# Structure-guided optimization of *N*-sulfonylpiperidines toward potent multi-target anticancer agents

**DOI:** 10.1038/s41598-026-44109-z

**Published:** 2026-04-13

**Authors:** Al Ghazali S. Al Jazairi, Walid E. Elgammal, Mahmoud Basseem I. Mohamed, Mohamed A. Seleem, Mahmoud S. Bashandy

**Affiliations:** 1 Chemistry Department, Faculty of Science (Boys), Al-Azhar University, El-Nasr Road, Nasr City, Cairo, 11884 Egypt; 2 Department of Pharmaceutical Organic Chemistry, Faculty of Pharmacy, Al- Azhar University, Cairo, 11884 Egypt

**Keywords:** *N*-Sulfonylpiperidine, Thiazolidinone derivatives, Anticancer agents, Apoptosis, VEGFR-2 inhibitors, EGFR inhibition, Topoisomerase II, Biochemistry, Cancer, Chemical biology, Chemistry, Computational biology and bioinformatics, Drug discovery

## Abstract

Cancer poses a significant therapeutic challenge due to its multifactorial origin and resistance to conventional remedies. Our previous efforts identified *N*-sulfonylpiperidine derivatives as potent VEGFR-2 inhibitors (Compound A). Herein, we report a rational design, synthesis, and biological assessment of a new series of analogs to enhance anticancer efficacy through scaffold optimization and multi-target engagement. Strategic modifications on **A** included group repositioning, diversifying the terminal aromatic substituents, and linker replacement. Among the synthesized derivatives, compound **16** exhibited promising cytotoxic activities across three cell lines, in comparison with the lead compound **A** and vinblastine. Preliminary mechanistic studies confirmed that **16** induced G0/G1 cell cycle arrest and promoted early apoptosis in MCF-7 cells. Enzyme inhibition assays further revealed that our derivative acts as a dual inhibitor of VEGFR-2 and EGFR versus moderate activity against topoisomerase II. Molecular docking studies supported these findings by showing favorable binding orientations and interactions within the VEGFR-2 active site. The binding pattern of **16** showed key hydrogen bonding and hydrophobic contacts that enhance ligand affinity. Altogether, this work highlights the impact of these modifications and introduces **16** as a promising lead to develop multitargeted anticancer therapeutics that inhibit tyrosine kinases and DNA-processing enzymes.

**Supplementary Information:**

The online version contains supplementary material available at 10.1038/s41598-026-44109-z.

## Introduction

Cancer remains one of the leading causes of morbidity and mortality globally. According to the National Cancer Institute (NCI), an estimated 2 M new cases of cancer will be diagnosed in the US, with a 30% mortality rate^1^. The current situation poses a continuous challenge to drug discovery efforts due to its complex pathophysiology, resistance mechanisms, and side-effect profiles associated with conventional chemotherapies.^2–4^ Consequently, the pursuit of novel molecules to modulate critical cancer-associated pathways has become an urgent priority in oncology drug discovery.^5–8^.

One such pathway is angiogenesis, which is primarily driven by vascular endothelial growth factor receptor-2 (VEGFR-2), a key receptor tyrosine kinase involved in tumor vascularization, proliferation, and metastasis.^9–11^ Inhibition of VEGFR-2 has emerged as an effective strategy in limiting tumor growth, as demonstrated by FDA-approved agents such as sorafenib and sunitinib.^12–18^ Likewise, epidermal growth factor receptor (EGFR) is another receptor tyrosine kinase overexpressed in multiple epithelial cancers.^19–21^ In addition, DNA topoisomerase II (Topo II), the nuclear enzyme essential for DNA replication and repair, represents well-validated molecular targets in anticancer drug development.^22–24^. Targeted cancer therapy has been greatly aided by clinically licensed small-molecule tyrosine kinase inhibitors that target EGFR and VEGFR-2. These drugs also offer a well-established medicinal-chemistry foundation for scaffold building. Based on a 4-anilinoquinazoline core, first-generation EGFR medicines like gefitinib and erlotinib function as ATP-competitive inhibitors by means of crucial hydrogen-bond interactions with the hinge region (Met793) of the EGFR kinase domain. A solvent-exposed side chain that improves binding affinity and selectivity, a hydrophobic region that occupies the adenine pocket, and a heteroaromatic hinge-binding moiety are the usual components of their pharmacophore.^25,26^.

Similarly, structural characteristics of VEGFR-2 inhibitors like sunitinib and sorafenib are tailored for interaction within the ATP-binding region of VEGFR-2. Sunitinib has an indolinone scaffold that can form hydrophobic interactions and hinge binding within nearby lipophilic regions, whereas sorafenib has a diaryl urea linker that extends into the hydrophobic back pocket and forms dual hydrogen bonds with the hinge region^27,28^. A hinge-binding heterocycle, a suitable hydrogen-bonding linker, and terminal hydrophobic substituents for ideal pocket occupancy are the three fundamental pharmacophoric requirements for kinase inhibition that these inhibitors together emphasize. It is easier to evaluate the new scaffold’s uniqueness and expected pharmacophoric connections in relation to clinically proven EGFR and VEGFR-2 inhibitors when it is framed within this known structural paradigm.

**Fig. 1:**
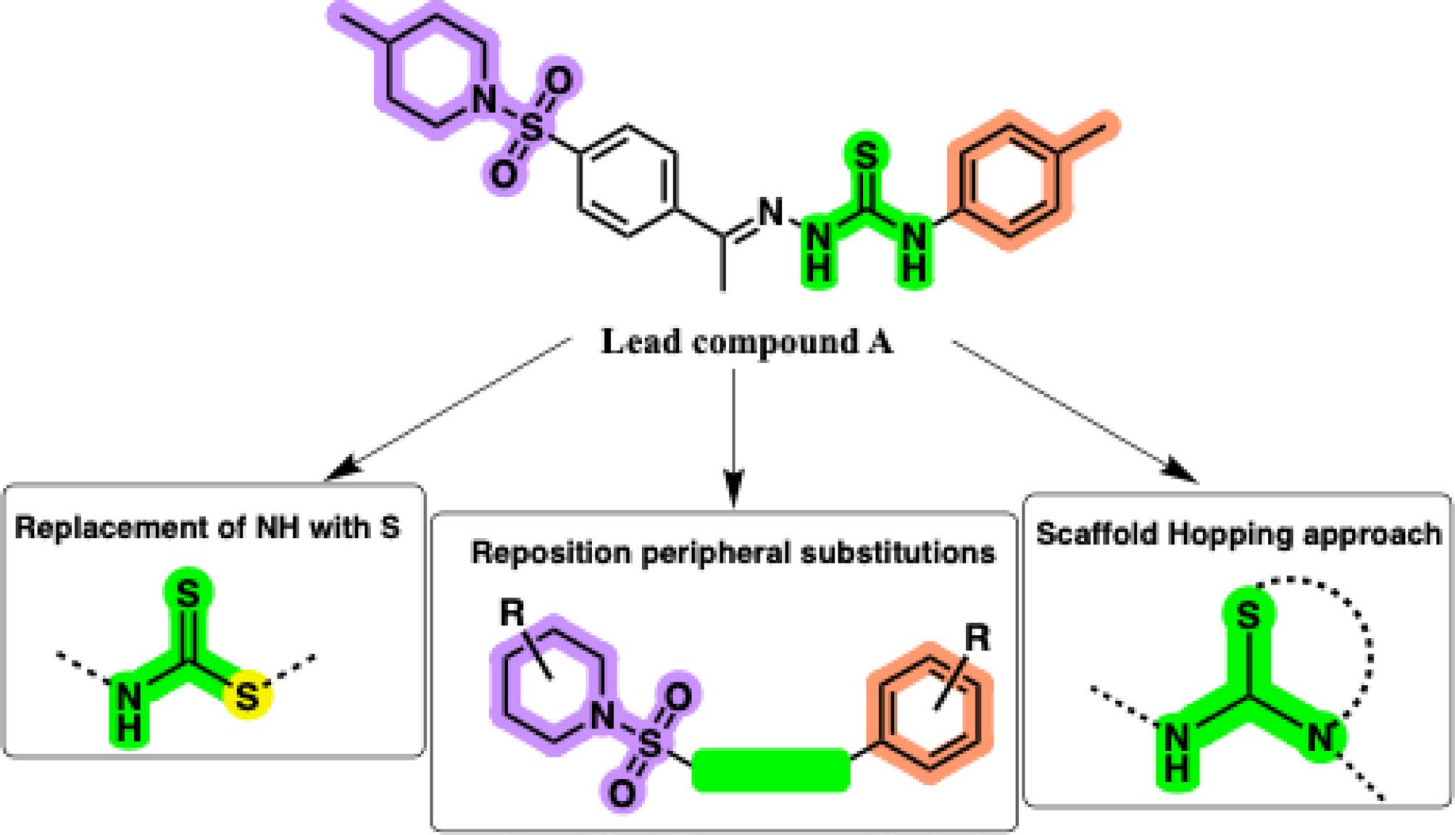
Work design.

In our previous work, we reported that a series of *N-sulfonylpiperidines* exhibited potent antiproliferative activity against various cell lines.^29^ Further investigations introduced compound **A** (Fig. 1) with promising VEGFR-2 inhibitory activity (IC₅₀ = 0.0554 µM), comparable to marketed drugs such as sorafenib and vinblastine. This category of molecules also induced G2/M cell cycle arrest, increased apoptotic populations, and demonstrated favorable in silico pharmacokinetic and toxicity profiles.

Prompted by these findings, we synthesized a new generation of *N*-sulfonylpiperidine-based derivatives with enhanced pharmacophoric features. These modifications aimed to expand the structure-activity relationship (SAR), improve drug-like properties, and probe multi-target interactions. In the current work, we report the synthesis, biological evaluation, and computational modeling of these novel derivatives, highlighting their potential as multitargeted anticancer candidates.^29,30^.

### Rationale of molecular design

Building on the promising activity of the lead molecule **A**, we sought to explore scaffold modifications that could enhance molecular rigidity and improve pharmacokinetic stability. The work design is summarized in Fig. 1. Structurally, compound **A** is composed of a thiourea-linked aniline moiety connected through a hydrazone bridge to a sulfonylated aryl-4-methylpiperidine fragment. We planned three fundamental strategies to improve the activity and physicochemical characteristics of these compounds. In the first strategy, we focused on modulating the piperidine ring by examining the impact of methyl group position by replacing the *p*-methyl isomer with *m*-methyl analogs. This strategy was aimed at measuring how subtle steric changes in the sulfonamide region may influence the biological activity of our compounds.

Next, the terminal aromatic moiety was diversified by introducing substituted anilines bearing *m*-methyl or *p*-methoxy to enhance π–π stacking, hydrogen bonding, and electronic compatibility with the active sites of the target proteins. Finally, the scaffold hopping approach was employed by replacing the flexible thiourea motif with a rigid fused dihydroimidazolone (hydantoin-like) system. The hydantoin and related systems are privileged structures known for their favorable pharmacokinetic and binding properties. This new scaffold was accompanied by electron-donating aryl substituents to improve target affinity. Through this multi-pronged, rational, and electronic modification, we aimed to enhance cytotoxic performance and generate more drug-like anticancer candidates.

## Results and discussion

### Chemistry

**Fig. 2:**
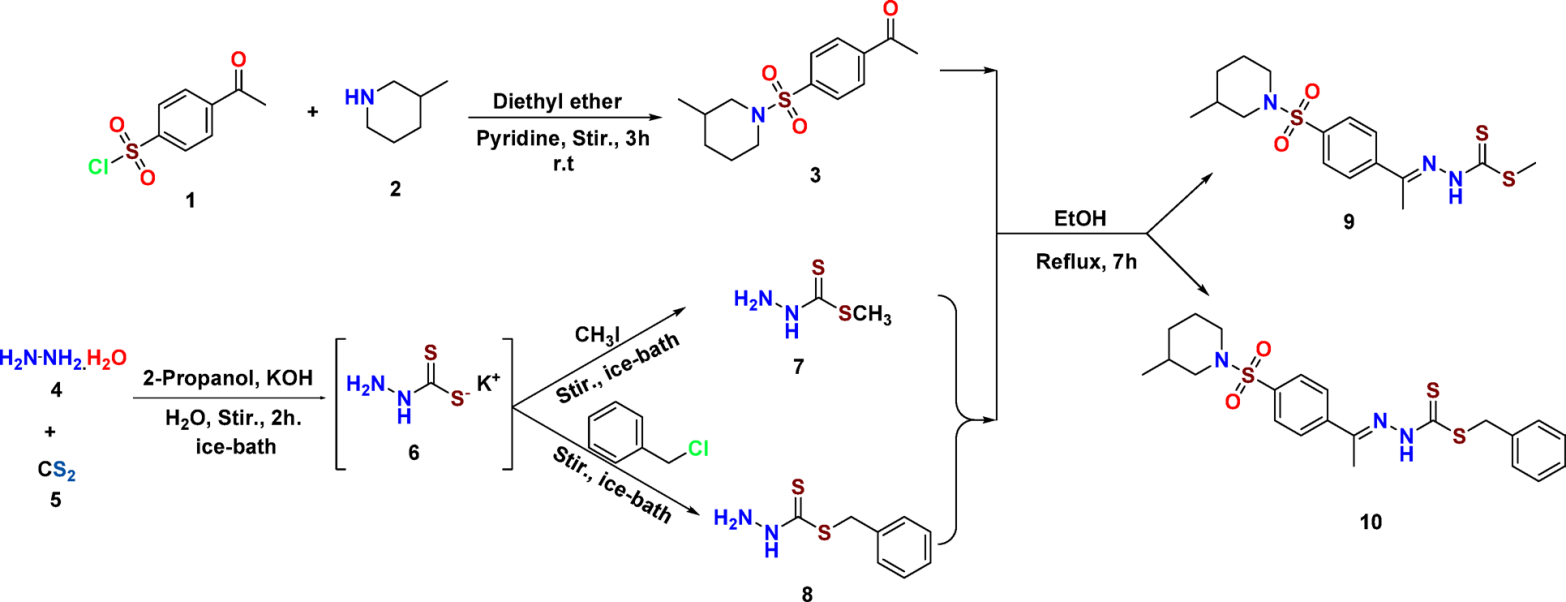
Preparation of the reagents and starting materials 3, 7, 8, and the methyl, benzyl thiourea hydrazone derivatives 9 and 10.

The establishment of methyl and benzyl thioester intermediates, **9** and **10**, was achieved via the ammonization of 4-acetylbenzenesulfonyl chloride with a heterocyclic aliphatic amine, such as β-pipecoline **2** (3-methyl piperidine), under basic conditions (pyridine),^31–34^, to afford the ketone derivative **3** by stirring for three hours at ambient temperature. This is then condensed with hydrazine derivatives **7** and **8** [prepared by means of hydrazinium hydroxide **4** plus methanedithione (CS₂, **5**) in an alcoholic medium of caustic potash (KOH)], affording intermediate **6**. Following that, an alkylation agent was added, like methyl iodide and/or benzyl chloride, resulting in reagents 7 and 8],^11,35–38^, ultimately yielding the corresponding molecules **9** and **10**, as shown in Fig. 2.

**Fig. 3:**
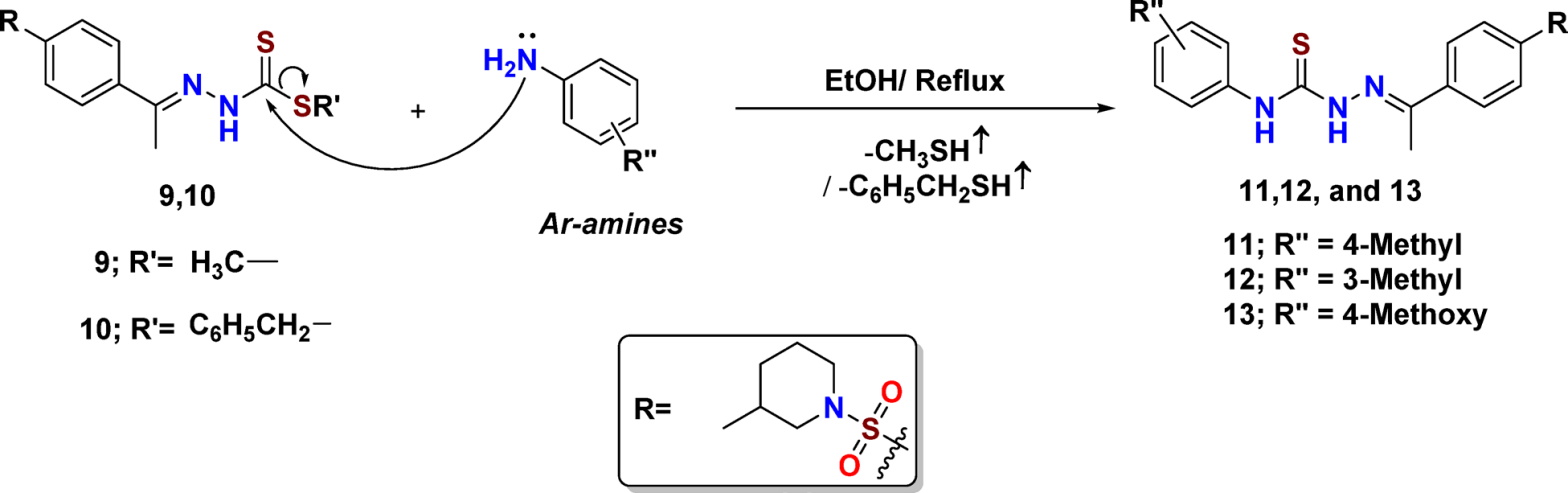
Synthetic pathway for preparing target thiosemicarbazide derivatives.

In order to confirm the structures of starting materials 9 and 10, we evaluated their chemical reactivity. This investigation focused on the role of methylsulfanyl and benzylsulfanyl groups as leaving groups in nucleophilic reactions. The most conventional and efficient method for synthesizing a thiosemicarbazone derivative involves a nucleophilic substitution between a methyl and/or benzyl thiol and an amine. This reaction is followed by the attack of primary aromatic amines such as para-toluidine, meta-toluidine, and anisidine on thioester intermediates 9 and 10 in an alcoholic solution heated under reflux until no methyl or benzyl mercaptan is detectable [tested using lab paper moistened with disodium pentacyanonitrosylferrate(II) dihydrate reagent (Na_2_[Fe(CN)_5_NO]. 2H_2_O), that produced a pink color].^29^ Also, the completion of the reaction was monitored by TLC using a mixture of two solvents, dichloromethane and methanol, at a ratio of 95:5, to afford unsymmetrical disubstituted thiourea derivatives 11–13 with impressive yields of 80–85% (Fig. 3).

**Fig. 4:**
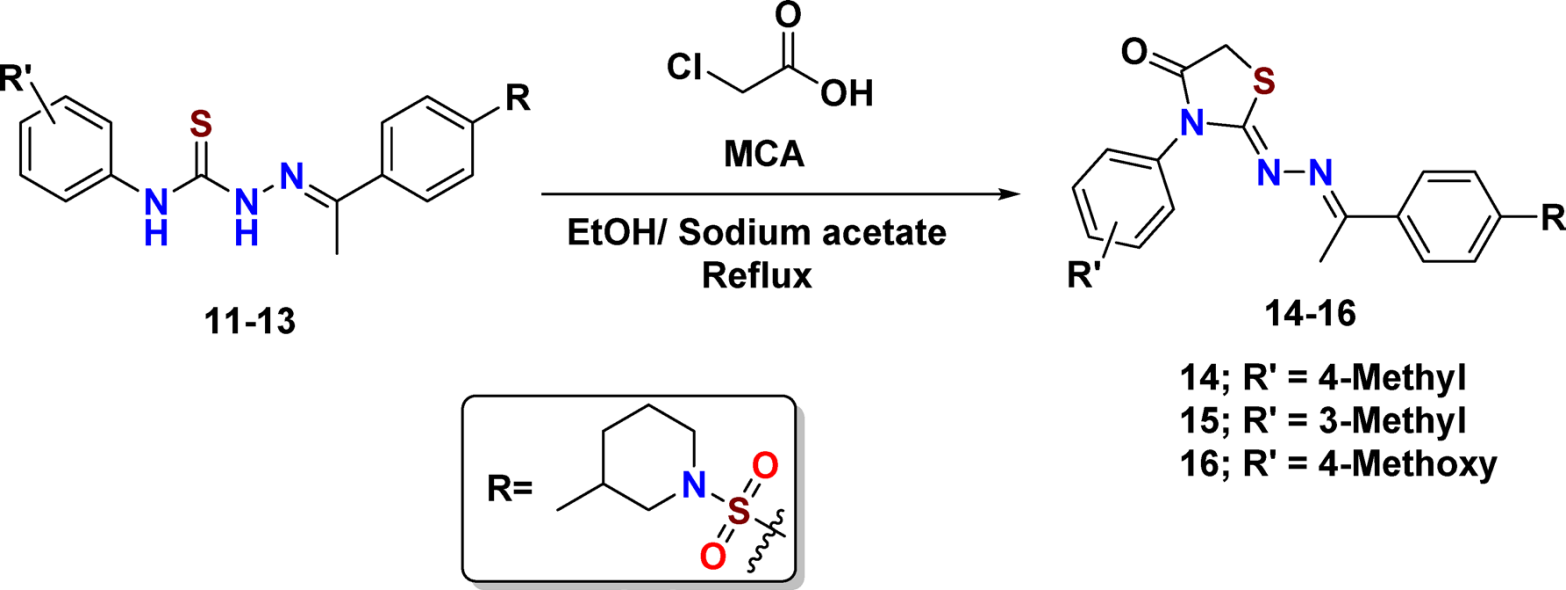
Synthetic pathway for preparing targets thiazolidin-4-one derivatives.

Eventually, the cyclocondensation of thiosemicarbazone precursors **11–13** with monochloroacetic acid (**MCA**) was carried out in boiling absolute ethanol, in conjunction with fused sodium acetate (NaOAc).^39^ After a duration of 7 h, this reaction effectively produced thiazolidinone derivatives **14–16** in moderate yields (Fig. 4). The initial materials exhaustion was monitored using TLC.

**Fig. 5:**
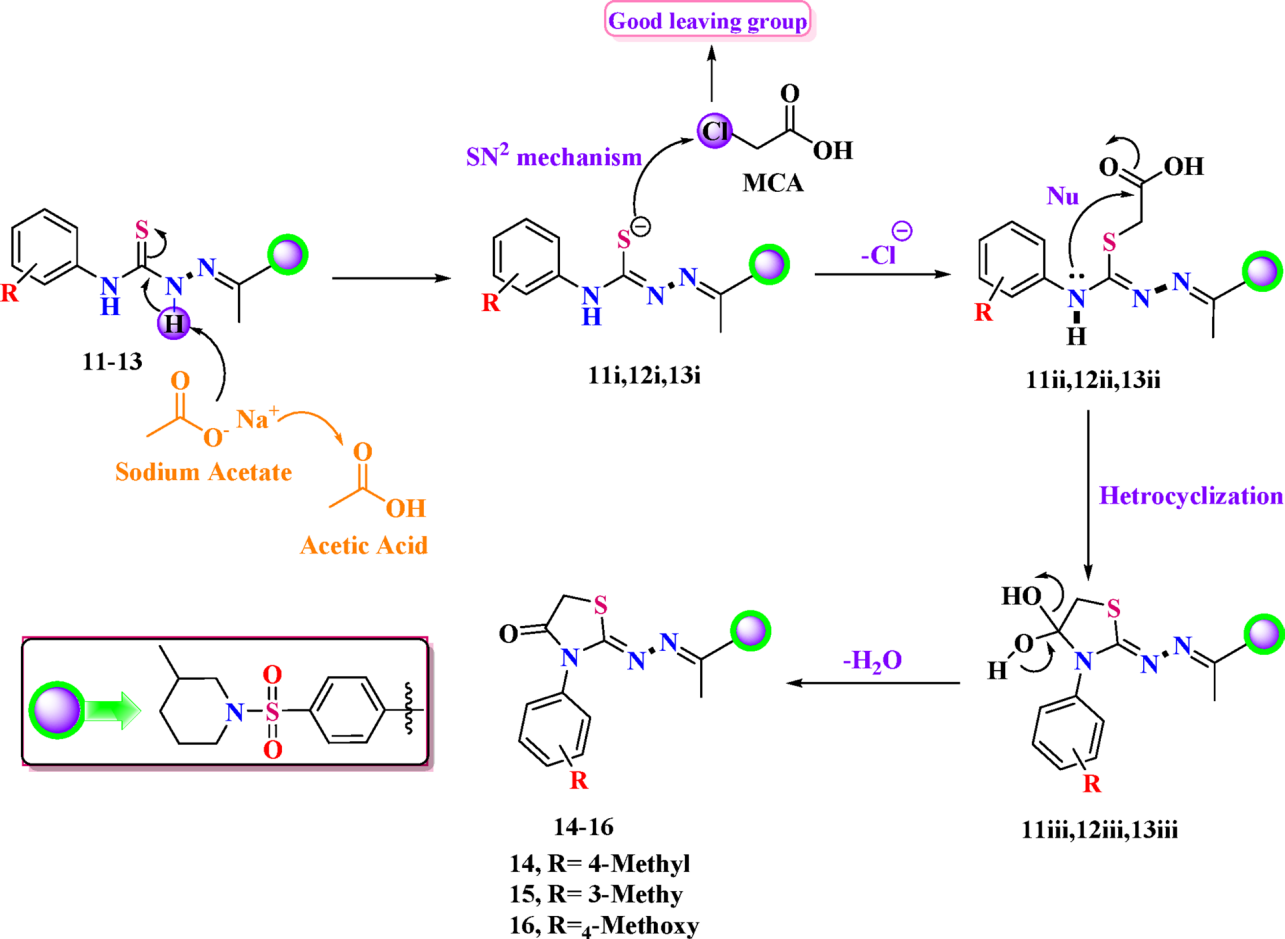
An offered machinery for forming a five-membered thiazolidinone ring.

The trustworthy mechanism for building thiazolidinones **14–16** could be epitomized in two steps. (i) An initial stage is the alkylation of the thiocarbonyl group (-C = S) of thiosemicarbazones **11–13** in their -SH construct (in thiolactim structure) in a mildly basic environment using anhydrous sodium acetate. After protonation of imino (NH) by sodium acetate, an intermediate in partially or fully thiol form was formed. This allowed S atoms to interact with the electrophilic center (CH₂-Cl) group of chloroacetic acid, which is more electrophilic than the COOH group, through the SN^2^ mechanism^40,41^. This step involves a nucleophilic attack by thiol on the electrophilic carbon of CH₂-Cl to remove the leaving group Cl^−^. This causes the S-alkylation of thiol and the formation of a new C-S bond. (ii). During the second stage, the intermediate underwent intramolecular cyclization, resulting in the formation of a heterocyclic ring through the secondary amino group (NH) attacking the carbonyl carbon. Subsequently, a water molecule was eliminated, facilitating the formation of five-membered thiazolidinones,^42^ (Fig. 5).

## Biological investigation

### Anticancer assay

At the outset of this study, the anti-proliferative activities of our compounds were determined using three human cancer cell lines: breast (MCF-7), colon (HCT-116), and liver (HepG_2_) in an MTT assay^43–45^. Dysregulation of EGFR-mediated signaling pathways has been reported to contribute to tumor progression and survival in these kinds of cancers.^46,47^ Although VEGFR-2 is predominantly expressed in endothelial cells, VEGF-driven signaling has been implicated in tumor proliferation and angiogenesis-related mechanisms in these cancer types.^48^ Therefore, the designated cell lines introduce relevant preliminary models to evaluate the antiproliferative impact of our compounds. Importantly, the proposed mechanism is further supported by the direct in vitro EGFR and VEGFR-2 enzymatic inhibition assays presented in this study. The marketed drug vinblastine was used as a positive control in the experiment. Data from this assay were demonstrated as the concentration that produced a 50% inhibition of cell growth, 24 h post-treatment (IC_50_), compared to positive and untreated controls, as summarized in Table 1.

First, the intermediate compound **3**, with a peripheral acetyl group, showed a very poor cytotoxic effect against the three cell lines. This observation highlighted the essentiality of the thiourea part as a pharmacophoric moiety, as indicated by our earlier work.^29^ The first set of molecules (**9** ~ **13**) did not display any promising activity against the three cell lines when compared with vinblastine and the lead compound **A**. Unexpectedly, repositioning the methyl group on the piperidine ring in compound **11** resulted in a substantial drop in its cytotoxic activity relative to the 4-methyl isomer, compound **A**. Modifications at the terminal aryl region restored partial activity. For instance, compound **12**, featuring a 3-methylpiperidine sulfonamide and an m-methylphenyl group, demonstrated improved cytotoxic activity compared to compound **11** but was still less potent than the parent isomer **A**. This observation is likely due to enhanced hydrophobic and electronic interactions.

Notably, scaffold-hopped congeners where the thiourea linker was incorporated in a fused dihydroimidazolone ring showed significant improvements in the cytotoxic activity. Among these compounds, derivative **16**, which bears a *p*-methoxy phenyl side chain, showed the most promising activity against the three cell lines when compared with the lead compound **2** and vinblastine (Table 1). Replacement of the *p*-methoxy phenyl moiety with *p*-methyl phenyl (**14**) or *p*-methyl phenyl (**15**) yielded two compounds with moderate cytotoxic effect. These results may demonstrate the effect of conformationally rigid, pharmacophore-rich scaffolds in the development of novel anticancer agents.

**Table 1:** Cytotoxic activity of the target compounds against MCF-7, HepG-2, and HCT-116 cell lines. Data are reported as the mean ± SD of three independent experiments in triplicate.

Cp.ID	Cytotoxicity IC50 (µM)		
	MCF-7	HCT116	HepG-2
A	4.43 ± 0.10	3.94 ± 0.10	3.76 ± 0.32
3	18.43 ± 0.9	23.78 ± 1.03	19.39 ± 1.2
9	31.48 ± 0.04	24.66 ± 0.89	20.0 ± 0.8
10	11.63 ± 0.82	15.02 ± 0.92	19.06 ± 0.85
11	22.26 ± 0.94	17.02 ± 0.87	15.73 ± 0.16
12	11.39 ± 0.18	14.41 ± 0.13	8.48 ± 0.70
13	12.98 ± 0.07	11.09 ± 0.23	9.60 ± 0.12
14	17.91 ± 0.77	14.84 ± 0.75	10.05 ± 0.71
15	14.02 ± 0.12	11.90 ± 0.25	6.79 ± 0.11
16	**3.78 ± 0.15**	**4.27 ± 0.52**	**4.79 ± 0.81**
Vin	4.6 ± 0.21	4.03 ± 0.63	8.5 ± 0.11

## In vitro cancer-related target inhibition analysis

### Cell apoptosis

**Fig. 6:**
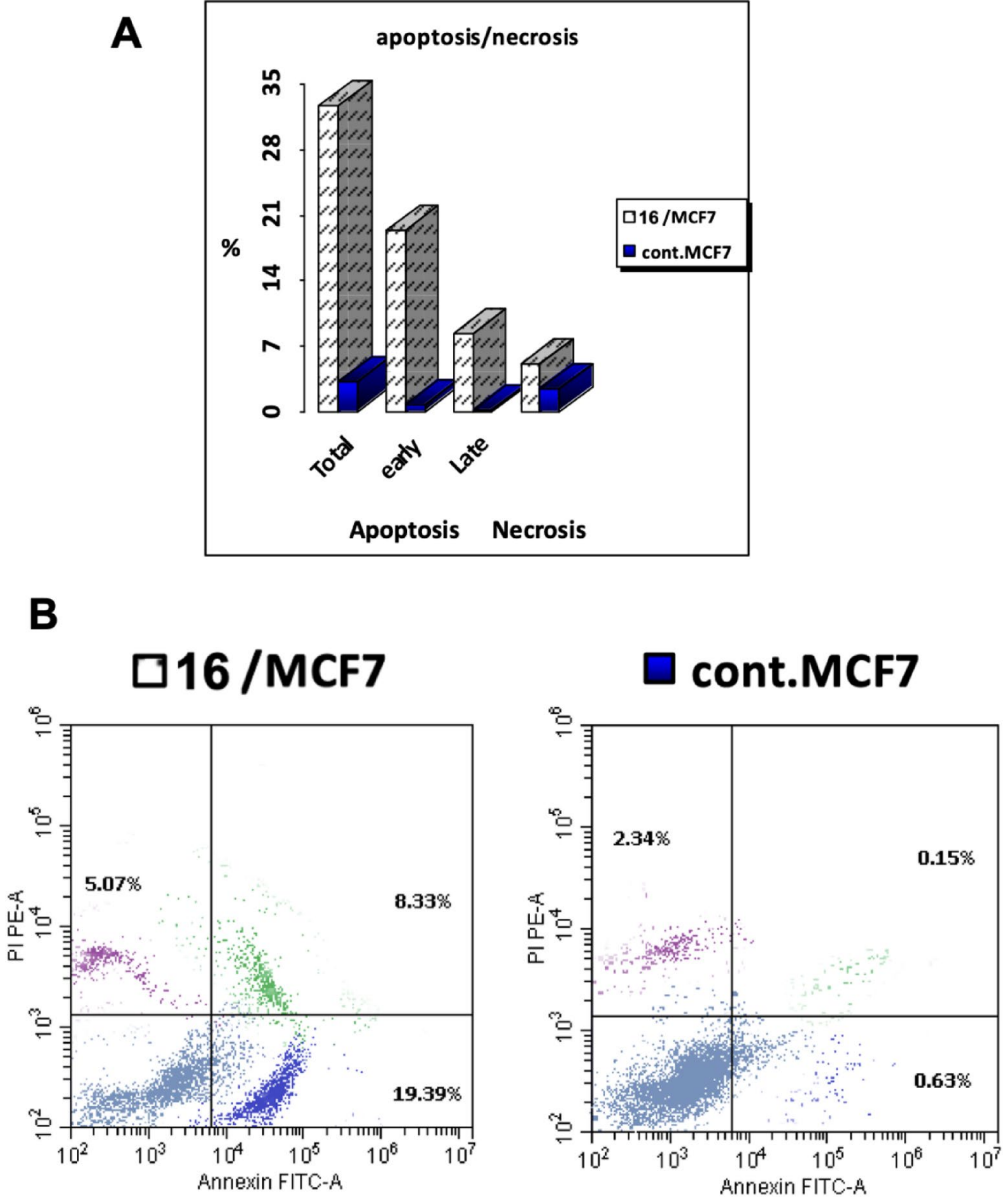
Annexin V–FITC/propidium iodide (PI) apoptosis analysis of MCF-7 cells following treatment with compound 16. **A** Quantitative representation of total apoptosis, early apoptosis, late apoptosis, and necrosis percentages in MCF-7 cells treated with compound 16 at its IC₅₀ concentration (3.78 µM) for 24 h (compared with untreated control cells). **B** Flow cytometry dot plots of Annexin V–FITC. Viable cells (Annexin V−/PI−) appear in the lower left quadrant, early apoptotic cells (Annexin V+/PI−) in the lower right quadrant, late apoptotic cells (Annexin V+/PI+) in the upper right quadrant, and necrotic cells (Annexin V−/PI+) in the upper left quadrant. The data are reported as the mean ± SD of three independent experiments in triplicate.

The binding of Annexin V to phosphatidylserine (PS) exposed on the external leaflet of the plasma membrane during apoptosis is widely used as an indicator of apoptotic cell death.^49^ In order to investigate the mechanism underlying the cytotoxicity of **16**, we performed an Annexin V-FITC/PI dual staining assay using MCF-7 cells. We used this flow cytometric analysis to distinguish between viable, early apoptotic, late apoptotic, and necrotic MCF-7 cell populations after treatment. Results from this assay are summarized in Fig. 6.

The results revealed that our molecule induced a significant apoptotic response in the treated cells (Fig. 6A). The total apoptosis rate was 32.79% (19.39% early apoptotic cells and 8.33% late apoptotic cells), along with a necrosis rate of 5.07% (Fig. 6B). In contrast, the control group showed only 3.12% total apoptosis (0.63% early, 0.15% late) and 2.34% necrosis. These findings suggest that our compound triggers apoptosis as the primary mode of cell death, which was consistent with its potent cytotoxic profile (Table 1). The higher apoptotic population in treated MCF-7 cells, especially in the early apoptotic phase, supports the hypothesis that **16** may exhibit its cytotoxic effect through apoptosis-regulatory signaling pathways, which involve mitochondrial dysfunction or caspase activation.

### Cell cycle analysis

**Fig. 7:**
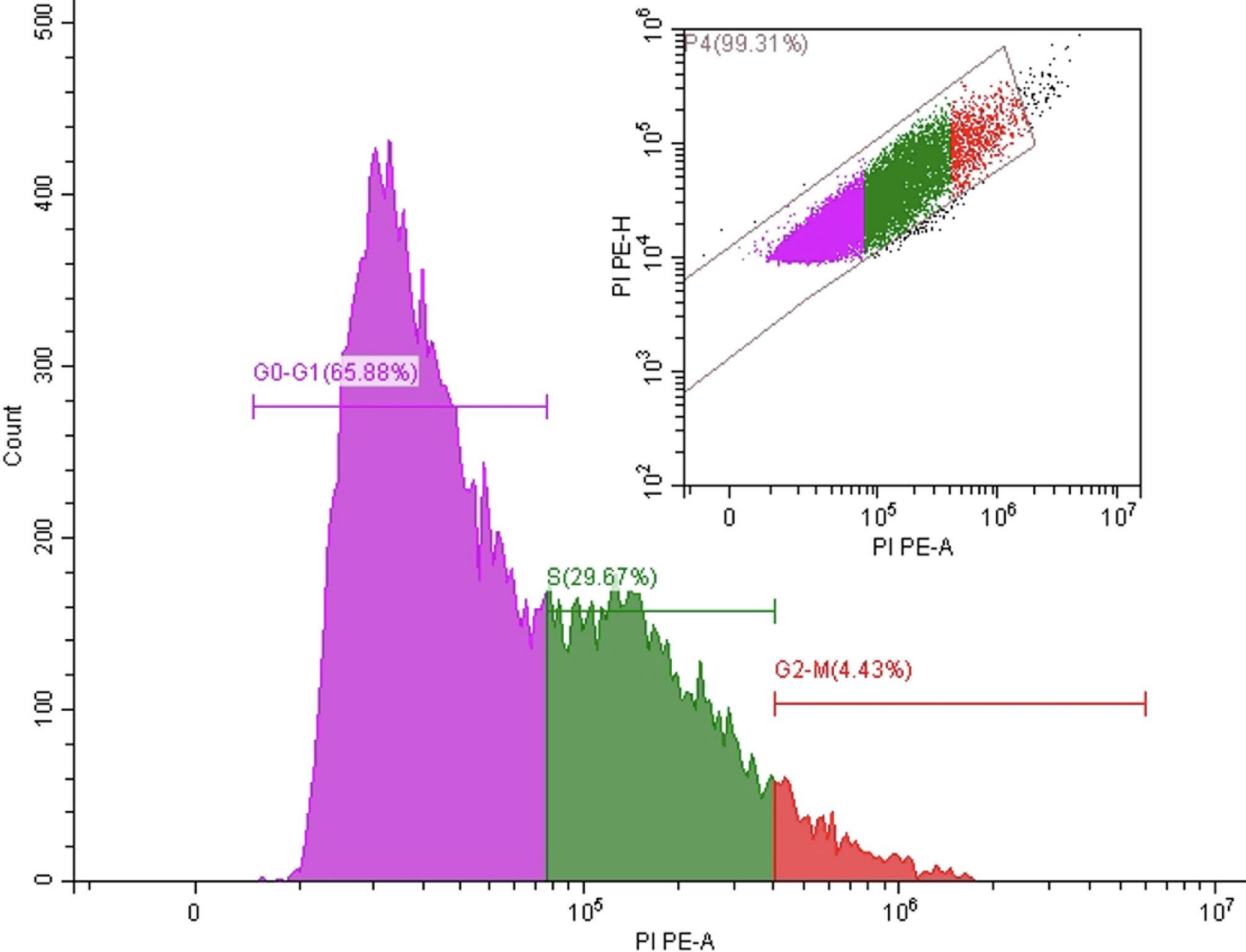
Dot plot of propidium iodide (PI) staining of the MCF-7 cell line treated with compound **16** at a concentration of 3.78 µM. After 24 h, the cells were fixed, stained with PI, and analyzed using flow cytometry. The percentages of cells in Sub-G1, G0/G1, S, and G2/M phases were quantified. Untreated cells served as the control. Data are reported as the mean ± SD of three independent experiments in triplicate.

Next, the further effect of **16** on regulating cell cycle distribution analysis was investigated using the Propidium Iodide Flow Cytometry Kit assay (Fig. 7). The results showed a significant alteration in cell cycle progression in MCF-7 cells treated with **16** compared to typical control profiles. Specifically, treatment with our compound resulted in the accumulation of 65% of the treated cells in the G0/G1 phase (Fig. 7). This pattern indicates that compound **16** induces a prominent G0/G1 phase arrest and obstructs the initiation of DNA synthesis and subsequent cell cycle progression. Substantial reduction in the G2/M population (4%) suggests that the **16** may also interfere with early cell cycle checkpoints. This interference is potentially through inhibition of cyclin-dependent kinases (CDKs) or upregulation of cell cycle inhibitory proteins such as p21 or p27. On the other hand, increased S-phase cell content (29) may reflect partial DNA synthesis stalling or a compensatory response due to G1 checkpoint disruption.

These results are in alignment with the Annexin V apoptosis assay, which showed a high proportion of early apoptotic cells, indicating that **16** not only halts cell cycle progression but also drives cells toward apoptosis.

### In vitro enzyme inhibition of EGFR, VEGFR-2, and topoisomerase II

**Fig. 8:**
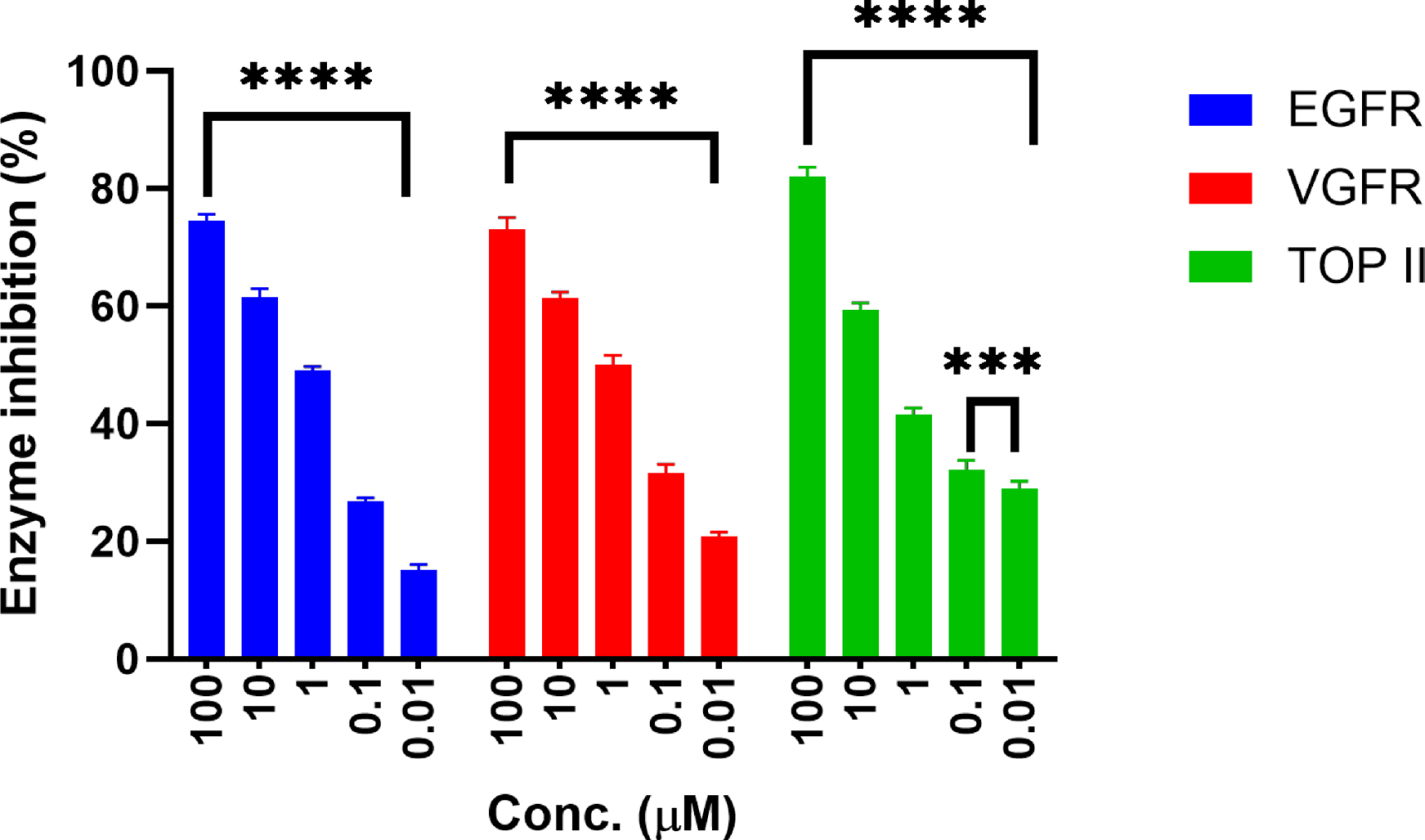
Enzymatic inhibition profile of **16** against EGFR (blue), VEGFR-2 (red), and Topoisomerase II (green) at five concentrations (100 − 0.01 µM). Data are presented as mean ± SD (*n* = 3). Statistical comparisons were performed using one-way ANOVA; each symbol represents the average of an experiment done in triplicate for the significant identification between groups (***p < 0.001, ****p < 0.0001).

Next, we performed an in vitro enzyme inhibition assay to investigate the molecular targets responsible for the reported cytotoxic effect of molecule **16**. In this assay, we evaluated the inhibitory activity of our compound against EGFR, VEGFR-2, and topoisomerase II, the clinically relevant cancer-related enzymes. Across a concentration range of 0.01 to 100 µM, as summarized in Fig. 8. The results revealed a prominent inhibition of EGFR and VEGFR-2 in a dose-dependent manner. At the highest use concentration (100 µM), the mean inhibition% reached 74.5% and 73.0%, respectively. Conversely, the inhibition of topoisomerase II at 100 µM was predominantly higher than the other two enzymes (81.9%). However, the overall dose-response curve indicated a considerably weaker potency.^50–54^.

Curve fitting of the inhibition data yielded IC_50_ values of 0.98 µM for VEGFR-2, 1.01 µM for EGFR, and 1.94 µM for Topoisomerase II. This data may confirm that compound **16** is a dual potent kinase inhibitor. The higher IC_50_ against topoisomerase II also suggests that **16** exhibits preferential inhibition toward receptor tyrosine kinases over nuclear enzymes involved in DNA topology.

## Molecular docking

Data from this assay correlate well with the previously observed cytotoxic and apoptotic effects of compound **16** against MCF-7 cells and support a mechanism of action involving dual blocking of EGFR and VEGFR-2. It also highlighted a potential interference with DNA-related targets at higher concentrations. This dual-target profile may reinforce the therapeutic promise of **16** as a kinase-focused anticancer lead compound.

**Fig. 9:**
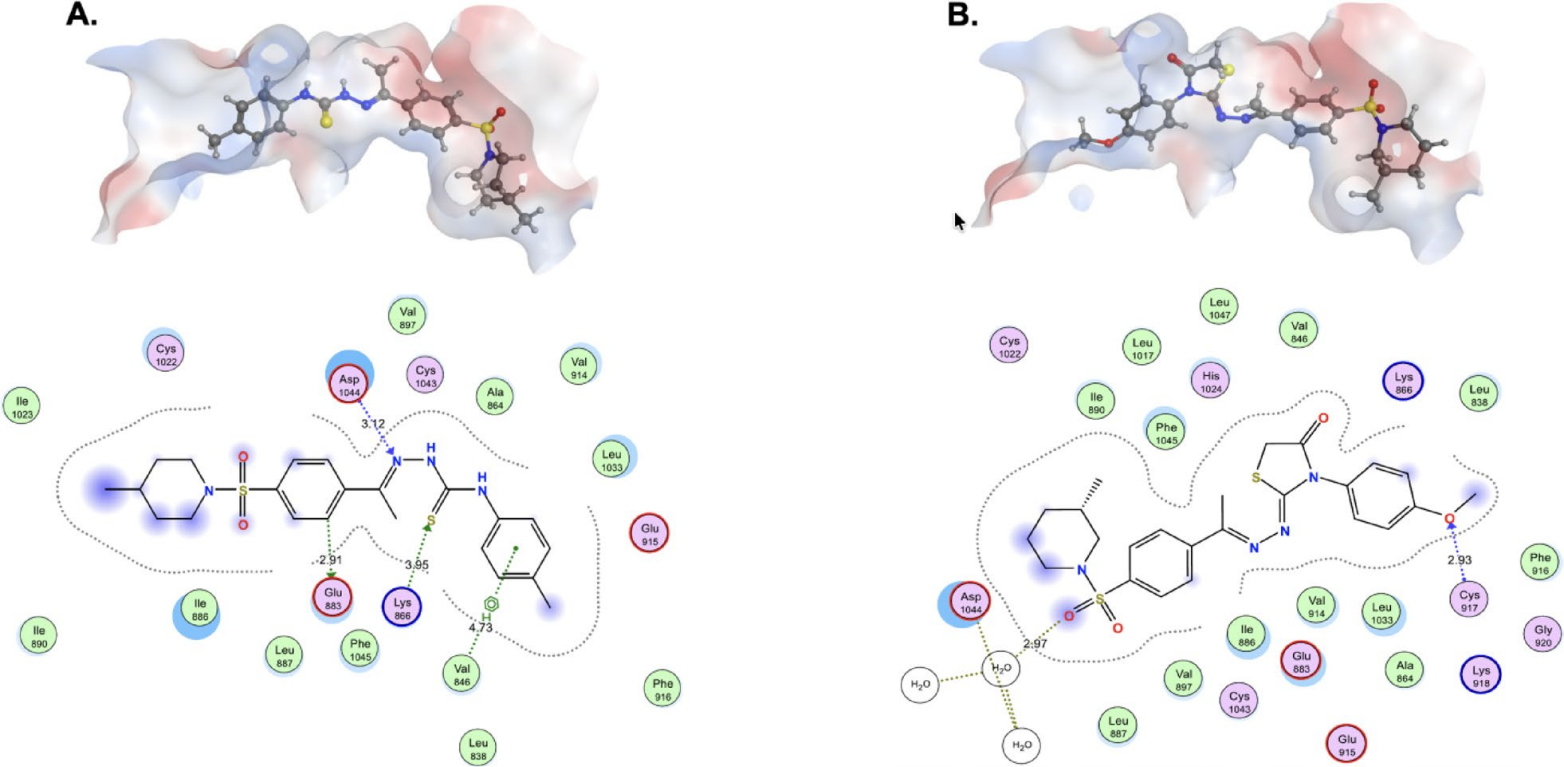
3D and 2D interactions of compounds **A** (**A**) and compound **16** (**B**) with VEGFR-2 (PDB ID: 2OH4). Docking was performed using MOE software. The key interacting residues are displayed as circles. Hydrogen bonds are represented as dashed lines, and hydrophobic interactions are shown as violet surfaces.

In order to identify the possible mechanism of action of compound **16**, we performed an in silico docking study. Compound 16 was docked against the reference molecule A inside VEGFR-2 (PDB ID: 2OH4). Docking of compound **A** into the VEGFR-2 active site (Fig. 9A) showed consistent hinge region engagement and interactions with the catalytic pocket residues. The sulfonyl moiety forms hydrogen bonds with Lys866 and/or Cys1022, which stabilizes the ligand orientation. In addition, the hydrazone–thiourea fragment participates in hydrogen bonding with two key residues near the DFG motif (Asp1044 and Glu883). Hydrophobic contacts are observed with Val846, Leu838, Leu1033, and Phe916, which frame the inhibitor within the lipophilic pocket.

In compound **16**, we observed that the fused dihydroimidazolone scaffold enforces a rigid conformation that aligns the pharmacophoric groups more effectively with the hinge region (Fig. 9B). We also observed a hydrogen bond interaction between the methoxyphenyl oxygen and Cys917, while the sulfonyl moiety maintains contact with Lys866 and Cys1022, as indicated in the reference molecule **A**. Notably, the piperidine nitrogen provides an extra stabilization point by engaging in a water-mediated hydrogen bonding with Asp1044. The ligand also benefits from extensive hydrophobic contacts with Leu838, Val846, Phe916, Ile890, and Leu1033, complemented by proximity to Glu915 and Glu883. These residues are known to be important for ligand recognition. These additional polar and hydrophobic interactions, combined with reduced conformational flexibility, are likely to contribute to the improved docking score and may explain the enhanced VEGFR-2 inhibitory activity observed for compound **16** in vitro.

## Quantum chemical analysis of reactivity indices for compound 16: insights into enhanced VEGFR-2 inhibition

Quantum chemical descriptors derived from Density Functional Theory (**DFT**) calculations provide a powerful framework for understanding the relationship between molecular electronic structure and biological activity. In this study, we employ the M05-2X-D3/6-31G** level of theory to compute key reactivity indices, including frontier molecular orbital energies, chemical hardness, softness, electronegativity, and electrophilicity for compound **16**, a potent multi-target anticancer agent. These indices are critically analyzed to elucidate the electronic factors underlying its enhanced inhibitory activity against VEGFR-2, a key target in anticancer drug design.

The HOMO energy (–0.211 au.) reflects the electron-donating ability of compound **16**. A relatively high HOMO value indicates a tendency to donate electrons to electron-deficient regions of the target protein, such as the electrophilic pockets in the VEGFR-2 active site. The LUMO energy (–0.0569 au.) suggests a moderate electron-accepting capacity, which may facilitate interactions with nucleophilic residues. The narrow energy gap (ΔƐ = 0.1541 au.) is particularly significant. A small ΔƐ is indicative of high chemical reactivity and polarizability, which often correlates with enhanced biological activity. This low gap implies that compound **16** can readily undergo charge transfer interactions with the enzyme’s active site, stabilizing the inhibitor–receptor complex. The computed hardness (η = 0.07705 au.) is low, while softness (σ = 12.98 au.) is high. According to Pearson’s Hard-Soft Acid-Base principle, soft molecules prefer soft reaction sites. The high softness of **16** suggests a propensity for strong, covalent-like interactions with soft nucleophilic residues in VEGFR-2, such as cysteine (Cys917) or lysine (Lys866), which aligns with docking results showing hydrogen bonding and hydrophobic contacts. The electronegativity (χ = 0.13395 eV) reflects the molecule’s ability to attract electrons. The moderate χ value indicates a balanced electron distribution, which may optimize both hydrogen-bond acceptance and donation. The electrophilicity index (ω = 0.1164 eV) quantifies the molecule’s tendency to accept electrons. A higher ω is associated with stronger electrophilic character, which in kinase inhibitors often correlates with binding to ATP-binding sites rich in electron-donating residues. The moderate ω of **16** suggests it can act as an effective electrophile without being overly reactive, reducing potential off-target effects. The combination of a low energy gap, high softness, and balanced electrophilicity positions compound **16** as an efficient inhibitor of VEGFR-2. The narrow ΔƐ facilitates charge transfer during binding, while high softness enables adaptive interactions with the flexible hinge region of VEGFR-2. Molecular docking studies (manuscript Fig. 4) confirm that **16** forms hydrogen bonds with Cys917 and Lys866 and hydrophobic contacts with Leu838 and Val846. The computed electronic properties support these interactions: the soft, polarizable electron cloud of **16** can engage in π–π stacking and van der Waals contacts, while its moderate electrophilicity allows for stable hydrogen-bond networks.

## Frontier molecular orbital and molecular electrostatic potential analysis of compound 16, electronic origins of charge transfer, and VEGFR-2 inhibition

**Fig. 10:**
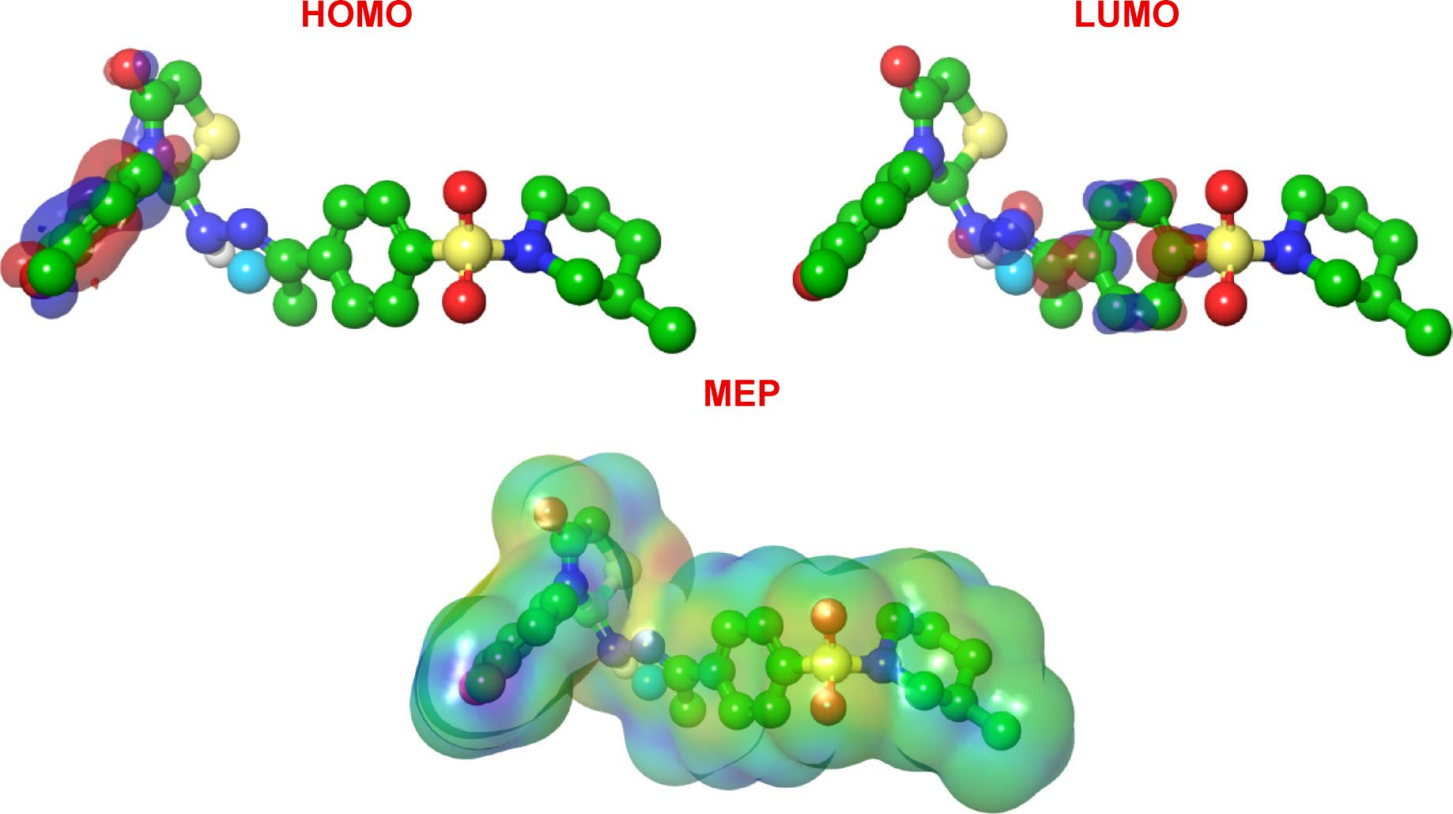
FMO and MEP for the most active **16**.

The HOMO of compound **16** is predominantly localized over the conjugated π-system encompassing the p-methoxyphenyl ring and the adjacent hydrazone-thiazolidinone scaffold. This distribution indicates that these regions are electron-rich and serve as the primary sites for nucleophilic attack or electron donation. Specifically, the HOMO density over the methoxy-substituted aromatic ring suggests its role in π–π stacking interactions with aromatic residues (Phe916) in the VEGFR-2 active site. The contribution from the thiazolidinone carbonyl and imine groups further highlights their potential as hydrogen-bond acceptors (Fig. 10). The LUMO is largely localized on the sulfonylpiperidine moiety and the electron-deficient thiazolidinone core. This localization indicates that these regions are electrophilic and likely to accept electron density from nucleophilic protein residues. The LUMO distribution over the sulfonyl group aligns with its known role in forming hydrogen bonds with Lys866 and Cys1022 in VEGFR-2, as observed in docking studies.

The spatial separation of HOMO and LUMO densities across distinct regions of the molecule facilitates intramolecular charge transfer (ICT), which enhances molecular polarizability and stabilizes inhibitor–receptor complexes. The HOMO→LUMO excitation represents a π→π* transition with possible charge transfer character from the donor (methoxyphenyl) to the acceptor (sulfonyl-thiazolidinone) regions. This ICT character is consistent with the narrow energy gap (ΔƐ = 0.1541 eV) previously calculated, which promotes favorable electronic coupling with the enzyme’s active site. In biological terms, this charge transfer capability enables **16** to interact dynamically with both hydrophobic and polar residues, optimizing binding affinity and selectivity.

## Molecular electrostatic potential (MEP) analysis

The MEP map of compound **16** reveals distinct regions of electrostatic potential corresponding to its functional groups. Red regions (negative potential) are localized over the sulfonyl oxygens and the thiazolidine one carbonyl oxygen, indicating strong hydrogen-bond accepting capacity. Blue regions (positive potential): Observed around the protonated piperidine nitrogen and the hydrazone NH groups, reflecting hydrogen-bond donating ability. Green regions (neutral potential) are associated with the aromatic rings and aliphatic chains, indicative of hydrophobic and van der Waals interaction zones. The MEP distribution correlates directly with the docking-predicted interactions. The negative potentials over sulfonyl oxygens complement the positive residues (Lys866, Cys1022) in VEGFR-2. The positive potential near the piperidine nitrogen supports water-mediated hydrogen bonding with Asp1044. The neutral aromatic surfaces align with hydrophobic subpockets lined by Val846, Leu838, and Leu1033.

The synergy between FMO distributions and MEP patterns provides a comprehensive electronic rationale for the inhibitory potency of **16**. The HOMO–LUMO separation facilitates electron donation from the methoxyphenyl group to the enzyme’s π-system while accepting electron density into the sulfonyl–thiazolidinone motif, stabilizing transition-state interactions. The MEP surface mirrors the active site electrostatic landscape of VEGFR-2, ensuring optimal hydrogen-bond networks and hydrophobic packing. The rigid dihydroimidazolone scaffold enforces a planar arrangement that maximizes orbital overlap and electrostatic alignment, reducing entropic penalty upon binding.

## In silico ADME and drug-likeness assessment of compounds A and 16

The predicted ADME profiles indicate that compounds **A** and **16** exhibited adequate drug-like characters. Compound **A** fully complies with Lipinski’s Rule of Five, while compound **16** shows a single borderline violation (Mwt 500.63 g/mol), which remains within the acceptable tolerance range (0–1 violation). Both compounds display suitable permeability potential represented by favorable lipophilicity (cLogP ≈ 3.7), acceptable hydrogen bonding capacity, and TPSA values below 140 Å²,

Neither compound is predicted to permeate the BBB, which may reduce CNS related adverse effects. In addition, both molecules are predicted to be non-substrates of P-glycoprotein, potentially minimizing efflux-related resistance. Overall, the in silico analysis supports the drug-like behaviour of our scaffold, with compound **16** remains within acceptable medicinal chemistry space for further optimization (Table 2).

**Table 2:** In silico ADME and drug-likeness prediction of **A** and **16**. Physicochemical descriptors, Lipinski parameters, and pharmacokinetic properties were calculated using the SwissADME web server.

Parameter	A	16	Recommended range
Molecular weight (g/mol)	444.61 g/mol	500.63 g/mol	< 500
cLogP (consensus)	3.71	3.73	1–5
H-bond donors (HBD)	2	0	≤ 5
H-bond acceptors (HBA)	4	7	≤ 10
Topological polar surface area (TPSA, Å²)	114.27 Å^2^	125.32 Å^2^	< 140
Rotatable bonds	7	6	< 10 (preferred)
Lipinski violations	0	1	0–1 acceptable
GI Absorption	High	Low	High preferred
BBB permeation	No	No	Optional (depends on indication)
P-gp substrate	No	No	No preferred

To preliminarily evaluate chemical novelty and potential off-target liabilities, compounds A and 16 were submitted to SwissSimilarity for 2D/3D similarity screening against FDA-approved and bioactive compound libraries. Both molecules showed only moderate similarity to known kinase inhibitors, indicating that our *N*-sulfonylpiperidine–thiazolidinone scaffold occupies a relatively underexplored region of VEGFR-2/EGFR inhibitor space. A small subset of hits was associated with tyrosine kinase and topoisomerase-related agents, consistent with the experimentally observed dual kinase and moderate Topo II inhibition profiles. No highly similar analogs linked to undesirable off-target mechanisms were identified at standard similarity cutoffs, which supports the novelty and acceptable off-target risk of compounds A and 16 at this stage.

## Structure activity relationship

The cytotoxicity data presented in Table 1 for a series of *N*-sulfonylpiperidine derivatives against three human cancer cell lines (MCF-7, HepG-2, HCT-116) reveal clear trends that inform the structure-activity relationships underlying their anticancer potency. This analysis systematically deconstructs the impact of specific structural modifications, piperidine methylation patterns, terminal aromatic substitution, and scaffold rigidity on biological activity, with a focus on identifying the pharmacophoric elements essential for potent, multi-target inhibition. The acetyl **3** derivative shows poor activity (IC₅₀ > 18 µM), confirming that the thiourea-hydrazone bridge is critical for activity. The methyl/benzyl thioester intermediates **9–10** also exhibit weak activity, reinforcing that the free thiourea (or its cyclic bioisostere) is necessary for target interaction. The 4-methylpiperidine (**A**) is the lead (IC₅₀ ≈ 3.7–4.4 µM). The 3-methylpiperidine **11** isomer shows reduced activity (IC₅₀ ≈ 15–22 µM). Moving the methyl group from the 4- to the 3-position likely introduces unfavorable steric clashes or alters the sulfonamide conformation, disrupting optimal binding in the hydrophobic pocket of VEGFR-2/EGFR. While *m*-methylphenyl **12** improved activity (IC₅₀ ≈ 8–14 µM) when compared with compound **11**. The *meta*-methyl enhances hydrophobic contact with the active site without significant steric hindrance. The *p*-methoxyphenyl **13** compound shows further improvement (IC₅₀ ≈ 9–13 µM). This group provides electron-donating resonance and moderate hydrophobicity, likely stabilizing π–π stacking or hydrogen bonding (via ether oxygen). When *p*-methoxyphenyl is attached to a rigid dihydroimidazolone scaffold in **16**,** it** achieves the best activity (IC₅₀ ≈ 3.8–4.8 µM), comparable to compound **A** and superior to vinblastine in some cell lines.

Compounds **14–16** (thiazolidin-4-one derivatives) show marked improvement over their flexible thiourea precursors (**11–13**). The dihydroimidazolone **(**thiazolidinone**)** ring enforces conformational rigidity, reducing entropy loss upon binding and pre-organizing the pharmacophore for optimal target engagement. The most active compound, **16**, combines the most favorable aromatic substituent (***p*-methoxy**) with the rigid dihydroimidazolone core, yielding the highest potency. The rigid scaffold orients the sulfonylpiperidine and aromatic rings for simultaneous interaction with the hinge region and hydrophobic pockets of VEGFR-2/EGFR.

## Methodology

### Substances and equipment

All apparatus, chemicals, and reagents are detailed in Supplementary Information. All biological experiments in this work were conducted on established commercial human cancer cell lines (MCF-7, HCT-116, and HepG2). The cells were purchased from authenticated vendors (ATCC or equivalent accredited suppliers).

### Chemistry

#### Synthesis of 1-(4-((3-methylpiperidin-1-yl)sulfonyl)phenyl)ethan-1-one, 3

0.01 mol of a benzenesulfonyl chloride derivative and 0.01 mol of β-pipecoline were subjected to a reaction in anhydrous ethoxyethane with pyridine serving as a basic heterocyclic catalyst. Following this, the reaction contents underwent stirring at 25 °C for three hours. The resulting precipitate was collected by filtration, scrubbed using cold H_2_O, and then recrystallized from an appropriate solvent, providing the pure compound.

colorless crystals from ethanol (yield 75%), Rf = 0.75 (DCM/MeOH, 9:1 v/v), HPLC purity (area %): 97.84%, m.p. 107–109 °C, IR (KBr, υ_max_ cm^−1^): 3055 (C-H aromatic), 2931, 2870 (C-H aliphatic), 1689 (C = O (acetyl)), 1342, 1165 (SO_2_). ^1^H NMR (300 MHz, CDCl_3_) δ_H_ = 8.08 (d, *J* = 8.2 Hz, 2 H, AB-Ar-H), 7.85 (d, *J* = 8.3 Hz, 2 H, AB-Ar-H), 3.74–3.59 (m, 2 H, piperidinyl_(2)_), 2.66 (s, 3 H, CH_3_), 2.28 (td, *J* = 11.3, 2.8 Hz, 2 H, piperidinyl_(6)_), 1.94 (t, *J* = 10.7 Hz, 1H, CH, piperidinyl_(3)_), 1.72 (dq, *J* = 12.7, 3.4 Hz, 2 H, CH_2_, piperidinyl_(4)_), 1.70–1.55 (m, 2 H, piperidinyl_(5)_), 0.88 (d, *J* = 6.5 Hz, 3 H, CH_3_ of piperidinyl). ^13^C NMR (75 MHz, CDCl_3_) δ_C_ = 196.79 (acetyl carbon), 140.42, 139.85, 128.72, 127.73 (aromatic carbons), 77.42, 77.00, 76.58 (CDCl_3_), 53.03 (piperidinyl moiety-C_2_), 46.34 (piperidinyl moiety-C_6_), 31.86 (piperidinyl moiety-C_4_), 30.59 (piperidinyl moiety-C_3_), 26.78 (CH_3_-C = N-), 24.56 (piperidinyl moiety-C_5_), 18.81 (carbon of CH_3_-piperidinyl moiety). MS (*m*/*z*): 281 [%]: [M^+^, (32.38%)], Anal. Calcd for C_14_H_19_NO_3_S (281.11): C, 59.76; H, 6.81; N, 4.98; Found: C, 59.65; H, 6.72; N, 4.85%.

#### General synthetic procedure for the intermediates 7, 8

Methyl and benzyl hydrazinecarbodithioate derivatives 7 and 8 were successfully synthesized using hydrazine hydrate, carbon disulfide, methyl iodide, and benzyl chloride, adhering to the methodology outlined in a prior publication.^35–37,39^.

#### The prevailing synthetic method for the target derivatives 9, 10

A combination comprising 0.01 moles of ketone derivative 1 and 0.01 moles of either methyl hydrazinecarbodithioate 7 and/or benzyl hydrazinecarbodithioate 8, dissolved in 30 mL of anhydrous ethanol. Then, the resulting mixture was subsequently subjected to reflux with agitation for four hours. Once the condensation reaction was finished, as determined by TLC, the mixture was allowed to cool to approximately 25 °C. The resulting solid was then filtered and purified through recrystallization utilizing an appropriate solvent, ultimately leading to the separation of the desired final products, derivatives 9 and 10.

##### Methyl(R, E)-2-(1-(4-((3-methylpiperidin-1-yl)sulfonyl)phenyl)ethylidene) hydrazine-1-carbodithioate, 9

Pale yellow crystals from 1,4-dioxane (yield 73%), Rf = 0.62 (DCM/MeOH, 9:1 v/v) HPLC purity (area %): 100%, m.p. 173–175 °C, IR (KBr, υmax cm⁻¹): 3138 (NH), 3070 (C-H aromatic), 2950, 2872 (C-H aliphatic), 1612 (C = N), and 1339, 1161 (SO_2_); ^1^H NMR (400 MHz, DMSO) δ_H_ = 12.60 (s, 1H, NH, exchangeable by D_2_O), 8.08 (d, *J* = 8.6 Hz, 2 H, AB-Ar-H), 7.79 (d, *J* = 8.6 Hz, 2 H, AB-Ar-H), 3.52 (d, *J* = 5.6 Hz, 2 H, CH_2_, piperidinyl_(2)_), 2.54 (s, 3 H, SCH_3_), 2.44 (s, 3 H, CH_3_-C = N), 2.24 (tdd, *J* = 11.7, 6.4, 2.7 Hz, 2 H, CH_2_, piperidinyl_(6)_), 1.93 (d, *J* = 5.8 Hz, 1H, CH, piperidinyl_(3)_), 1.65–1.62 (m, 2 H, CH_2_, piperidinyl_(4)_), 1.46 (q, *J* = 12.4 Hz, 2 H, CH_2_, piperidinyl_(5)_), 0.84 (d, *J* = 6.4 Hz, 3 H, CH_3_-piperidinyl).^13^ C NMR (101 MHz, DMSO) δ_C_ = 201.26 (thiocarbonyl, C = S), 150.16 (-C = N-), 141.86, 139.92, 129.54, 128.17, 128.07, 127.73 (aromatic carbons), 53.09 (piperidinyl moiety-C_2_), 46.56 (piperidinyl moiety-C_6_), 31.71 (piperidinyl moiety-C_4_), 30.67 (piperidinyl moiety-C_3_), 24.60 (piperidinyl moiety-C_5_), 19.14 (CH_3_S), 17.55 (carbon of CH_3_-piperidinyl moiety). MS (*m*/*z*): 385.26 [%]: [M^+^, (15.92%)], Anal. Calcd for C_16_H_23_N_3_O_2_S_3_ (385.56): C, 49.84; H, 6.01; N, 10.90; Found: C, 49.72; H, 5.03; N, 10.78%.

##### Benzyl-2-(1-(4-((3-methylpiperidin-1-yl)sulfonyl)phenyl)ethylidene) hydrazine-1-carbodithioate, 10

white crystals from 1,4-dioxane (yield 70%), Rf = 0.5 (DCM/MeOH, 9:1 v/v), HPLC purity (area %): 95.47%, m.p. 185–187^°^C, IR (KBr, υmax cm⁻¹): 3428 cm⁻¹ (NH), 3063 (aromatic-CH), 2927, 2876 (C-H, aliphatic), 1602 (C = N), and 1339, 1157 (SO₂). ^1^H NMR (400 MHz, DMSO) δ_H_ = 12.65 (s, 1H, NH, exchangeable by D_2_O), 8.16 (d, *J* = 8.2 Hz, 3 H, AB-Ar-H and 1H of phenyl group), 7.83 (d, *J* = 8.2 Hz, 3 H, AB-Ar-H and 1H of phenyl group), 7.57–7.24 (m, 3 H, benzyl group), 4.51 (s, 2 H, CH_2_-benzyl), 3.54 (d, 2 H, CH_2_, piperidinyl_(2)_), 2.32 (s, 3 H, CH_3_-C = N), 2.25 (dd, *J* = 11.6, 2.8 Hz, 2 H, CH_2_, piperidinyl_(6)_), 1.95 (t, *J* = 10.7 Hz, 1H, CH, piperidinyl_(3)_), 1.78–1.59 (m, 2 H, CH_2_, piperidinyl_(4)_), 1.54–1.35 (m, 2 H, CH_2_, piperidinyl_(5)_), 0.85 (d, *J* = 6.4 Hz, 3 H, CH_3_-piperidinyl).^13^ C NMR (101 MHz, DMSO) δ_C_ = 199.26 (thiocarbonyl, C = S), 156.81 (-C = N-), 152.54, 149.72 (aromatic carbons), 141.92, 136.99, 130.95, 129.55 (benzyl carbons), 129.06, 128.19, 128.07, 127.89 (aromatic carbons), 53.11 (piperidinyl moiety-C_2_), 46.58 (piperidinyl moiety-C_6_), 31.72 (CH_2_-benzyl), 30.70 (piperidinyl moiety-C_4_), 24.62 (piperidinyl moiety-C_3_), 19.17 (piperidinyl moiety-C_5_), 16.61(CH_3_), 15.43 (carbon of CH_3_-piperidinyl moiety). MS (*m*/*z*): 461 [%]: [M^+^, (33.5%)], base peak; 137 (100%), Anal. Calcd for C_22_H_27_N_3_O_2_S_3_ (461.88 ): C, 57.24; H, 5.90; N, 9.10; Found: C, 57.13; H, 5.81; N, 8.97%.

#### Overall technique for the synthesis of desired derivatives 11–13

In an RBF outfitted with a condenser, a thioester derivative 9 and/or 10 (0.01 mol) and substituted aniline, for example, p-toluidine and/or m-toluidine and/or p-anisidine (0.01 mol), were combined. After that, the reaction contents were heated for 12 h under reflux. The formation of benzyl mercaptan and/or methyl mercaptan was observed by soaking filter paper in sodium nitroprusside, which developed a pink color. The resultant precipitate was filtered and rinsed with ethyl alcohol, followed by recrystallization from an appropriate solvent to yield compounds 11, 12, and 13.

##### 2-(1-(4-((3-methylpiperidin-1-yl)sulfonyl)phenyl)ethylidene)-N-(p-tolyl) hydrazine-1-carbothioamide, 11

Yellowish white solid from 1,4-dioxane (yield 80%), Rf = 0.57 (DCM/MeOH, 9:1 v/v), HPLC purity (area %): 98.53%, m. p. 210–212 °C; IR (KBr, υ_max_ cm⁻¹): 3309, 3290 (2NH), 3032 (C-H, aromatic), 2946, 2852 (C-H, aliphatic), 1613 (C = N), and 1336,1165 (SO_2_). ^1^H NMR (400 MHz, DMSO) δ_H_ = 10.70 (s, 1H, NH, exchangeable by D_2_O), 10.08 (s, 1H, NH, exchangeable by D_2_O), 8.26 (d, *J* = 8.4 Hz, 2 H, AB-Ar-H), 7.72 (d, *J* = 8.5 Hz, 2 H, AB-Ar-H), 7.42 (d, *J* = 8.2 Hz, 2 H, AB-Ar-H), 7.19 (d, *J* = 8.0 Hz, 2 H, AB-Ar-H), 3.49 (t, 2 H, CH_2_, piperidinyl_(2)_), 2.42 (s, 3 H, CH_3_-C = N), 2.32 (s, 3 H, CH_3_ (p-position)), 2.21 (d, *J* = 2.6 Hz, 2 H, CH_2_, piperidinyl_(6)_), 1.90 (t, *J* = 10.7 Hz, 1H, CH, piperidinyl_(3)_), 1.71–1.56 (m, 2 H, CH_2_, piperidinyl_(4)_), 1.54–1.41 (m, 2 H, CH_2_, piperidinyl_(5)_), 0.83 (d, *J* = 6.5 Hz, 3 H, CH_3_-piperidinyl). ^13^C NMR (101 MHz, DMSO) δ_C_ = 177.83 (thiocarbonyl, C = S), 147.21 (-C = N-), 142.16, 137.00, 136.18, 135.20, 129.05, 127.74, 127.68, 126.42 (aromatic carbons), 53.14 (piperidinyl moiety-C_2_), 46.57 (piperidinyl moiety-C_6_), 31.76 (piperidinyl moiety-C_4_), 30.65 (piperidinyl moiety-C_3_), 24.60 (piperidinyl moiety-C_5_), 21.09 (CH_3_), 19.15 (**C**H_3_-C = N), 14.78 (carbon of CH_3_-piperidinyl moiety). MS (*m*/*z*): 444 [%]: [M^+^, (13.31%)], base peak; 169 (100%), Anal. Calcd for C_22_H_28_N_4_O_2_S_2_ (444.80): C, 59.43; H, 6.35; N, 12.60; Found: C, 59.33; H, 6.28; N, 12.49%.

##### 2-(1-(4-((3-methylpiperidin-1-yl)sulfonyl)phenyl)ethylidene)-N-(m-tolyl) hydrazine-1-carbothioamide, 12

Yellowish white solid from 1,4-dioxane (yield 80%), Rf = 0.6 (DCM/MeOH, 9:1 v/v), HPLC purity (area %): 99.89%, m.p. 238–240 °C; IR (KBr, υ_max_ /cm^− 1^): 3309, 3224 (2NH), 3059(C-H aromatic), 2920, 2860 (C-H aliphatic), 1598 (C = N) and 1338,1157 (SO_2_); ^1^H NMR (400 MHz, DMSO) δ_H_ = 10.73 (s, 1H, NH), 10.09 (s, 1H, NH), 8.26 (d, *J* = 8.5 Hz, 2 H, AB-Ar-H), 7.72 (d, *J* = 8.6 Hz, 2 H, AB-Ar-H), 7.45–7.35 (m, 2 H, phenyl group), 7.27 (t, *J* = 7.7 Hz, 1H, Phenyl group), 7.05 (d, *J* = 7.5 Hz, 1H, Phenyl group), 3.52 (t, *J* = 10.5 Hz, 2 H, CH_2_, piperidinyl_(2)_), 2.42 (s, 3 H, CH_3_-C = N-), 2.34 (s, 3 H, CH_3_ (m-position), 2.27–2.16 (m, 2 H, CH_2_, piperidinyl_(6)_), 1.93 (dt, *J* = 18.5, 10.7 Hz, 1H, CH, piperidinyl_(3)_), 1.74–1.57 (m, 2 H, CH_2_, piperidinyl_(4)_), 1.54–1.39 (m, 2 H, CH_2_, piperidinyl_(5)_), 0.84 (d, *J* = 6.3 Hz, 3 H, CH_3_-piperidinyl moiety). ^13^C NMR (101 MHz, DMSO) δ_C_ = 177.69 (thiocarbonyl, C = S), 147.29 (-C-C = N-), 142.14, 139.43, 137.87, 136.19, 128.40, 128.07, 127.69, 126.65, 123.62 (aromatic carbons), 53.14 (piperidinyl moiety-C_2_), 46.57 (piperidinyl moiety-C_6_), 31.75 (piperidinyl moiety-C_4_), 30.65 (piperidinyl moiety-C_3_), 24.60 (piperidinyl moiety-C_5_), 21.42 (CH_3 (m−position)_), 19.16 (**C**H_3_-C = N), 14.80 (carbon of CH_3_-piperidinyl moiety). MS (*m*/*z*): 444 [%]: [M^+^, (19.07%)], base peak; 313 (100%), Anal. Calcd for C_22_H_28_N_4_O_2_S_2_ (444.17): C, 59.43; H, 6.35; N, 12.60; Found: C, 59.32; H, 6.27; N, 12.47%.

##### N-(4-methoxyphenyl)-2-(1-(4-((3-methylpiperidin-1-yl)sulfonyl)phenyl)-ethylidene) hydrazine-1-carbothioamide, 13

White solid from 1,4-dioxane (yield 85%), Rf = 0.55 (DCM/MeOH, 9:1 v/v), HPLC purity (area %): 98.90%, m.p. 226–228 °C; IR (KBr, υ_max_ cm⁻¹): 3293, 3209 (2NH), 3064 (C-H aromatic), 2929, 2851 (C-H aliphatic), 1612 (C = N), and 1341,1157 cm^− 1^ (SO_2_). ^1^H NMR (400 MHz, DMSO) δ_H_ = 10.67 (s, 1H, NH, exchangeable by D_2_O), 10.05 (s, 1H, NH, exchangeable by D_2_O), 8.26 (d, *J* = 8.4 Hz, 2 H, AB-Ar-H), 7.72 (d, *J* = 8.3 Hz, 2 H, AB-Ar-H), 7.39 (d, *J* = 8.9 Hz, 2 H, AB-Ar-H), 6.95 (d, *J* = 8.9 Hz, 2 H, AB-Ar-H), 3.78 (s, 3 H, OCH_3_), 3.51 (d, 2 H, CH_2_, piperidinyl_(2)_), 2.42 (s, 3 H, CH_3_-C = N), 2.21 (d, 2 H, CH_2_, piperidinyl_(6)_), 1.90 (t, *J* = 10.7 Hz, 1H, CH, piperidinyl_(3_), 1.67–1.61 (m, 2 H, CH_2_, piperidinyl_(4)_), 1.58–1.28 (m, 2 H, CH_2_, piperidinyl_(5)_), 0.83 (d, *J* = 6.5 Hz, 3 H, CH_3_-piperidinyl moiety). ^13^C NMR (101 MHz, DMSO) δ_C_ = 178.15 (thiocarbonyl, C = S), 157.58 (aromatic carbon), 147.08 (-C-C = N-), 142.18, 136.14, 132.46, 128.20, 128.05, 127.66, 113.79 (aromatic carbon), 55.73 (OCH_3_), 53.14 (piperidinyl moiety-C_2_), 46.57 (piperidinyl moiety-C_6_), 31.75 (piperidinyl moiety-C_4_), 30.65 (piperidinyl moiety-C_3_), 24.59 (piperidinyl moiety-C_5_), 19.15 (CH_3_), 14.72 (carbon of CH_3_-piperidinyl moiety). MS (*m*/*z*): 460 [%]: [M^+^, (37.30%)], base peak; 410 (100%), Anal. Calcd for C_22_H_28_N_4_O_3_S_2_ (460.61): C, 57.37; H, 6.13; N, 12.16; Found: C, 57.26; H, 6.05; N, 12.02%.

#### General protocol for the synthesis of derivatives 14, 15, and 16

After dissolving crucial intermediates 11, 12, and/or 13 (0.01 mol) and monochloroacetic acid (MCA) (0.01 mol) in 20 milliliters of anhydrous ethyl alcohol, 0.01 mol of anhydrous sodium acetate (catalyst) was added. After stirring the mixture at 100 °C for seven hours, it was filtered. The crude product was cleaned using ethyl alcohol and water. Under a vacuum, the solid product was dried. After that, the crude product was refined by recrystallization with the right solvent to produce 14–16, respectively.

##### (Z)-2-(((E)-1-(4-(((S)-3-methylpiperidin-1-yl)sulfonyl)phenyl)ethylidene) hydraziney-lidene)-3-(p-tolyl)thiazolidin-4-one, 14

White solid from 1,4-dioxane (yield 75%), Rf = 0.7 (DCM/MeOH, 9:1 v/v), HPLC purity (area %): 99.65%, m.p. 255–257 °C; IR (KBr, υ_max_ cm^−1^): 3039 (C-H aromatic), 2927, 2873 (C-H aliphatic), 1728 (C = O), 1608 (C = N), and 1373,1157 cm^− 1^ (SO_2_). ^1^H NMR (400 MHz, DMSO) δ_H_ = 8.05 (d, *J* = 8.1 Hz, 2 H, AB-Ar-H), 7.79 (d, *J* = 8.1 Hz, 2 H, AB-Ar-H), 7.34 (d, *J* = 8.2 Hz, 2 H, AB-Ar-H), 7.30 (d, *J* = 8.1 Hz, 2 H, AB-Ar-H), 4.11 (s, 2 H, CH_2_-thiazolidinone ring), 3.51 (t, *J* = 11.4 Hz, 2 H, CH_2_, piperidinyl_(2)_), 2.39 (s, 3 H, CH_3_-C = N-), 2.22 (s, 3 H, CH_3_ (p-position)), 2.06 (d, 2 H, CH_2_, piperidinyl_(6)_), 1.93 (t, *J* = 10.7 Hz, 1H, CH, piperidinyl_(3)_), 1.74–1.56 (m, 2 H, CH_2_, piperidinyl_(4)_), 1.55–1.40 (m, 2 H, CH_2_, piperidinyl_(5)_), 0.84 (d, *J* = 6.3 Hz, 3 H, CH_3_-piperidinyl moiety). ^13^C NMR (101 MHz, DMSO) δ_C_ = 172.42 (thiazolidinone ring-C_4_), 165.44 (-C-C = N-), 161.04 (S-C = N-), 141.97, 138.55, 136.94, 132.93, 129.92, 128.19, 128.04, 127.65 (aromatic carbon), 53.08 (piperidinyl moiety-C_2_), 46.56 (piperidinyl moiety-C_6_), 32.82 (thiazolidinone ring-C_3_), 31.69 (piperidinyl moiety-C_4_), 30.70 (piperidinyl moiety-C_3)_, 24.61 (piperidinyl moiety-C_5_), 21.25 (CH_3 (p−position)_), 19.15 (CH_3_-C = N-), 15.18 (carbon of CH_3_-piperidinyl moiety). MS (*m*/*z*): 484 [%]: [M^+^, (50.87%)], base peak; 340 (100%), Anal. Calcd for C_24_H_28_N_4_O_3_S_2_ (484.16): C, 59.48; H, 5.82; N, 11.56; Found: C, 59.36; H, 5.73; N, 11.42%.

##### (Z)-2-(((E)-1-(4-(((S)-3-methylpiperidin-1-yl)sulfonyl)phenyl)ethylidene) hydraziney-lidene)-3-(m-tolyl)thiazolidin-4-one, 15

Yellowish white solid from 1,4-dioxane (yield 64%), Rf = 0.72 (DCM/MeOH, 9:1 v/v), HPLC purity (area %): 98.65%, m.p. 205–207 °C; IR (KBr, υ_max_ cm⁻¹): 3059 (C-H aromatic), 2924, 2873 (C-H aliphatic), 1728 (C = O), 1612 (C = N), and 1338, 1157 cm^− 1^ (SO_2_). ^1^H NMR (400 MHz, DMSO) δ_H_ = 8.04 (d, *J* = 8.5 Hz, 2 H, AB-Ar-H), 7.79 (d, *J* = 8.5 Hz, 2 H, AB-Ar-H), 7.42 (t, *J* = 7.7 Hz, 1H, phenyl group), 7.28 (d, *J* = 7.7 Hz, 1H, phenyl group), 7.23 (d, *J* = 3.9 Hz, 1H, phenyl group), 7.21 (s, 1H, phenyl group), 4.12 (s, 2 H, CH_2_-thiazolidinone ring), 3.51 (t, *J* = 11.2 Hz, 2 H, CH_2_, piperidinyl_(2)_), 2.38 (s, 3 H, CH_3_-C = N-), 2.22 (s, 5 H, CH_3_ (m-position), and 2 H, CH_2_, piperidinyl_(6)_), 1.94 (td, *J* = 11.0, 4.7 Hz, 1H, CH, piperidinyl_(3)_), 1.74–1.56 (m, 2 H, CH_2_, piperidinyl_(4)_), 1.56–1.40 (m, 2 H, CH_2_, piperidinyl_(5)_), 0.84 (d, *J* = 6.1 Hz, 3 H, CH_3_-piperidinyl moiety). ^13^C NMR (101 MHz, DMSO) δ_C_ = 172.36 (thiazolidinone ring-C_4_), 165.34 (-C-C = N-), 161.08 (-S-C = N-), 141.97, 138.91, 136.96, 135.46, 129.71, 129.21, 128.83, 128.03, 127.65, 125.52 (aromatic carbon), 53.07 (piperidinyl moiety-C_2_), 46.56 (piperidinyl moiety-C_6_), 32.86 (thiazolidinone ring-C_3_), 31.69 (piperidinyl moiety-C_4_), 30.70 (piperidinyl moiety-C_3_), 24.62 (piperidinyl moiety-C_5_), 21.27 (CH_3 (m−position)_), 19.15 (CH_3_-C = N-), 15.17 (carbon of CH_3_-piperidinyl moiety). MS (*m*/*z*): 484 [%]: [M^+^, (38.98%)], base peak; 241 (100%), Anal. Calcd for C_24_H_28_N_4_O_3_S_2_ (484.16): C, 59.48; H, 5.82; N, 11.56; Found: C, 59.37; H, 5.74; N, 11.43%.

##### 3-(4-methoxyphenyl)-2-(((E)-1-(4-((3-methylpiperidin-1-yl)sulfonyl)phenyl) ethylidene)hydrazono)thiazolidin-4-one, 16

Pale-yellow solid from 1,4-dioxane (yield 70%), Rf = 0.67 (DCM/MeOH, 9:1 v/v), HPLC purity (area %): 99.56%, m. p. IR (KBr, υmax cm^−1^): 3075 (C-H aromatic), 2928, 2872 (C-H aliphatic), 1729 (C = O), 1609 (C = N), and 1337,1156 cm^−1^ (SO_2_). ^1^H NMR (400 MHz, DMSO) δ_H_ = 8.04 (d, *J* = 8.7 Hz, 2 H, AB-Ar-H), 7.79 (d, *J* = 8.7 Hz, 2 H, AB-Ar-H), 7.34 (d, *J* = 8.9 Hz, 2 H, AB-Ar-H), 7.07 (d, *J* = 9.0 Hz, 2 H, AB-Ar-H), 4.10 (s, 2 H, CH_2_-thiazolidinone ring), 3.82 (s, 3 H, OCH_3_), 3.51 (t, *J* = 10.5 Hz, 2 H, CH_2_, piperidinyl_(2)_), 2.23 (s, 3 H, CH_3_-C = N-), 2.08 (d, *J* = 10.1 Hz, 2 H, CH_2_, piperidinyl_(6)_), 1.93 (t, *J* = 10.8 Hz, 1H, CH, piperidinyl_(3)_), 1.72–1.57 (m, 2 H, CH_2_, piperidinyl_(4)_), 1.54–1.40 (m, 2 H, CH_2_, piperidinyl_(5)_), 0.84 (d, *J* = 6.4 Hz, 3 H, CH_3_-piperidinyl moiety). ^13^C NMR (101 MHz, DMSO) δ_C_ = 172.54 (thiazolidinone ring-C_4_), 165.61 (-C-C = N-), 160.98 (S-C = N-), 159.49 (-C-OCH_3_), 144.18, 141.98, 136.91, 129.60, 128.01, 127.64, 114.62 (aromatic carbon), 55.85 (OCH_3_), 53.07 (piperidinyl moiety-C_2_), 46.56 (piperidinyl moiety-C_6_), 32.76 (thiazolidinone ring-C_3_), 31.68 (piperidinyl moiety-C_4_), 30.68 (piperidinyl moiety-C_3_), 24.59 (piperidinyl moiety-C_5_), 19.14 (CH_3_-C = N-), 15.15 (carbon of CH_3_-piperidinyl moiety). MS (*m*/*z*): 500 [%]: [M^+^, (11.96%)], base peak; 361 (100%), Anal. Calcd for C_24_H_28_N_4_O_4_S_2_ (500.16): C, 57.58; H, 5.64; N, 11.19; Found: C, 57.46; H, 5.55; N, 11.07%.

## Biological investigation

### In vitro cytotoxicity

The in vitro cytotoxic activities of the synthesized molecules were tested against the three selected human tumor cell lines using the MTT assay protocol as described elsewhere. Briefly, cells were seeded in 96-well plates at an adequate density. Then, the plates were incubated for 24 h at optimal conditions. After the incubation time, the medium was replaced with 0.1 ml of fresh medium containing serial dilutions of the test compounds and incubated for 48 h. Then, 10 µl of MTT solution (5 µg/ml) was included to each well, and then the plates were incubated for an additional 4 h. Following, the formed MTT-formazan crystals were dissolved in 100 µl of DMSO. The absorbance of each well was measured at 490 nm using an automatic ELISA reader system (TECAN, CHE). The IC_50_ values were calculated using the nonlinear regression fitting models using GraphPad Prism. The data showed the mean of three independent replicates in triplicate and were expressed as means ± SD. Vincristin, the commercially available drug, was used as a positive control.

### In vitro enzymatic inhibition

The ability of compound 16 to inhibit the selected enzymes was evaluated using human ELISA kits for each enzyme. The appropriate antibody was mounted on a 96-well plate, followed by the addition of compound **16** or the standard solution. The plates were incubated at room temperature for 2.5 h, followed by washing (3X). Then, 100 µL of the prepared biotin antibody was added, and then the plates were incubated for an additional one hour at room temperature. The plates were washed again, and 100 µL of streptavidin solution was added. The plates were then incubated for 45 min. Following, the plates were washed with 100 µL of TMB, substrate reagent was added, and the plates were incubated for an additional 30 min. Finally, 50 µL of the stop solution was added, and the absorbance values were immediately measured at 450 nm.

### Cell cycle analysis

MCF-7 cells were exposed to compound **16** at its IC_50_ value for 72 h. The cells were then harvested, washed with PBS, and fixed. The fixed cells were stained with the Cycle TESTTM PLUS DNA Reagent Kit (BD Biosciences, San Jose, CA) according to the manufacturer’s instructions. Cell-cycle distribution was evaluated using a flow cytometer. DMSO was chosen as the negative control, and doxorubicin was used as a positive control.

### Apoptosis analysis

MCF-7 cells were propagated in a 96-well plate and then treated with compound **16** at its IC_50_ value for 72 h. Following, the cells were collected by centrifugation after washing with PBS. Then the collected cells were stained by Annexin V-FITC and propidium iodide (PI) in the binding buffer for 20 min using the apoptosis detection kit (BD Biosciences, San Jose, CA). Annexin V-FITC and PI binding were analyzed and detected using a flow cytometer, and the frequencies in all quadrants were analyzed using FlowJo software. DMSO was chosen as the negative control, and doxorubicin was used as a positive control.

### Molecular docking study

The crystal structure of VEGFR-2 (PDB ID: 2OH4, resolution: 2.05 Å) was retrieved from the Protein Data Bank (https://www.rcsb.org). Protein preparation was carried out following standard protocols, including protonation, correction of structural issues, and energy minimization using the Hamiltonian AM1 method. Structural optimization was performed using the MMFF94x force field. The 3D structures of the lead compound (**A**) and the newly synthesized compound (**16**) were constructed and subjected to conformational optimization prior to docking. Molecular docking was performed on the MOE 2019 platform. To validate the docking protocol, the native co-crystallized ligand was re-docked into its original binding site, and the resulting pose was compared to the crystallographic orientation by calculating the root mean square deviation (RMSD) of heavy atoms. A successful validation was confirmed by an RMSD value within the acceptable threshold (< 2.0 Å), indicating reliable docking conditions. Using the validated protocol, docking of the test compounds was performed. For each ligand, 20 docking poses were generated using the Alpha Triangle placement method, with the ASE scoring function and rigid receptor refinement. The top-ranked pose (based on favorable binding interactions and docking score) was selected for further analysis. Interaction analysis was conducted using Discovery Studio Visualizer v4.0. Comparative analysis of docking poses was performed by evaluating the binding modes and docking scores of the test compounds relative to the re-docked reference ligand.

## Computational methodology

All calculations were performed using Gaussian 09 software. The geometry of compound **16** was optimized at the M05-2X-D3/6-31G** level, which includes dispersion corrections and is well-suited for describing non-covalent interactions in biological systems. Frequency calculations confirmed the absence of imaginary frequencies, ensuring stable minima. The energies of the highest occupied molecular orbital (HOMO) and lowest unoccupied molecular orbital (LUMO) were used to derive the following reactivity parameters: Ionization Potential (IP) ≈ –EHOMO, Electron Affinity (EA) = –ELUMO, Energy Gap (ΔƐ) = ELUMO – EHOMO, Chemical Hardness (η) = (IP – EA)/2, Softness (σ) = 1/η, Electronegativity (χ) = (IP + EA)/2, Electrophilicity Index (ω) = χ² / 2η.

## Supplementary Information

Below is the link to the electronic supplementary material.

Supplementary Material 1

## Data Availability

The datasets utilized and analyzed in this study can be obtained from the corresponding author upon request.
