# Supplementary material for: Structure-guided optimization of N-sulfonylpiperidines toward potent multi-target anticancer agents
Source: Sci Rep. 2026 Apr 13;16:12230. doi: 10.1038/s41598-026-44109-z (PMC13076777; doi:10.1038/s41598-026-44109-z)

Structure-Guided Optimization of N-Sulfonylpiperidines toward Potent Multi-target Anticancer Agents

Al Ghazali S. Al Jazairi [a], Walid E. Elgammal [a **], Mahmoud Basseem I. Mohamed [a], Mohamed A. Seleem [b], Mahmoud S. Bashandy [a]

[a] Chemistry Department, Faculty of Science (Boys), Al-Azhar University, Nasr City, 11884, Cairo, Egypt.

[b] Department of Pharmaceutical Organic Chemistry, Faculty of Pharmacy, Al-Azhar University, Cairo 11884, Egypt.

Corresponding author:

Walid E. Elgammal
Chemistry Department, Faculty of Science, Al-Azhar University, Nasr City, Cairo, Egypt, Tel: +201025267111, E-mail: walidebaied.sci85@azhar.edu.eg, ORCID: 0000-0002-5982-5635, Al-Azhar University, El-Nasr Road, Nasr City, 11884, Cairo, Egypt

Content
1. Chemistry
2. Spectral data
3. Biological activity (dose-response curve)

1. Chemistry:

Reagents and solvents for the synthesis were procured from a range of reputable suppliers, including Sigma-Aldrich, Alpha Chem, Fluka, and Loba. These reagents were utilized without any further purification. Thin-layer chromatography (TLC) was executed on silica gel plates utilizing a DCM and MeOH mixture in a 95:5 ratio as the elution system. The progression of the reaction and the assessment of product purity were monitored using a UV indicator at a wavelength of 254 nm. For nuclear magnetic resonance (NMR) analysis, both 1-proton and 13-carbon spectra were collected using a JNM-ECA 500 II instrument manufactured by JEOL-Japan, employing deuterated dimethyl sulfoxide at frequencies of 300 and 400 MHz for ^{1}H NMR and 75, 101MHz for ^{13}C NMR. Chemical shifts (delta δ) are expressed in parts per million (ppm), while signal multiplicities are designated as br s (broad singlet), s (singlet), d (doublet), dd (doublet of doublets), t (triplet), q (quartet), and m (multiplet). Coupling constants (J) are reported in hertz (Hz). The ^{1}H and ^{13}C spectra were referenced to the residual solvent signals: DMSO-d_6 (2.50 or 39.52 ppm), $CDCl_3$ (7.26 or 77.16 ppm). The spectra were referenced against the internal standard

organosilicon compound, such as tetramethylsilane with the formula $Si(CH_3)_4$ and abbreviated as (TMS) peak at (δ=0.00 ppm), with all ^1H NMR spectra showing a water peak at (δ=3.33 ppm). NMR spectra processing was conducted using MestreNova version 14.3.3. Melting points (°C, uncorrected) were determined using open capillaries on a Gallen Kemp melting point apparatus sourced from Sanyo Gallen Kemp in Southborough, UK. We measured mass spectra using a Thermo Scientific GCMS model (Isq Lt), together with Thermo X-Calibur software from Shimadzu Corporation, Kyoto, Japan, at the Regional Center for Mycology and Biotechnology (RCMB), Al-Azhar University, Nasr City, Cairo, Egypt. An elemental analysis (C-H-N) was conducted at Al-Azhar University, Cairo, Egypt, with results accurate to 0.4%.

2. Spectral data

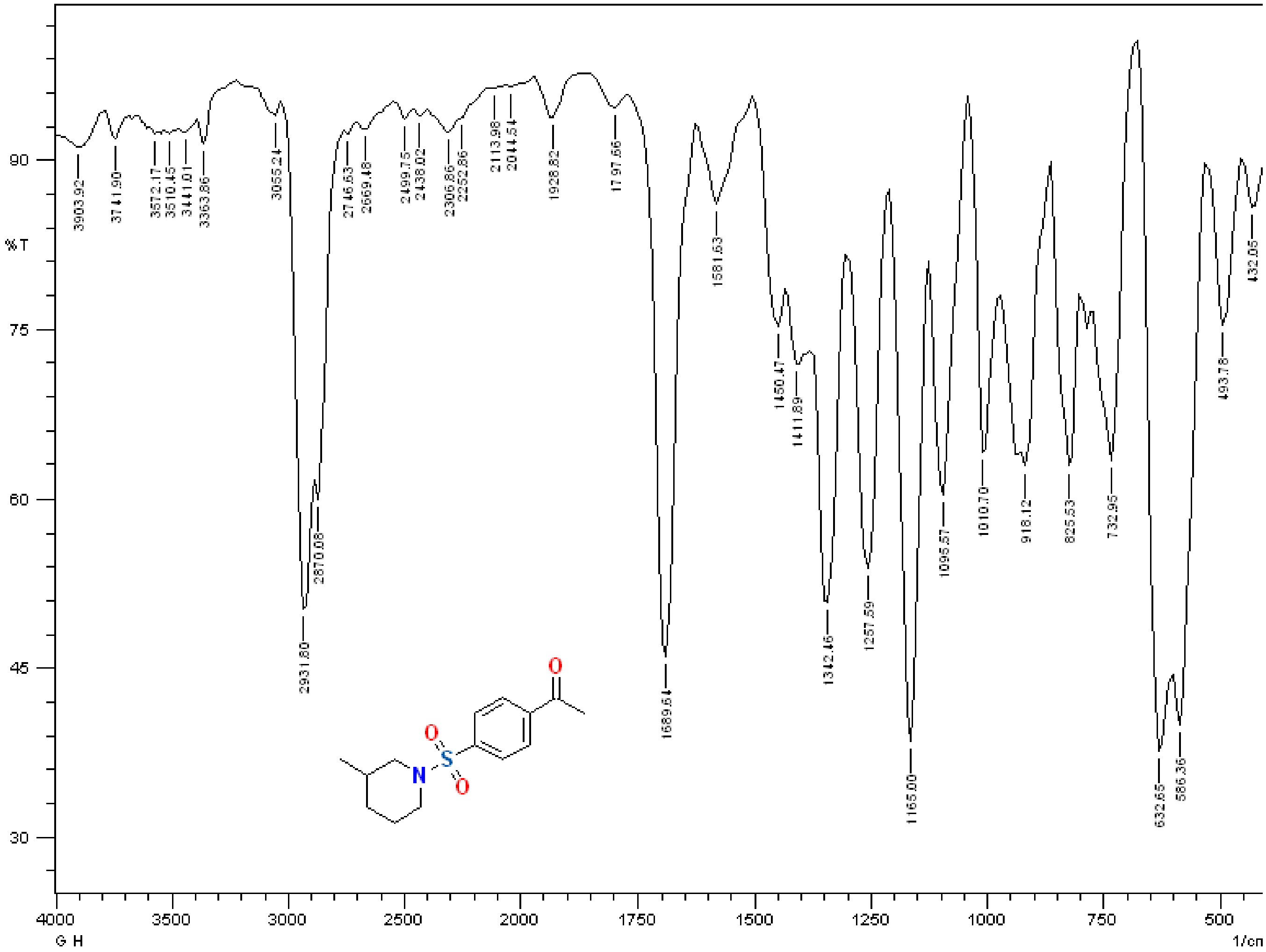

*Fig S1: FT-IR (*KBr, ν cm^{-1}) *of starting material 3.*

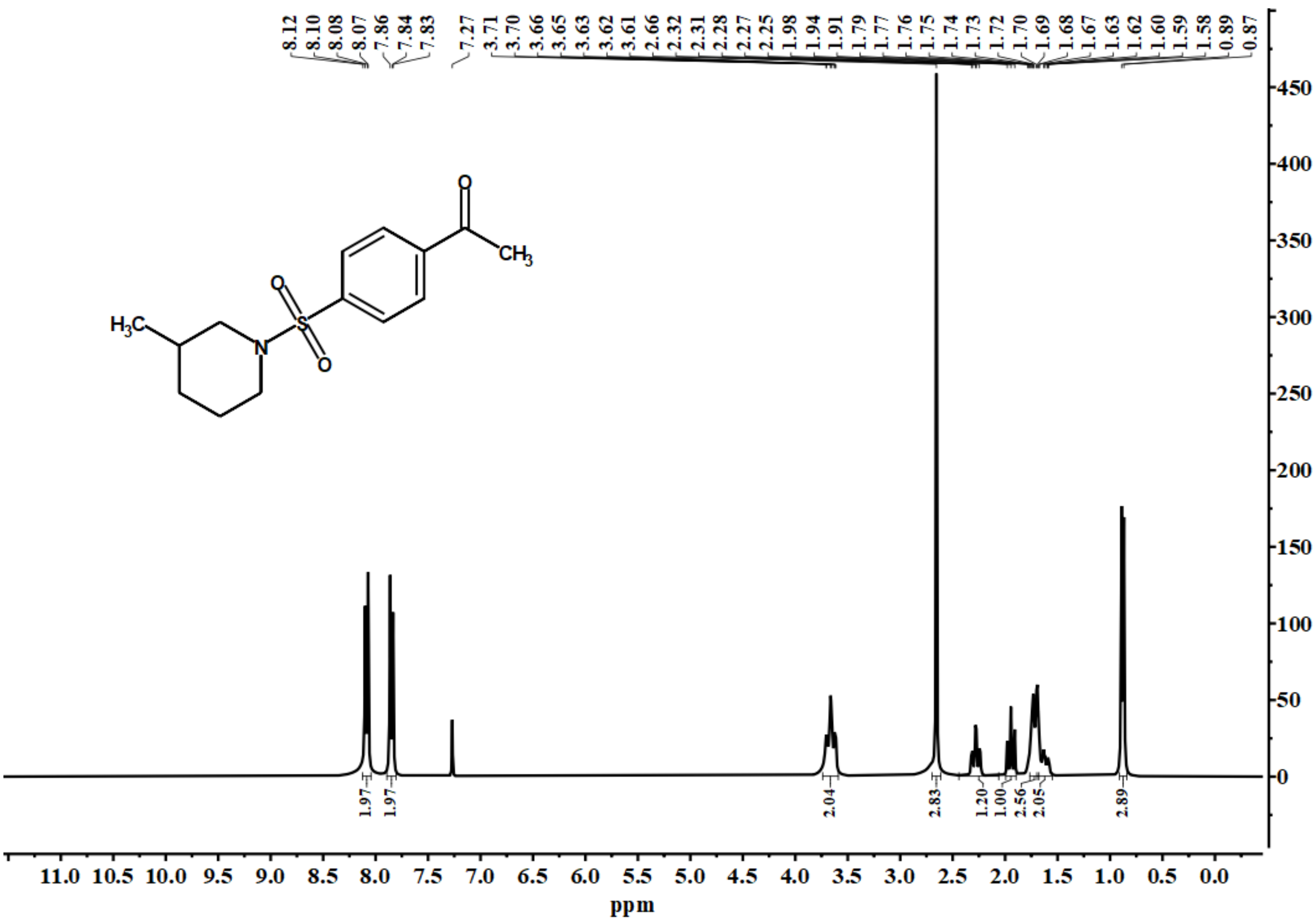

*Fig S2: ^{1}H NMR (*CDCl$_3$) *of starting material 3*

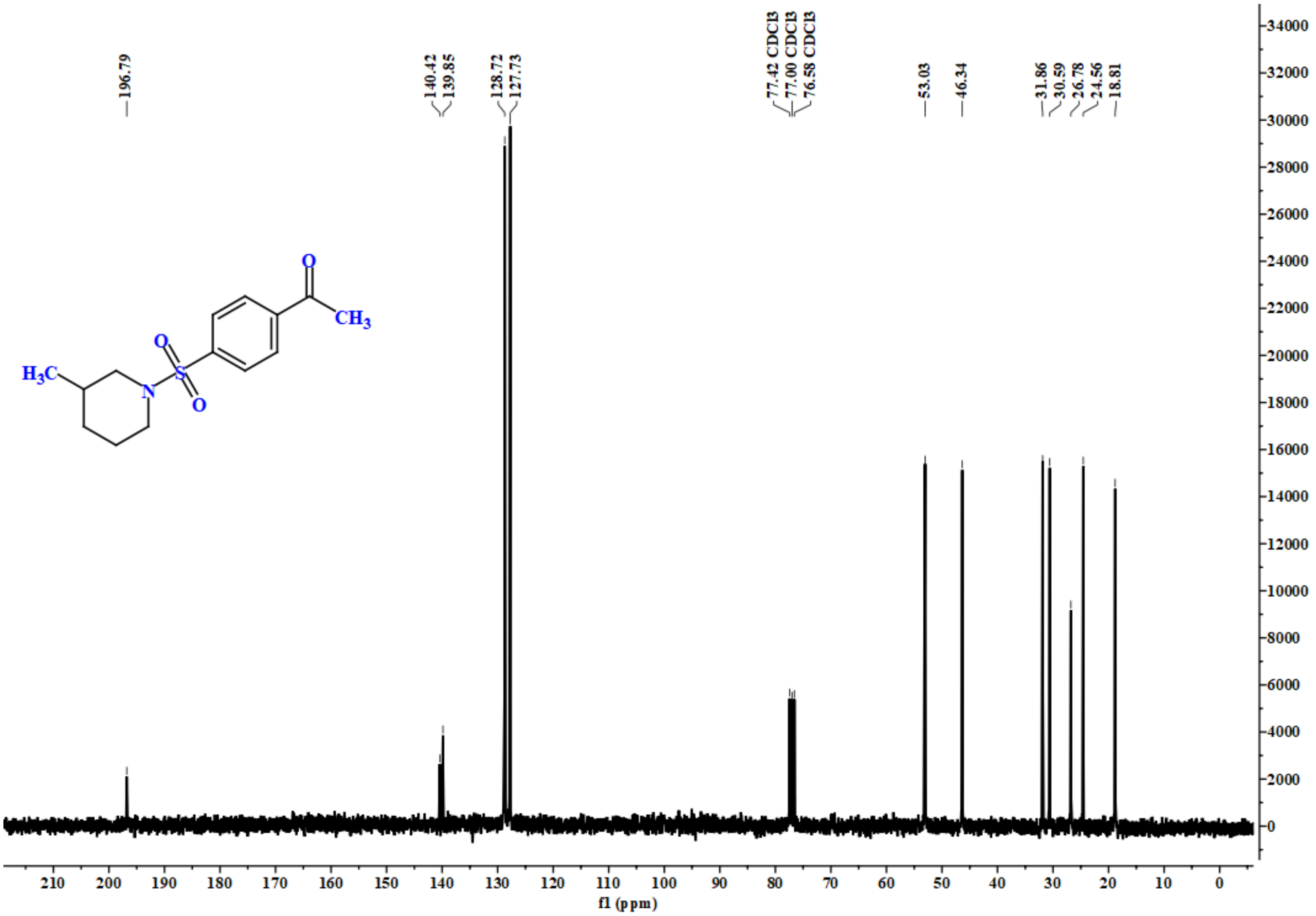

Fig S3: ^{13}C NMR ($CDCl_3$) of *starting material* 3

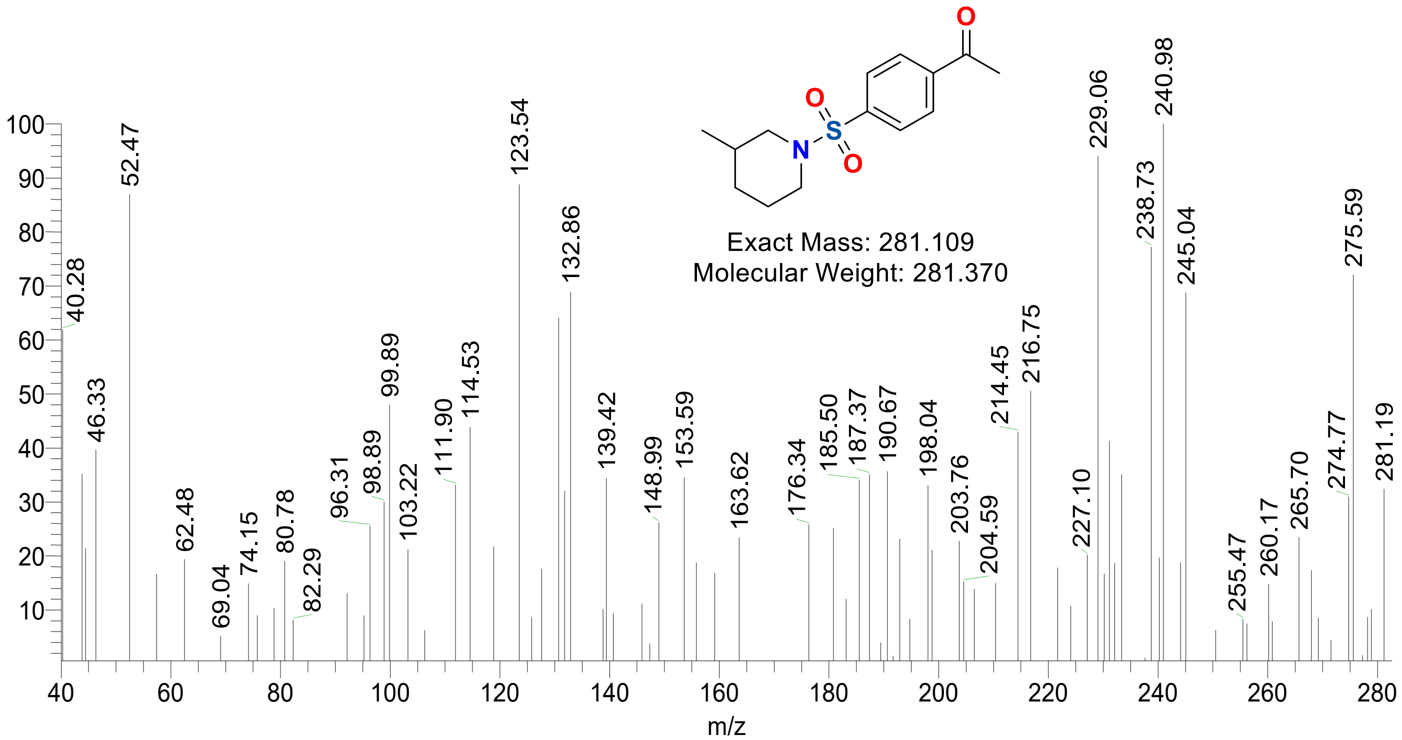

Fig S4: Mass spectrum of *starting material* 3

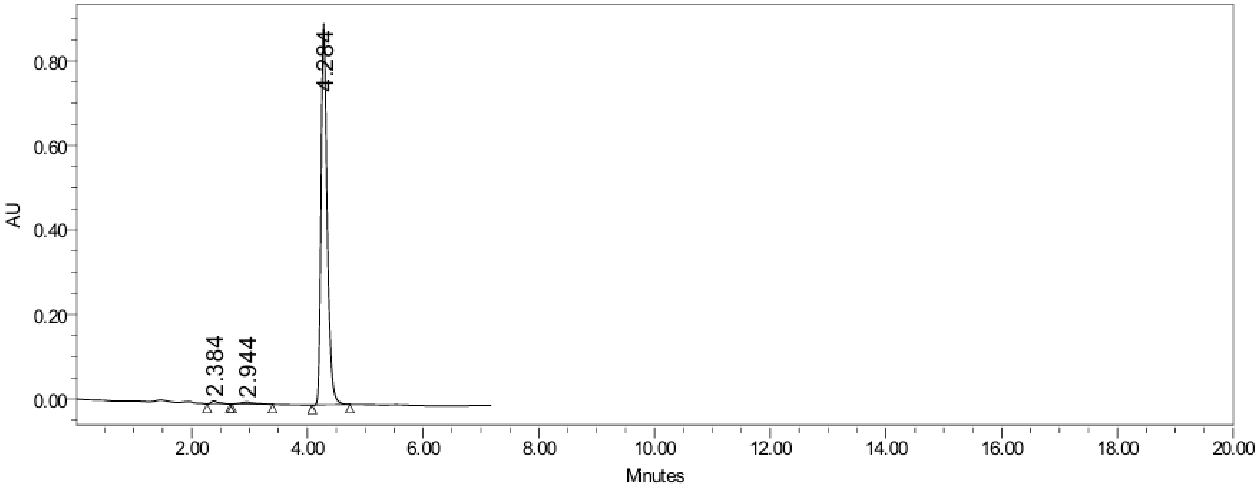

	RT	Area	% Area	USP Plate Count	USP Tailing	K Prime
1	2.384	64003	0.95	1756.44	1.70	1.27
2	2.944	81333	1.21	745.93	1.42	1.80
3	4.284	6577921	97.84	8346.18	1.35	3.08

Fig S5: HPLC chromatogram of starting material 3

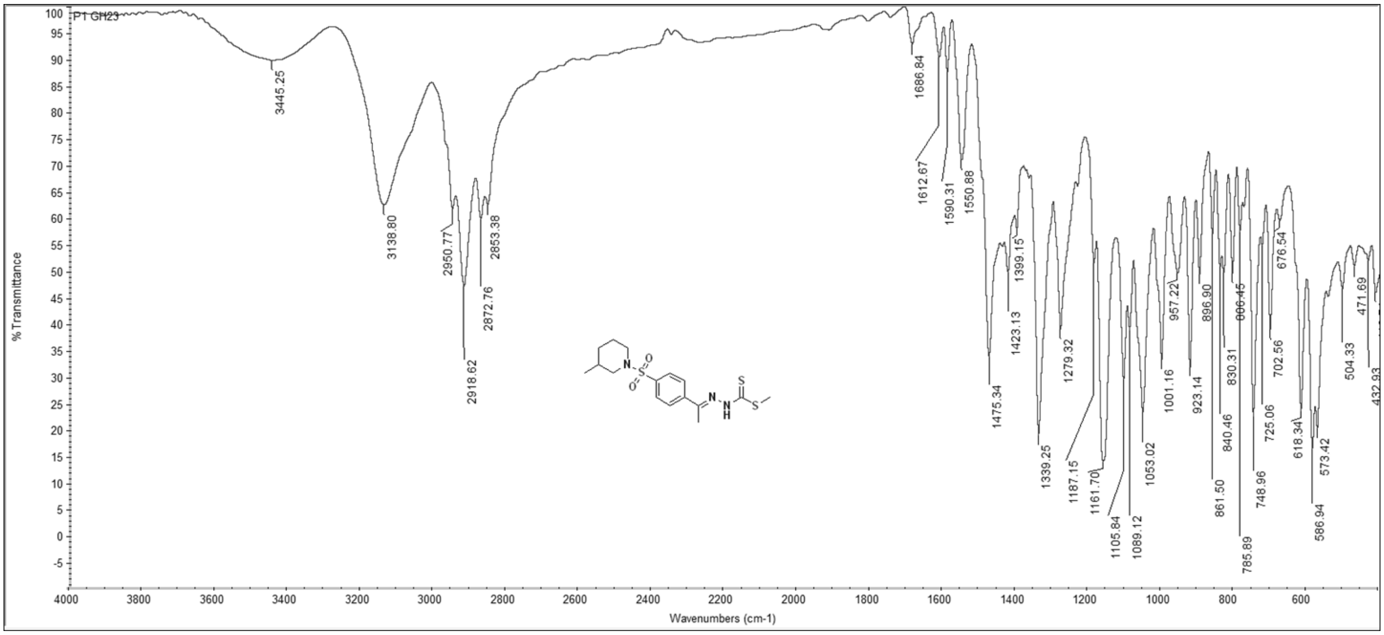

*Fig S6: FT-IR (*KBr, ν cm^{-1}) *of starting material 9.*

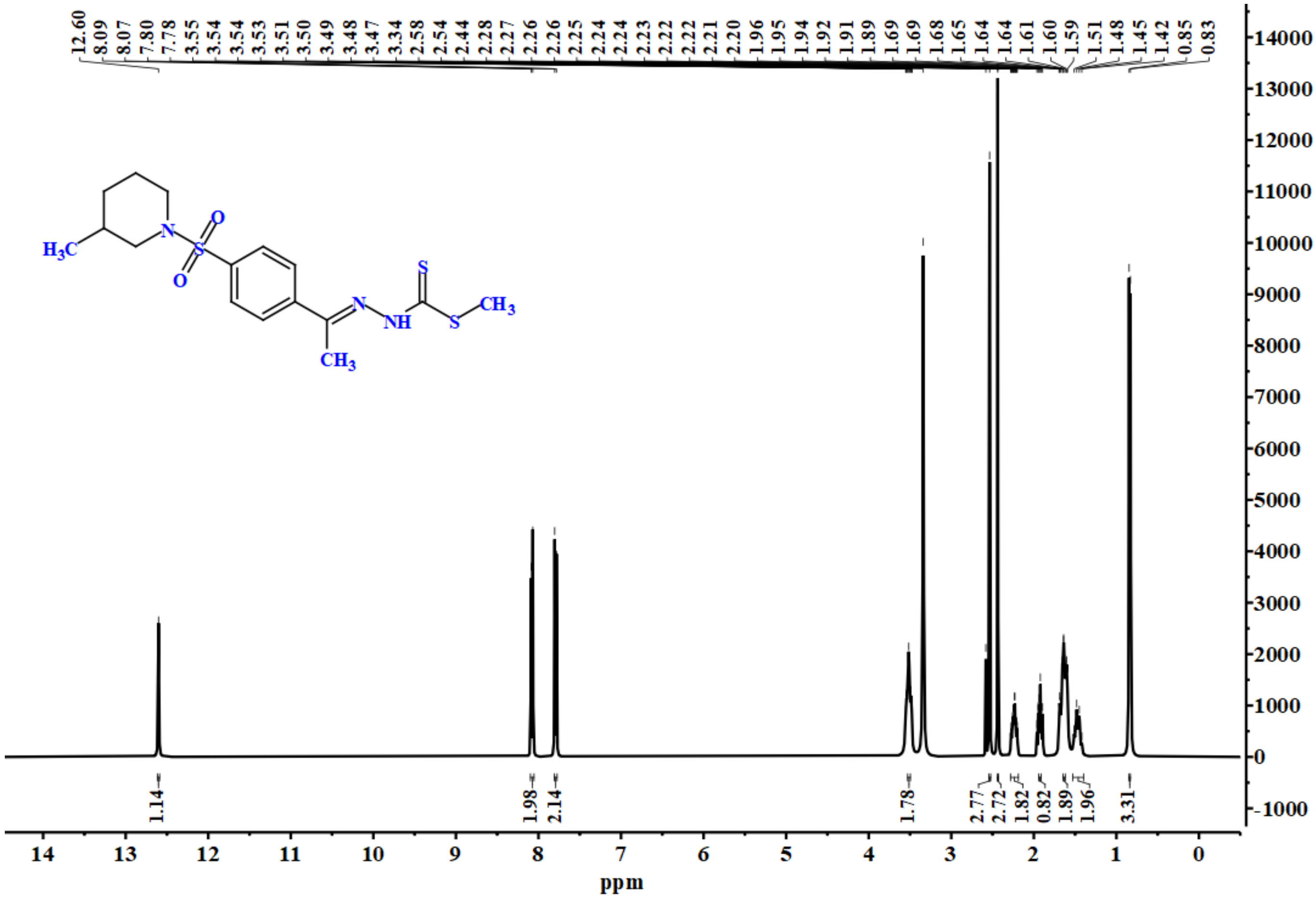

*Fig S7: ^{1}H NMR (*DMSO-d$_6$) *of starting material 9*

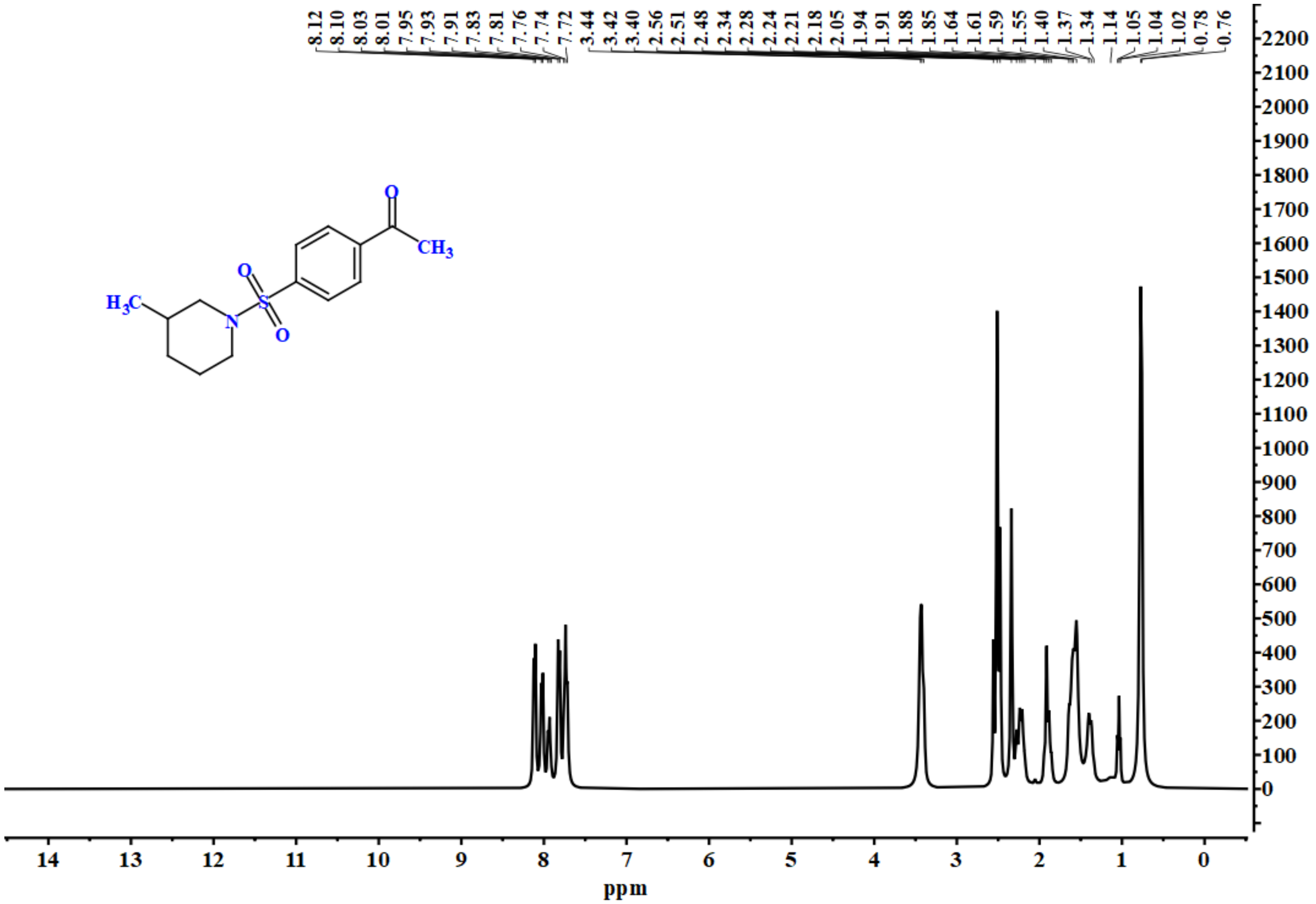

Fig S8: ^{1}H NMR / D_2O (**DMSO-d_6) ***of starting material   9

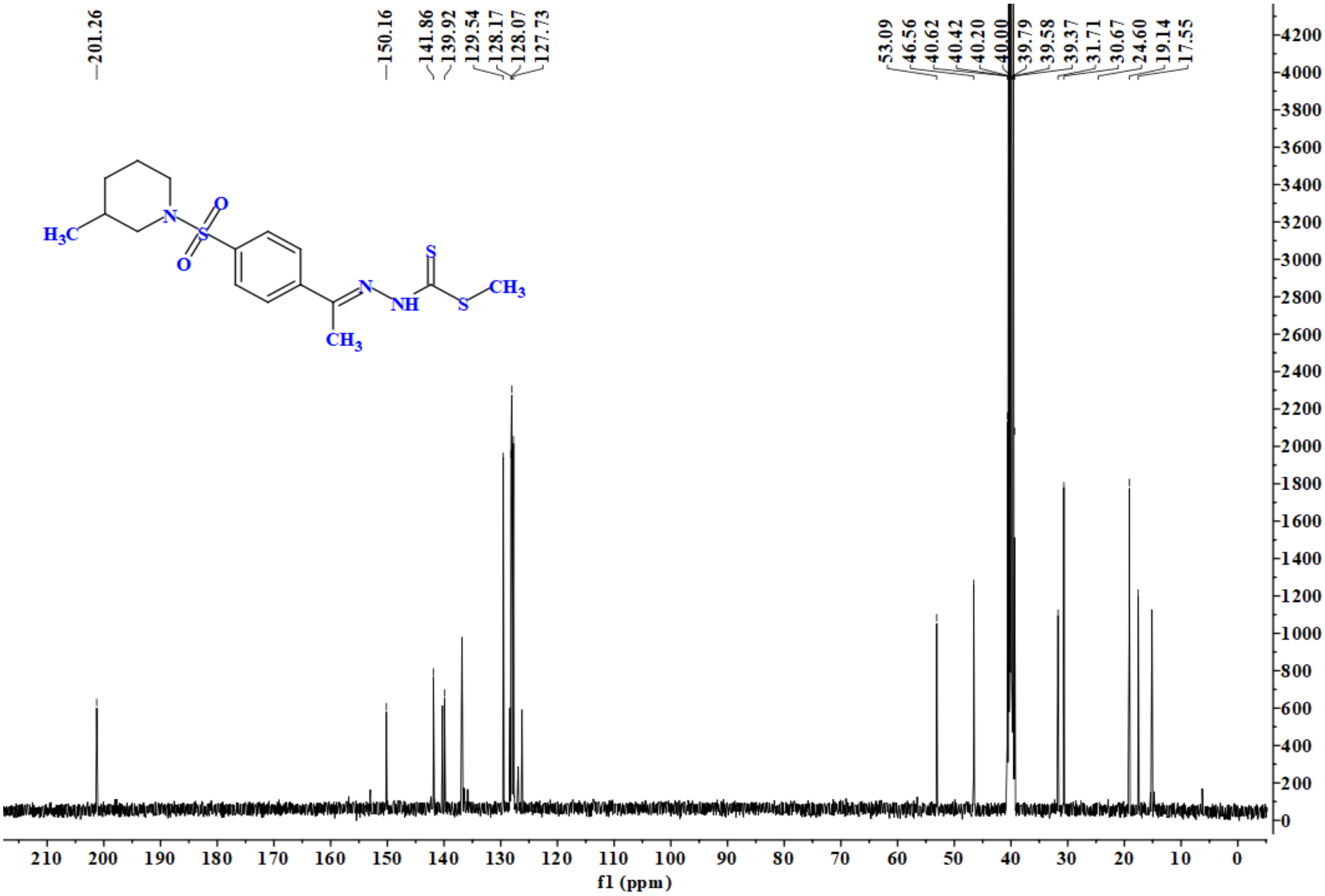

Fig. S9: ^{13}C NMR (DMSO-d$_6$) **of *starting material* 9**

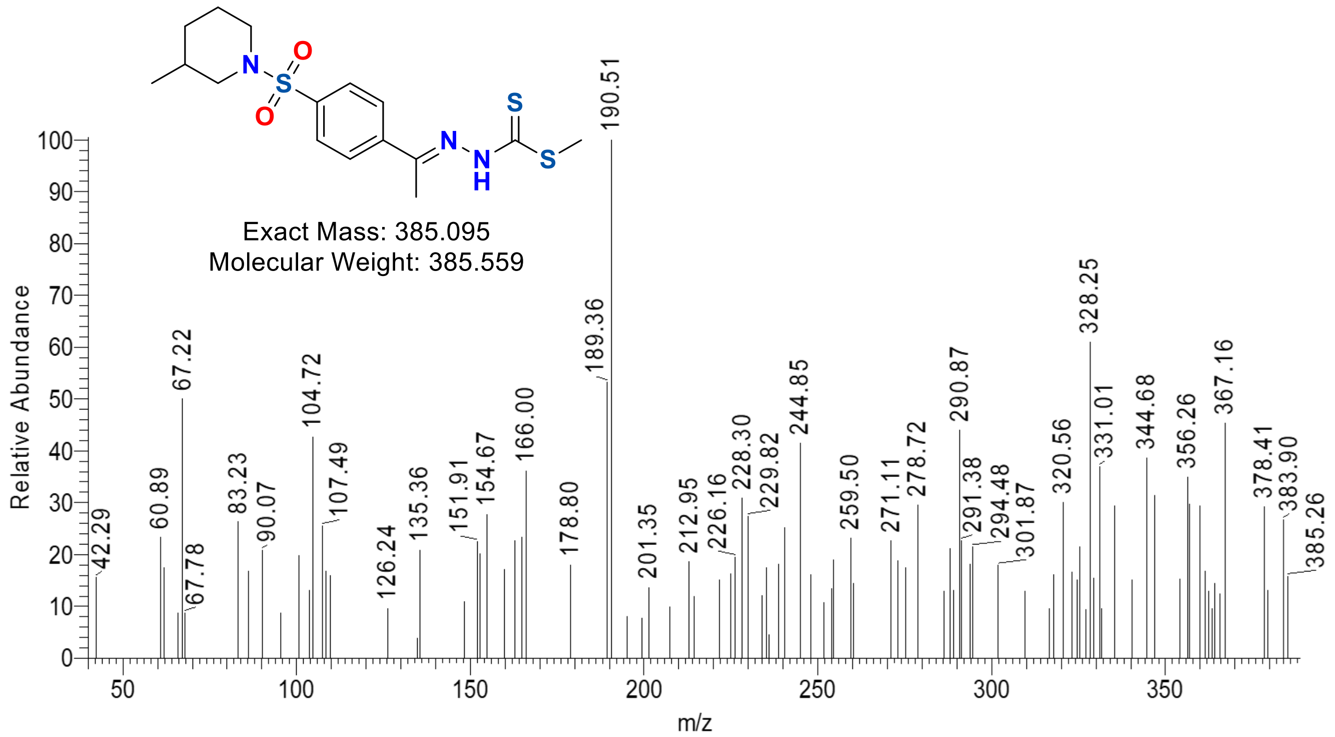

Fig S10: Mass spectrum of *starting material* 9

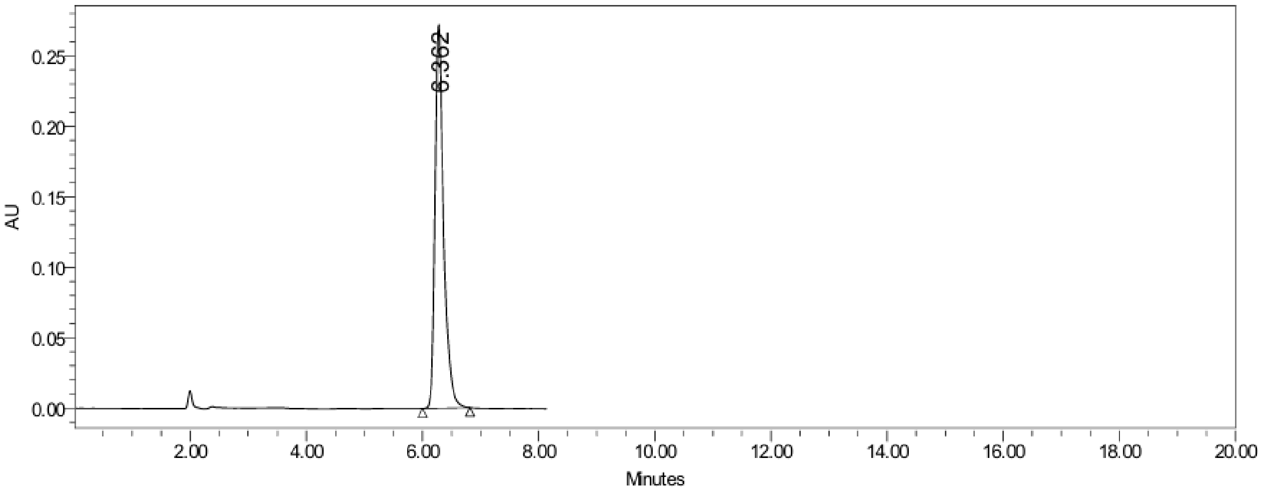

	RT	Area	% Area	USP Plate Count	USP Tailing	K Prime
1	6.362	56735	100.00	4009.65	1.10	5.06

Fig S11: HPLC chromatogram of *starting material* 9

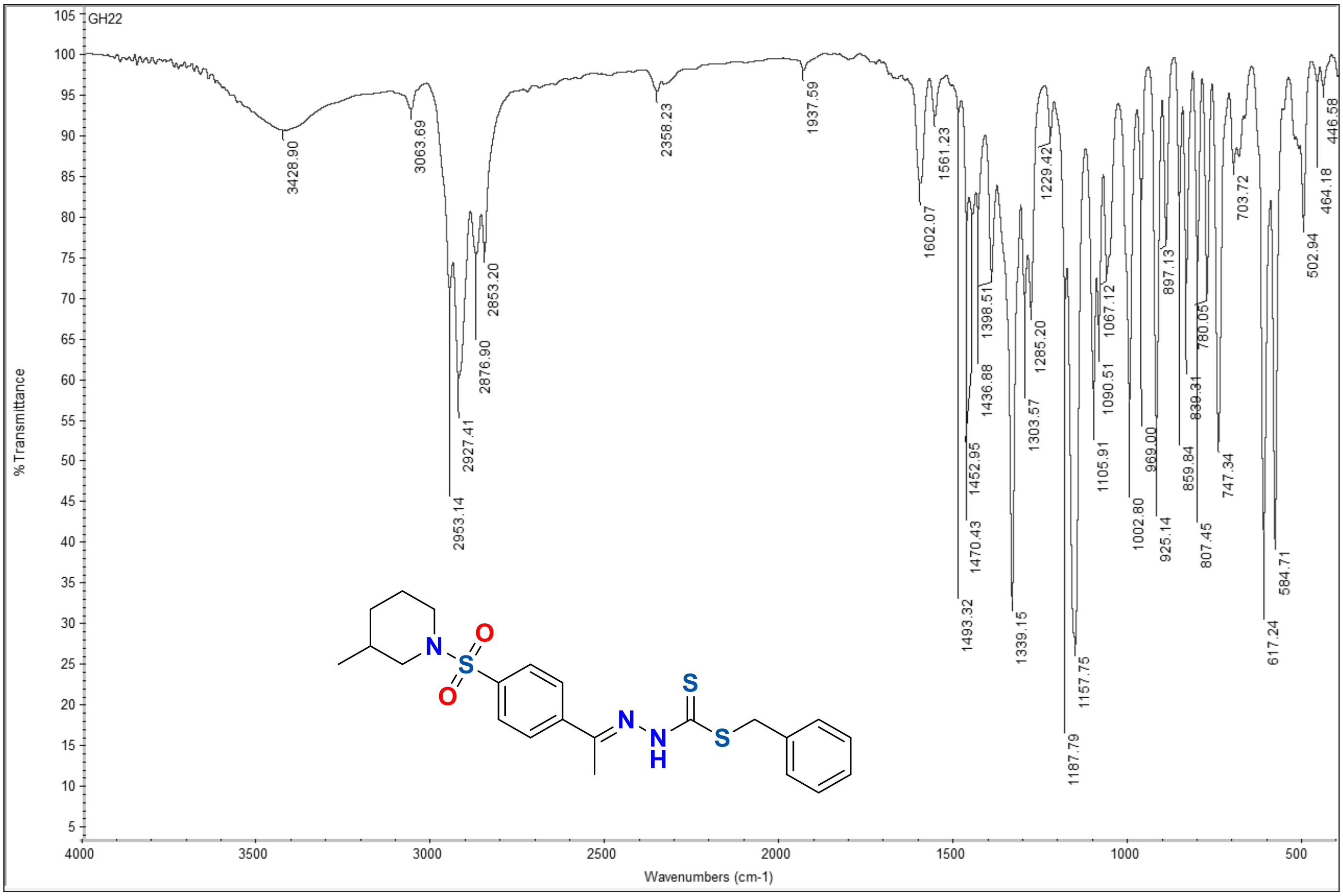

*Fig S12: FT-IR (*KBr, ν cm^{-1}) *of starting material 10.*

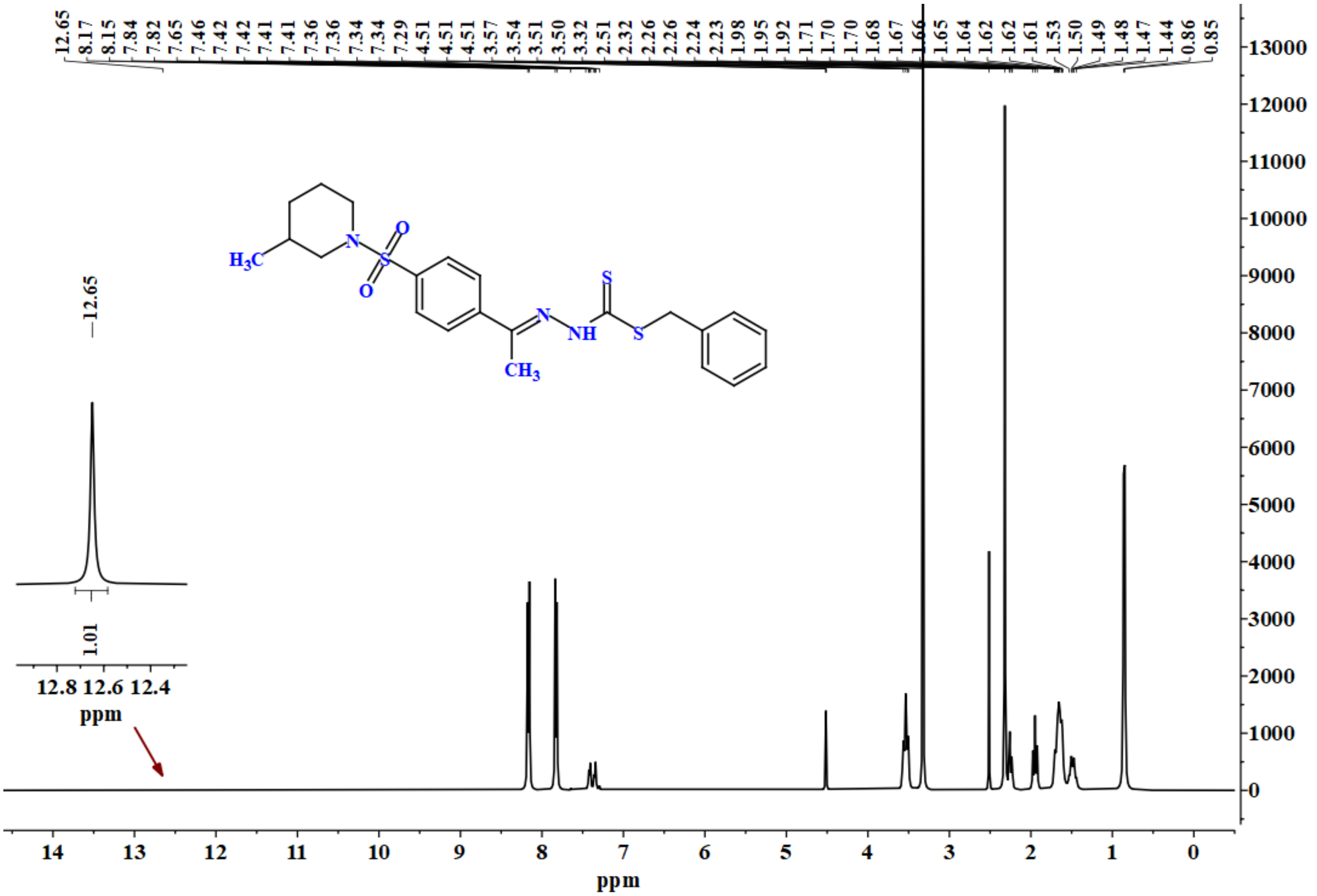

Fig S13: ^{1}H NMR (*DMSO-d$_6$) *of starting material 10

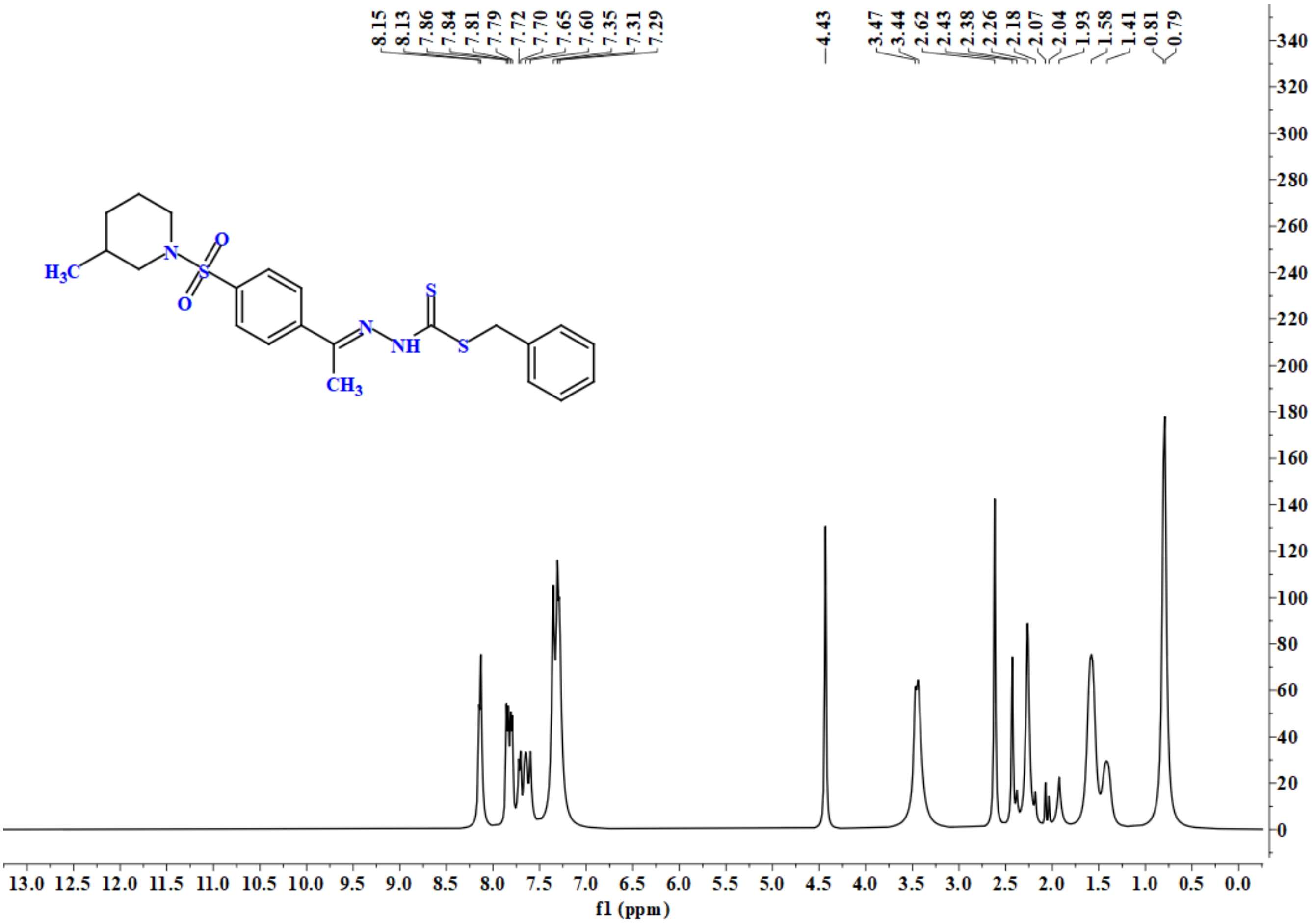

*Fig S14: ^{1}H NMR / D_2O (*DMSO-d$_6$) *of starting material 10*

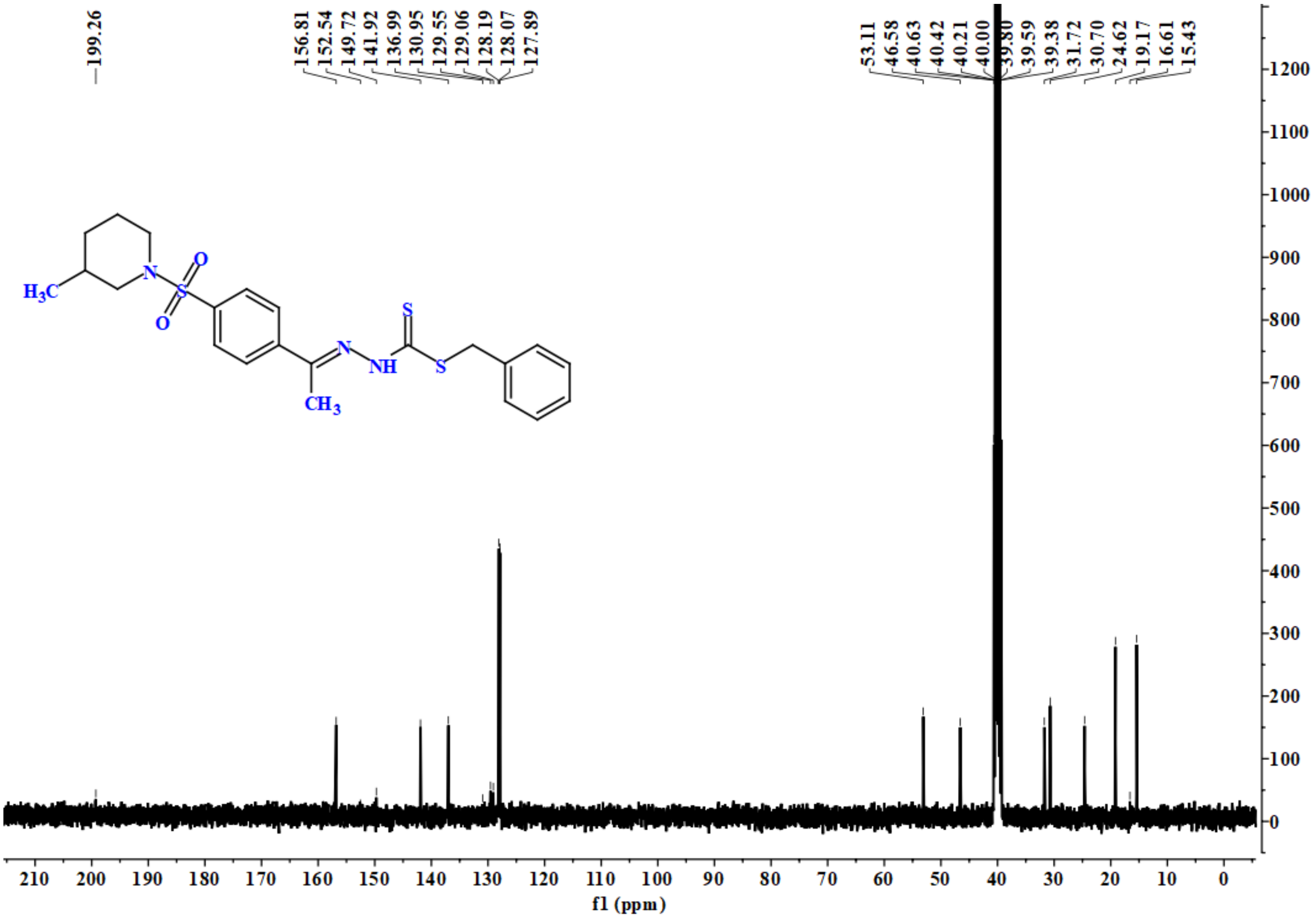

Fig S15: 13C NMR (DMSO) **of *starting material* 10**

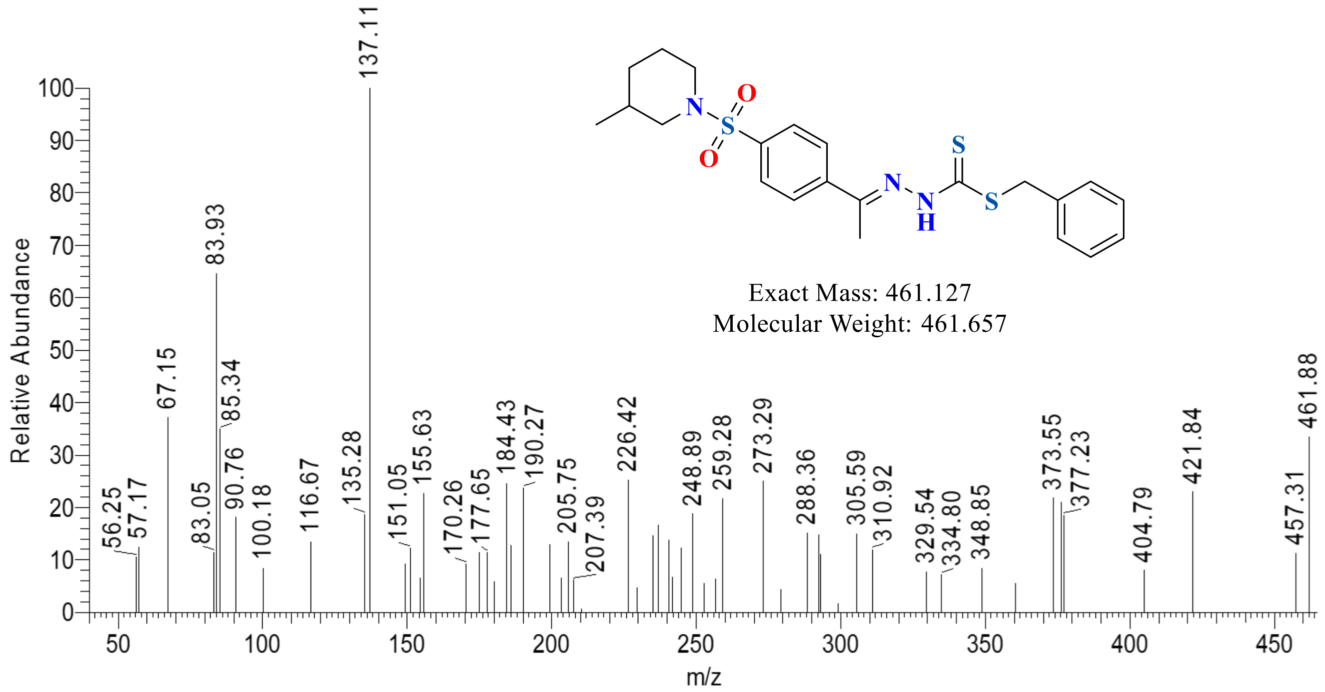

Fig S16: Mass spectrum of *starting material* 10

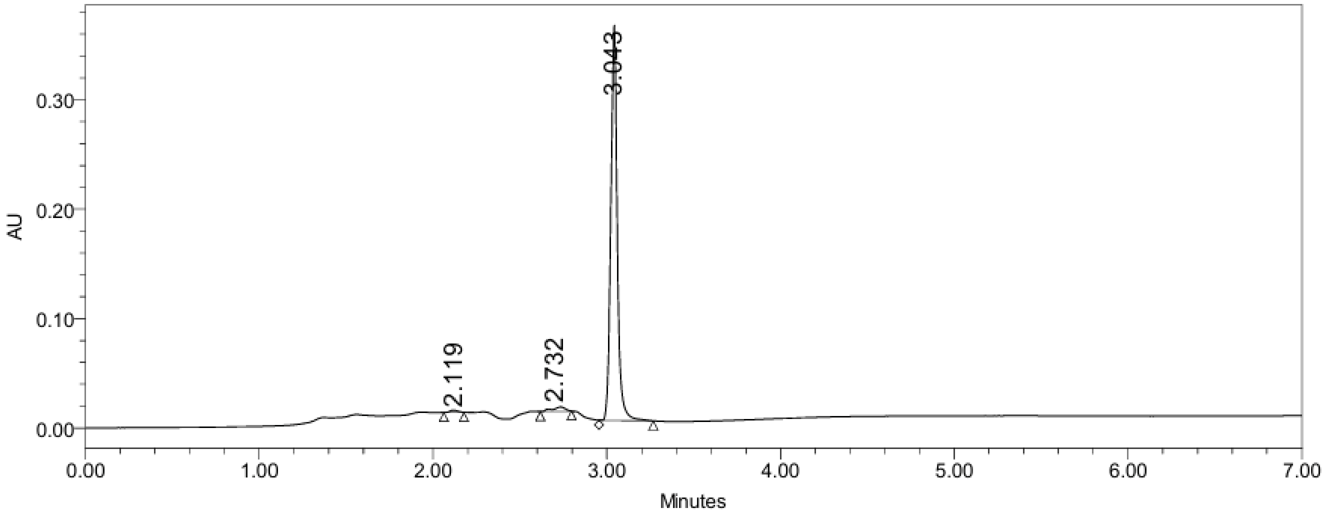

	RT	Area	% Area	USP Plate Count	USP Tailing	K Prime
1	2.119	11337	1.15	9140.53	1.16	1.02
2	2.732	33031	3.37	8114.66	0.86	1.60
3	3.043	933517	95.47	31500.06	1.08	1.90

Fig S17: HPLC chromatogram of *starting material* 10

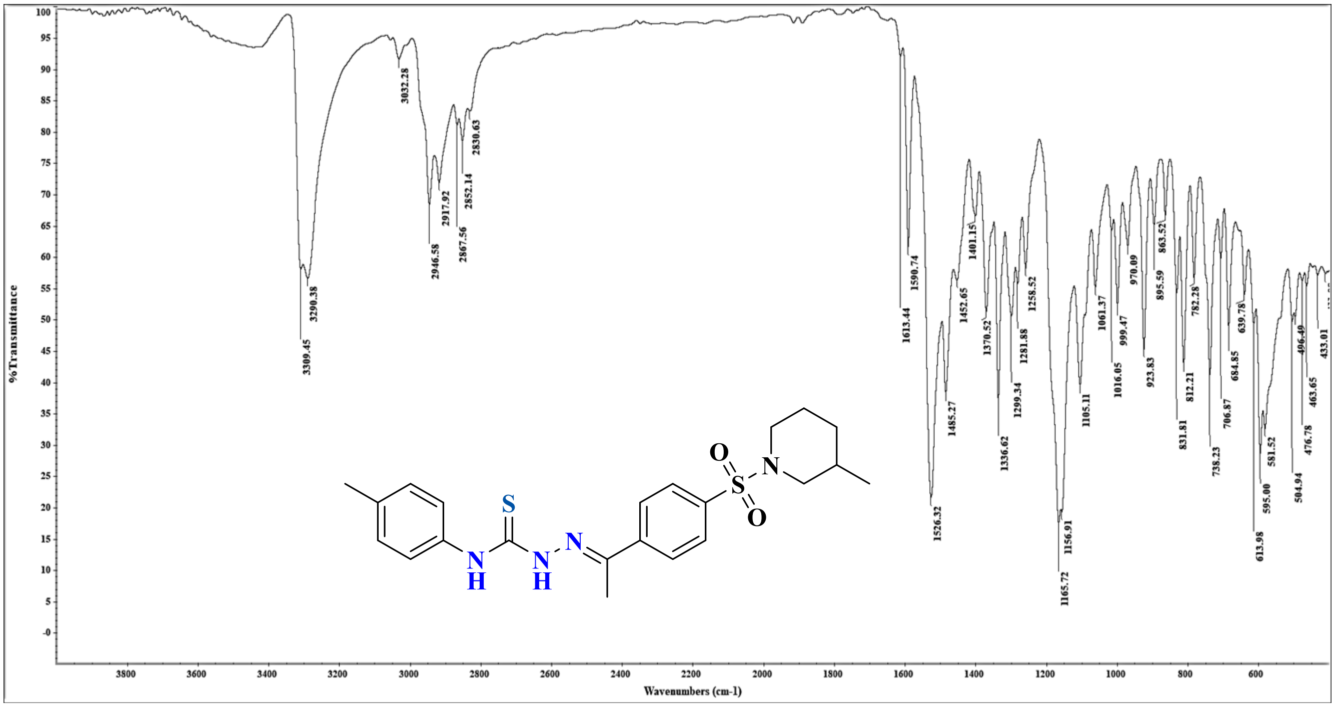

Fig S18: FT-IR (*KBr, ν cm^{-1}*) of intermediate 11.

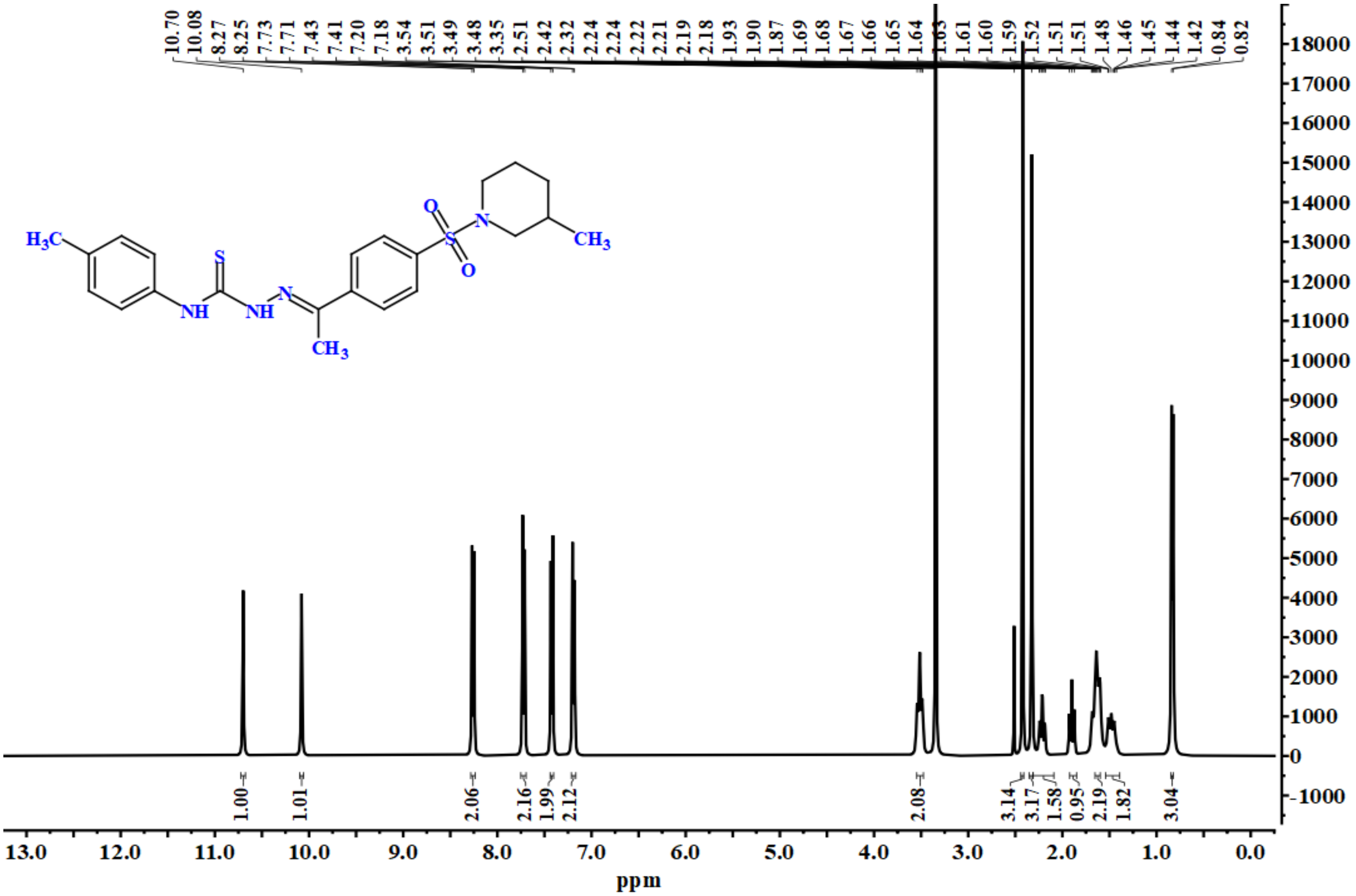

Fig S19: ¹H NMR (*DMSO-d_6) *of intermediate 11

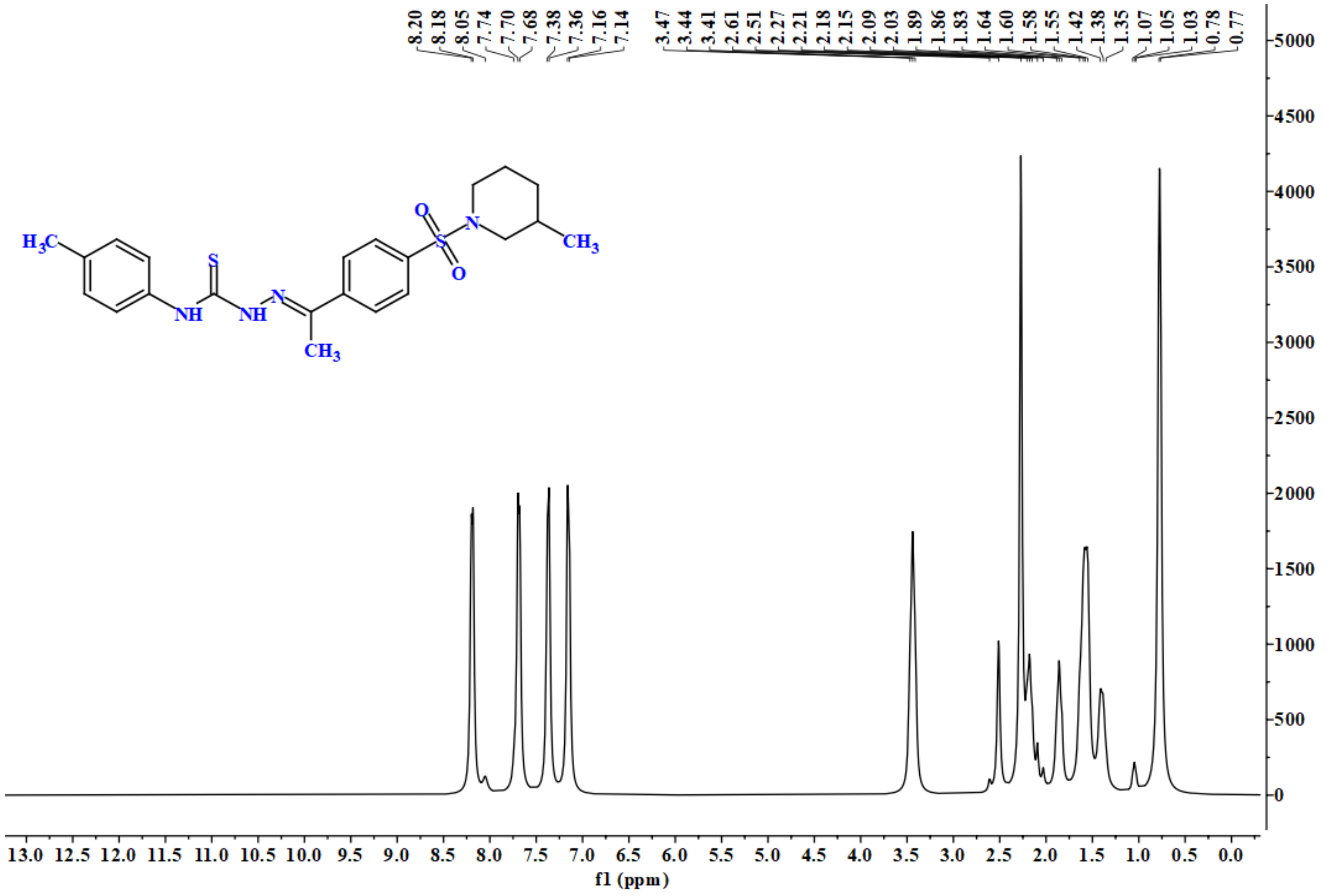

*Fig S20: ^{1}H NMR / D$_2$O (*DMSO-d$_6$) *of intermediate 11*

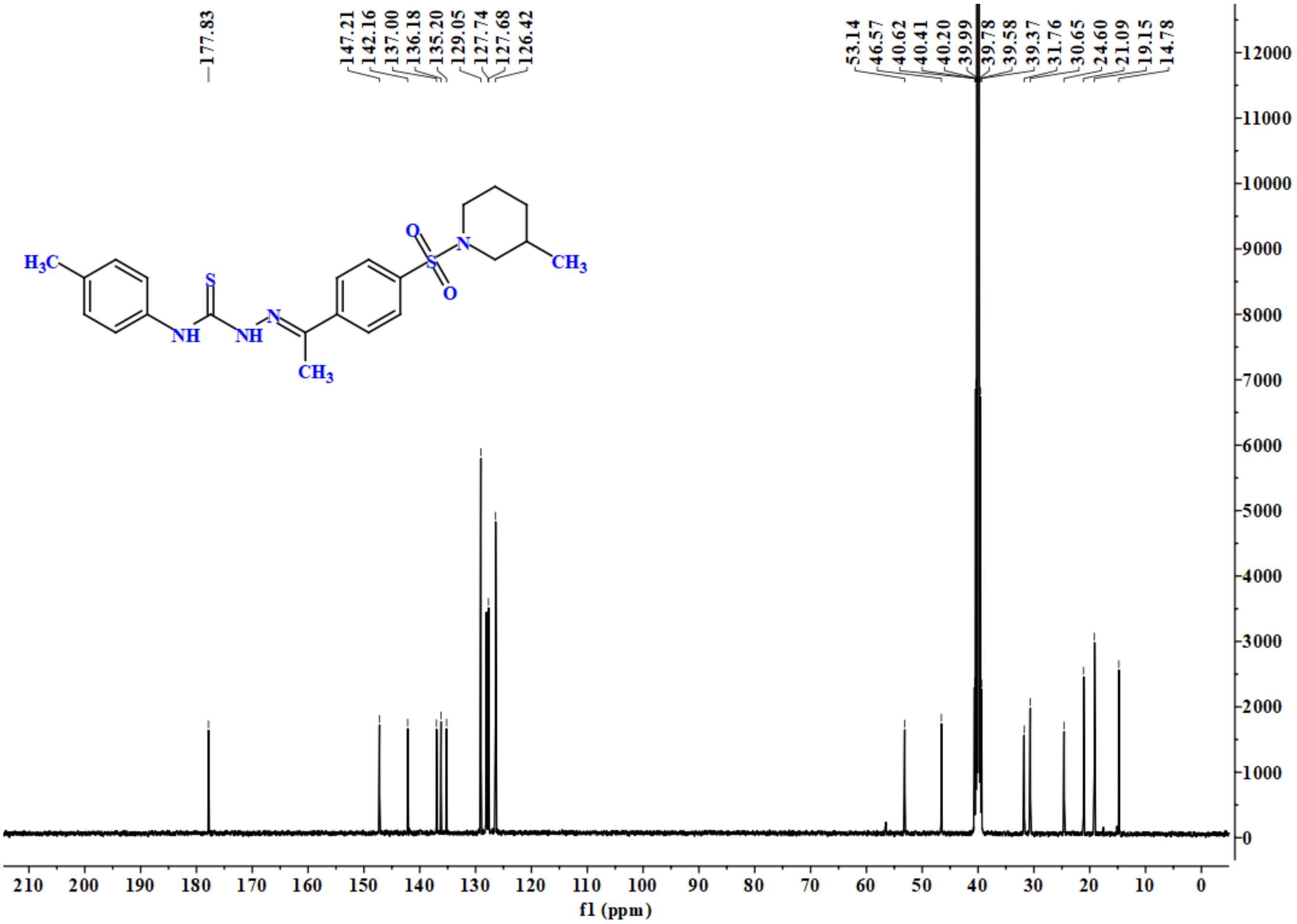

Fig S21: ^{13}C NMR (**DMSO-d6**) *of intermediate 11*

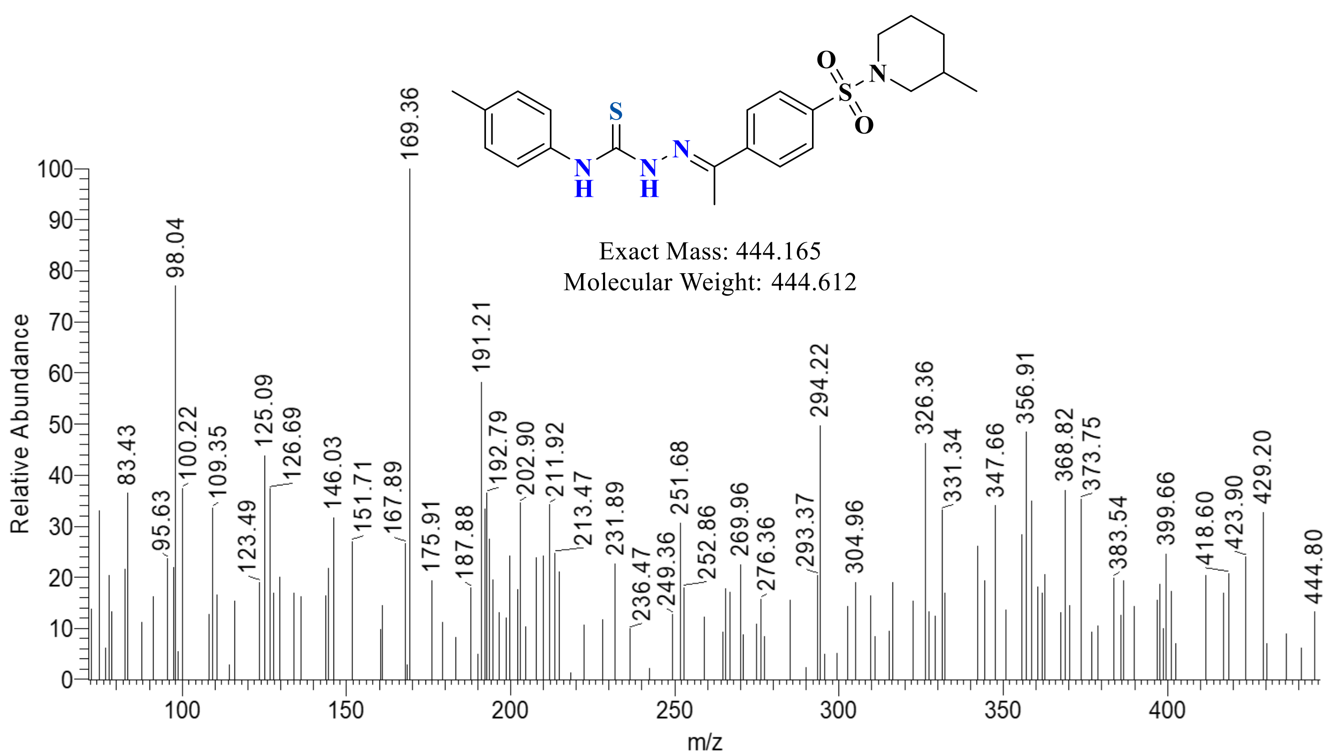

Fig S22: Mass spectrum of intermediate 11

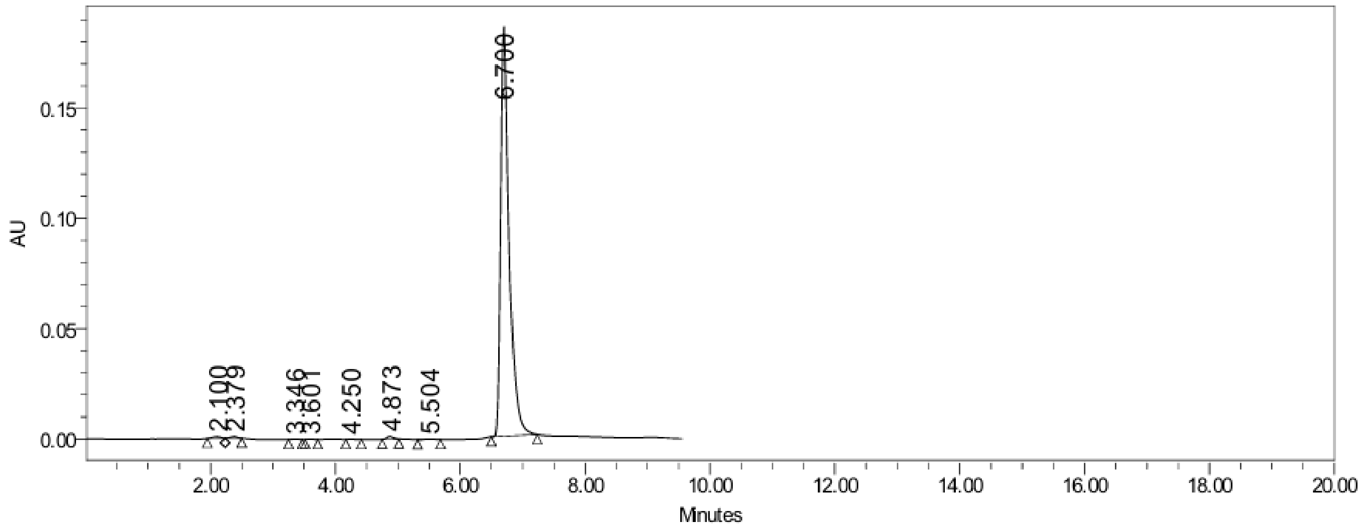

	RT	Area	% Area	USP Plate Count	USP Tailing	K Prime
1	2.100	7517	0.42	1057.57	0.94	1.00
2	2.379	6065	0.34	1862.63	0.93	1.27
3	3.346	810	0.05	5959.14	1.22	2.19
4	3.601	551	0.03	10159.84	1.07	2.43
5	4.250	563	0.03	13217.21	1.54	3.05
6	4.873	8990	0.50	10169.04	1.11	3.64
7	5.504	1815	0.10	8074.21	0.98	4.24
8	6.700	1769126	98.53	12907.97	1.64	5.38

Fig S23: HPLC chromatogram of intermediate 11

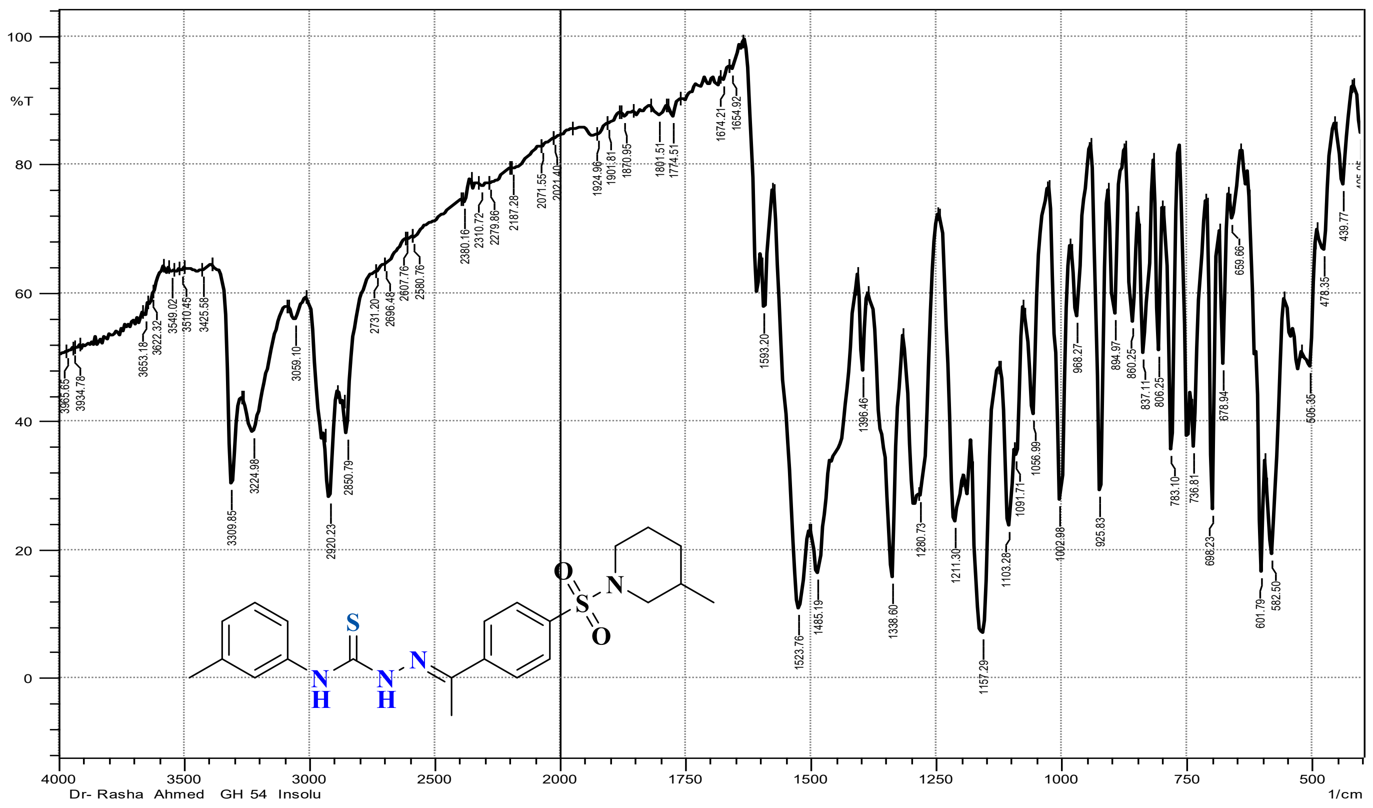

*Fig S24: FT-IR (***KBr, ν cm**$^{-1}$**)** *of intermediate 12.*

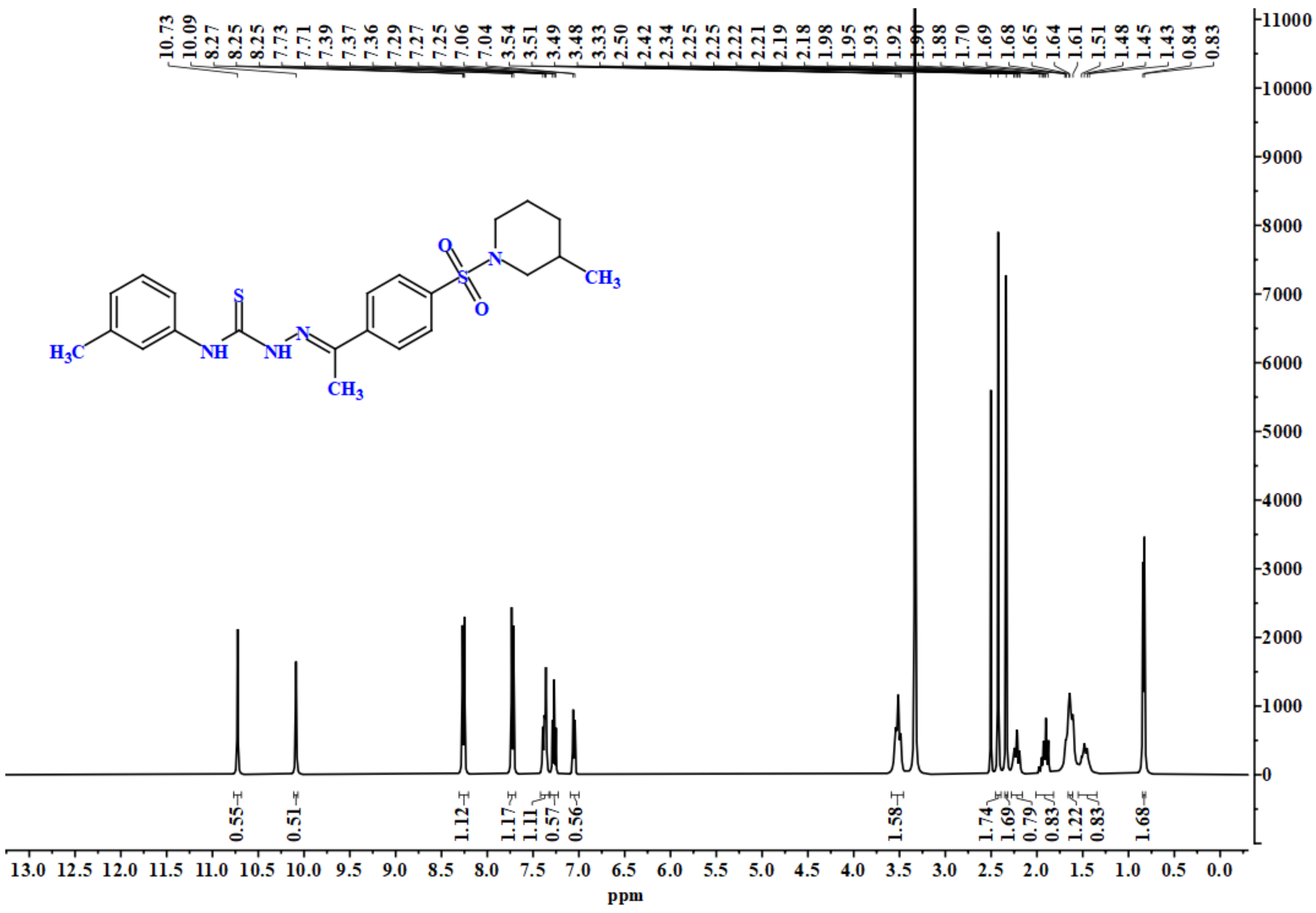

*Fig S25: ^{1}H NMR (*DMSO-d$_6$) *of intermediate 12*

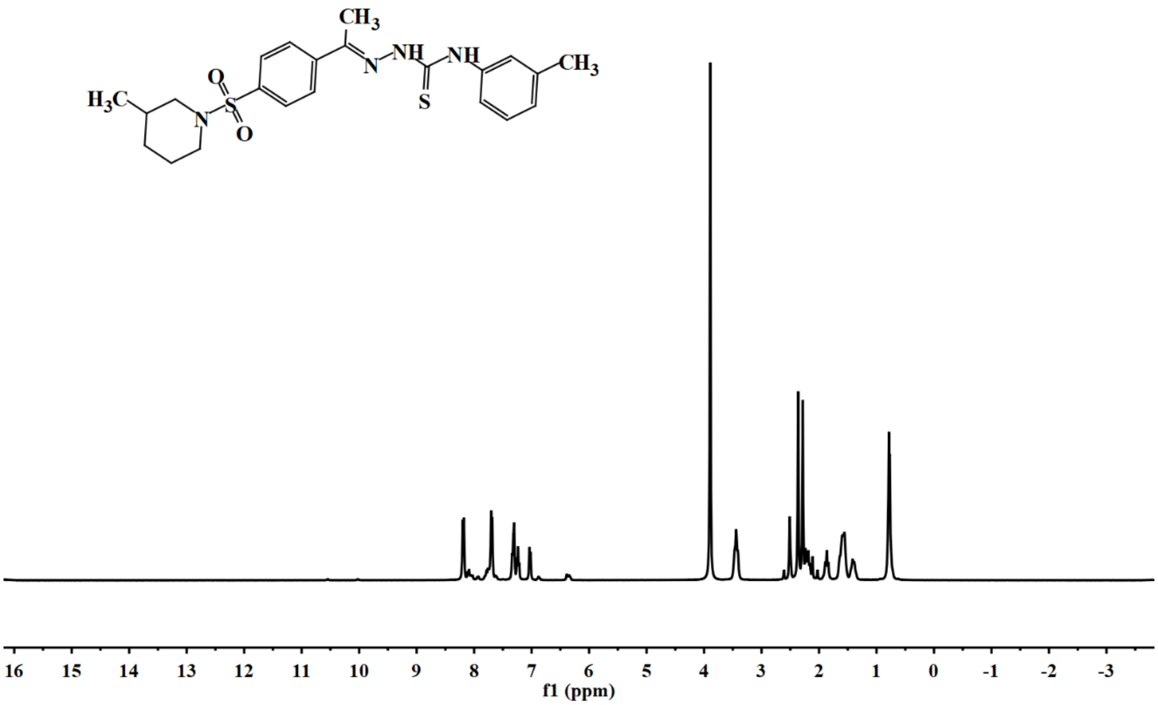

Fig S26: ^{1}H NMR / D_2O (*DMSO-d_6) *of intermediate 12

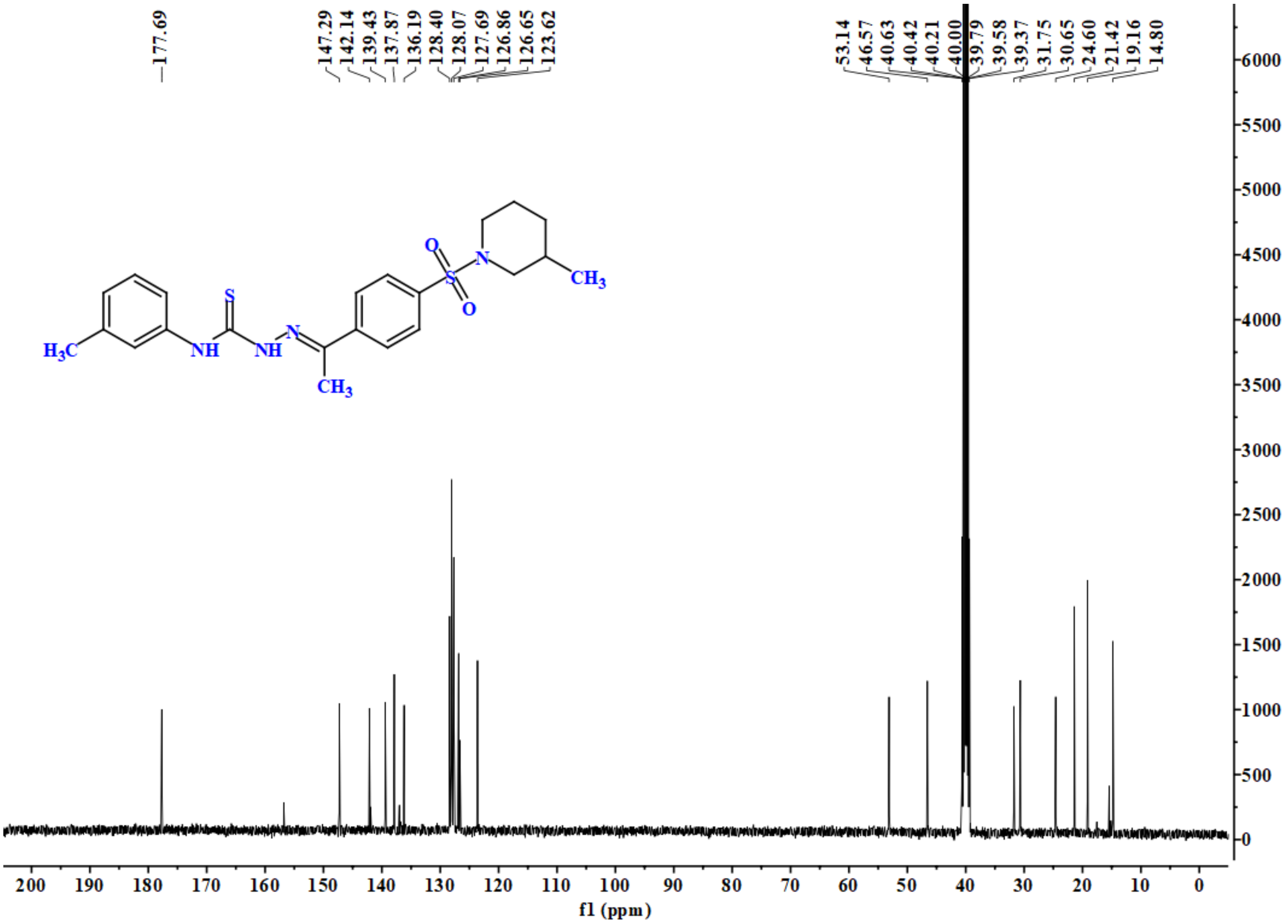

Fig S27: ^{13}C NMR (DMSO-d$_6$) *of intermediate 12*

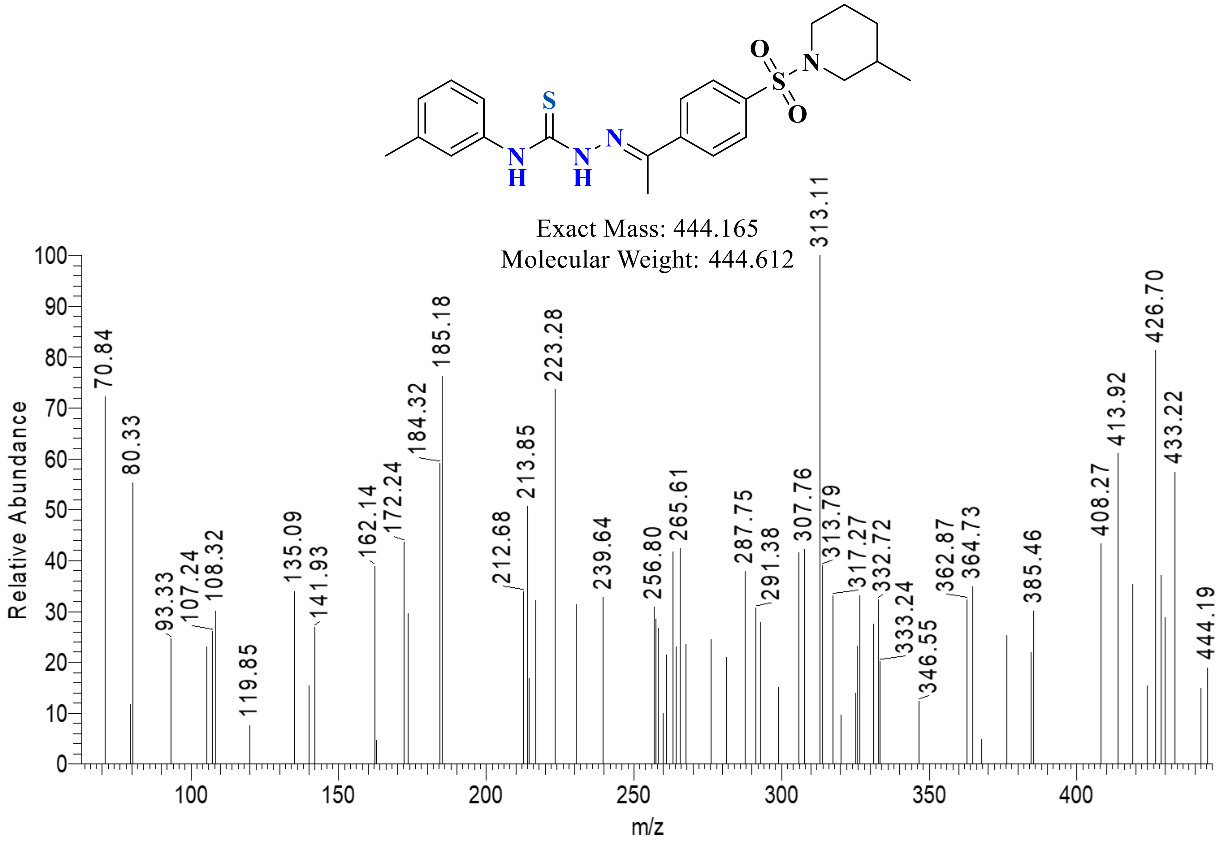

Fig S28: Mass spectrum of intermediate 12

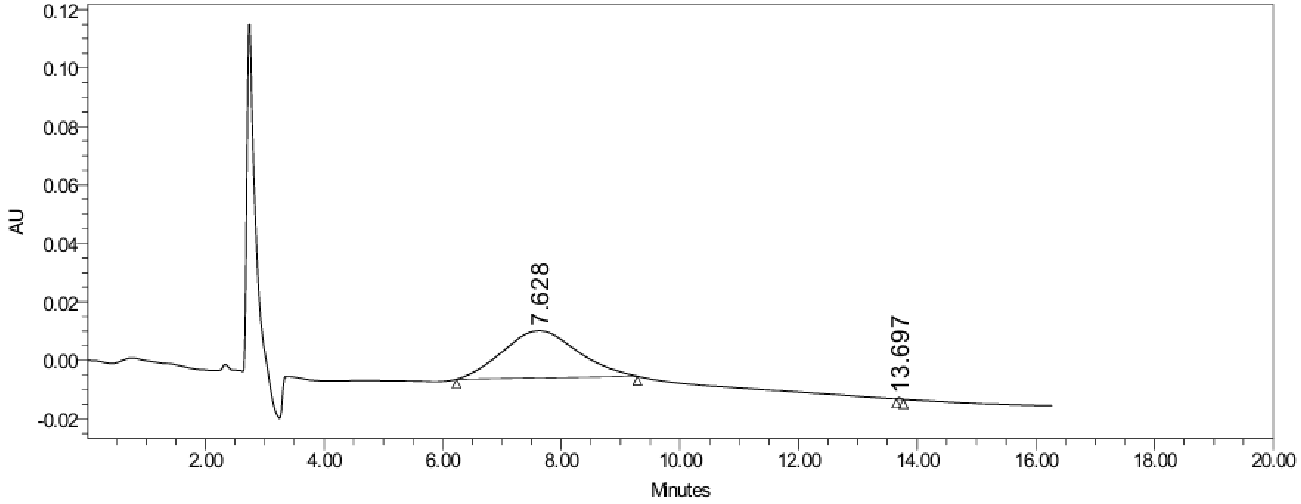

	RT	Area	% Area	USP Plate Count	USP Tailing	K Prime
1	7.628	1410189	99.89	160.31	1.09	6.26
2	13.697	1519	0.11	298264.01	1.10	12.04

Fig S29: HPLC chromatogram of intermediate 12

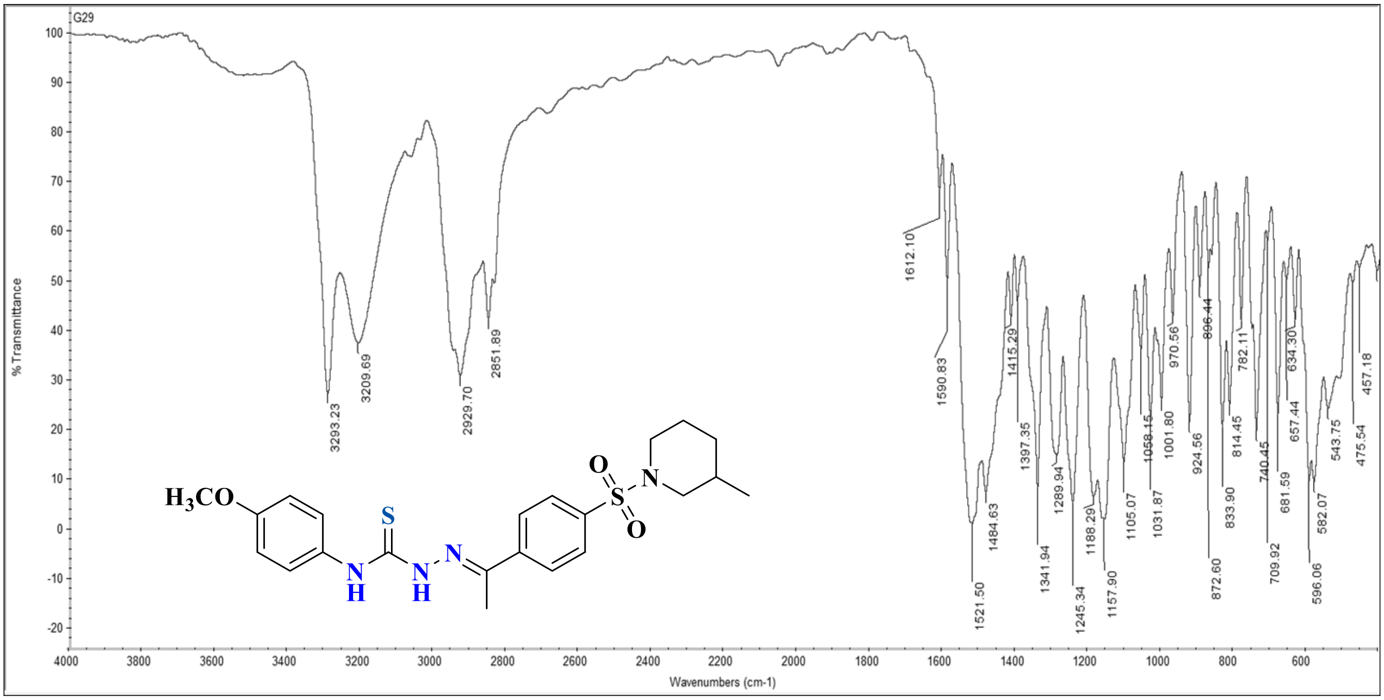

Fig S30: FT-IR (*KBr, ν cm^{-1}) *of* intermediate *13.

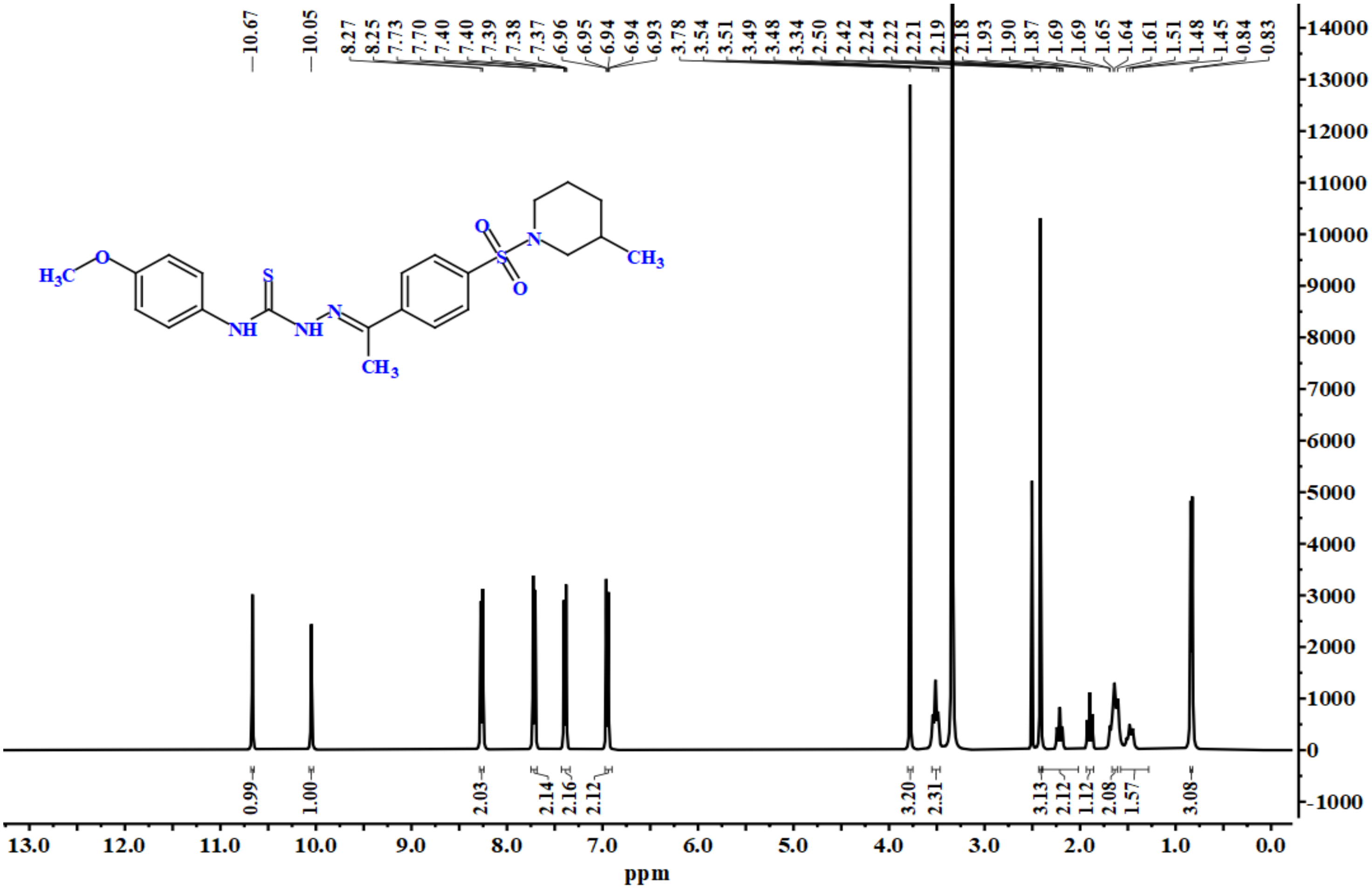

*Fig S31: ^{1}H NMR (*DMSO-d$_6$) ***of*** **intermediate *13***

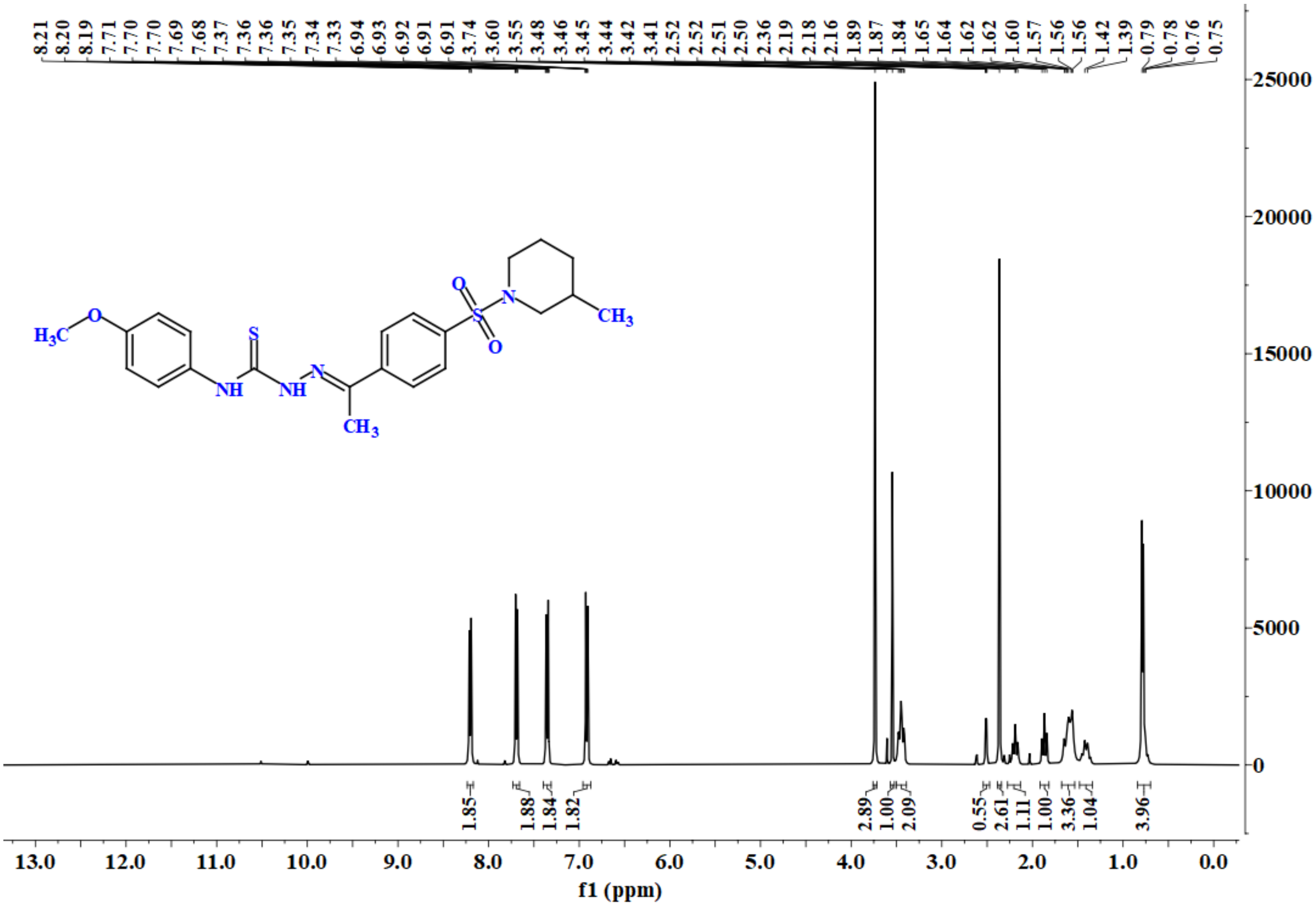

*Fig S32: ^{1}H NMR / D_2O (*DMSO-d6) *of* intermediate *13*

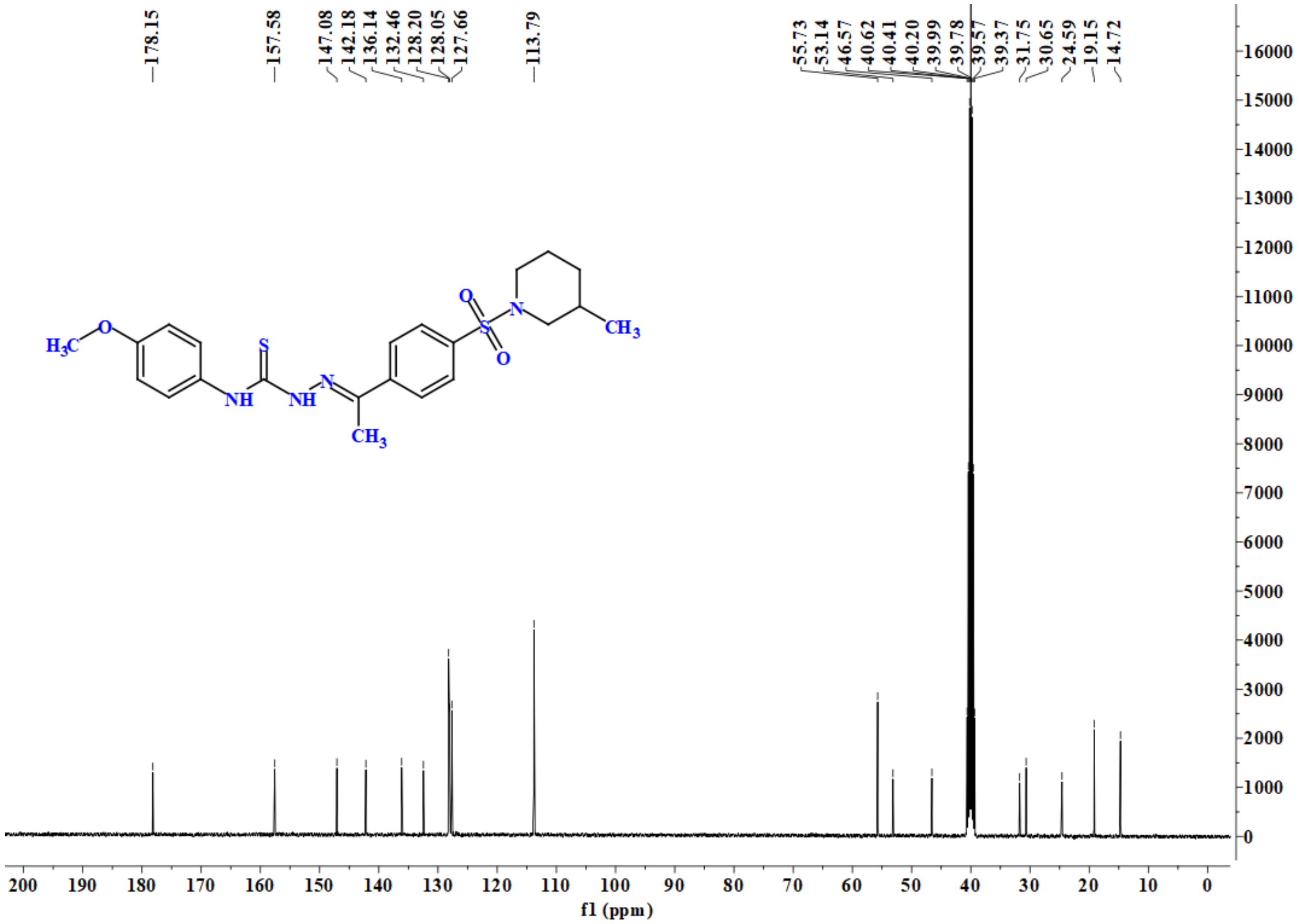

Fig S33: ^{13}C NMR (**DMSO-d6**) ***of* intermediate *13***

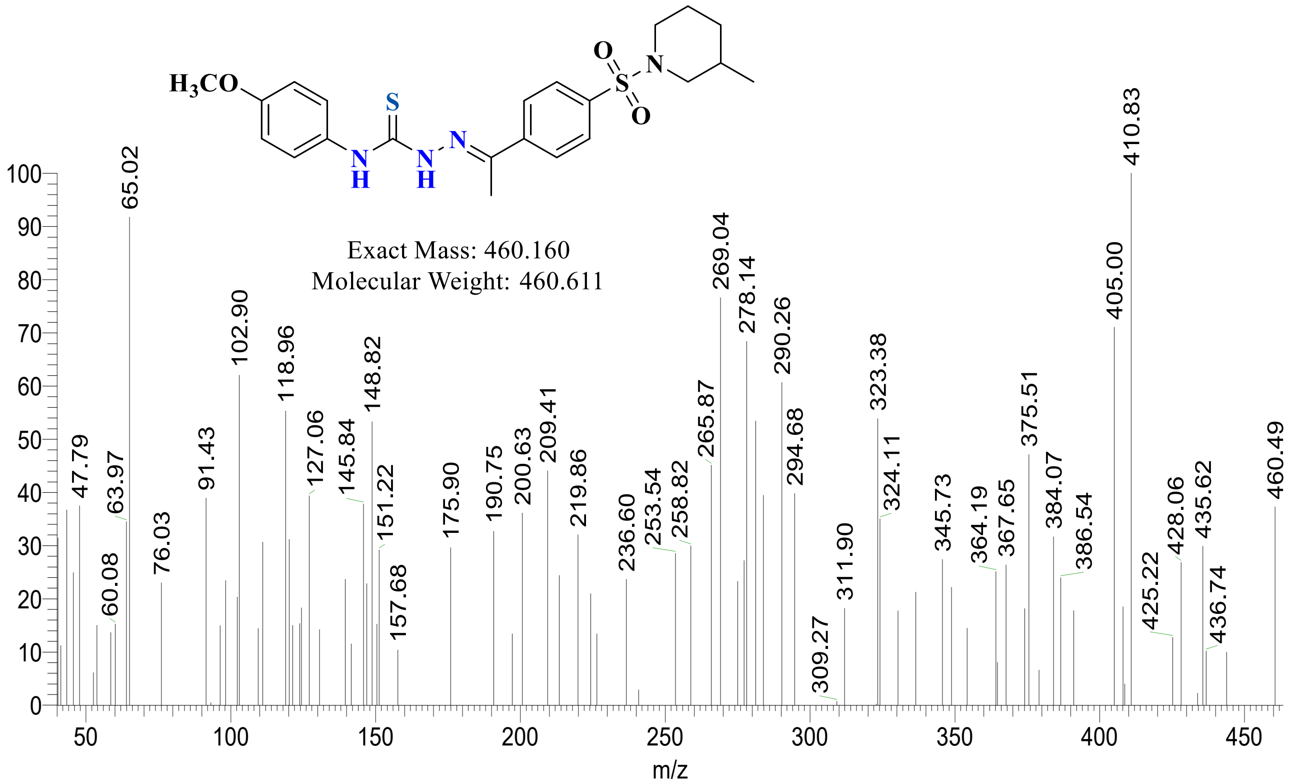

Fig S34: Mass spectrum of intermediate 13

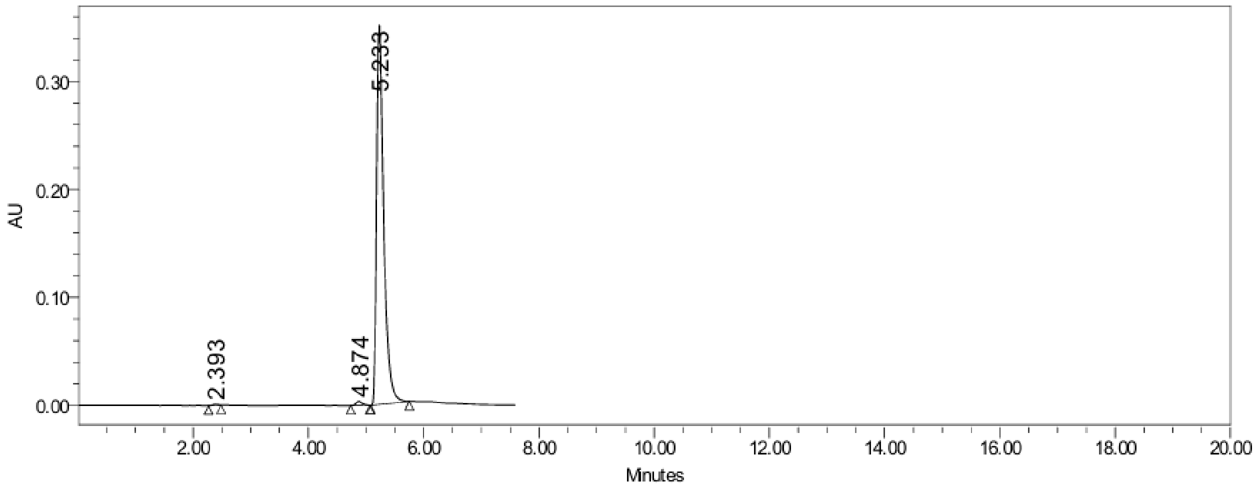

	RT	Area	% Area	USP Plate Count	USP Tailing	K Prime
1	2.393	5011	0.17	3381.54	0.89	1.28
2	4.874	28097	0.93	9628.99	1.24	3.64
3	5.233	2973927	98.90	9425.99	1.59	3.98

Fig S35: HPLC chromatogram of intermediate 13

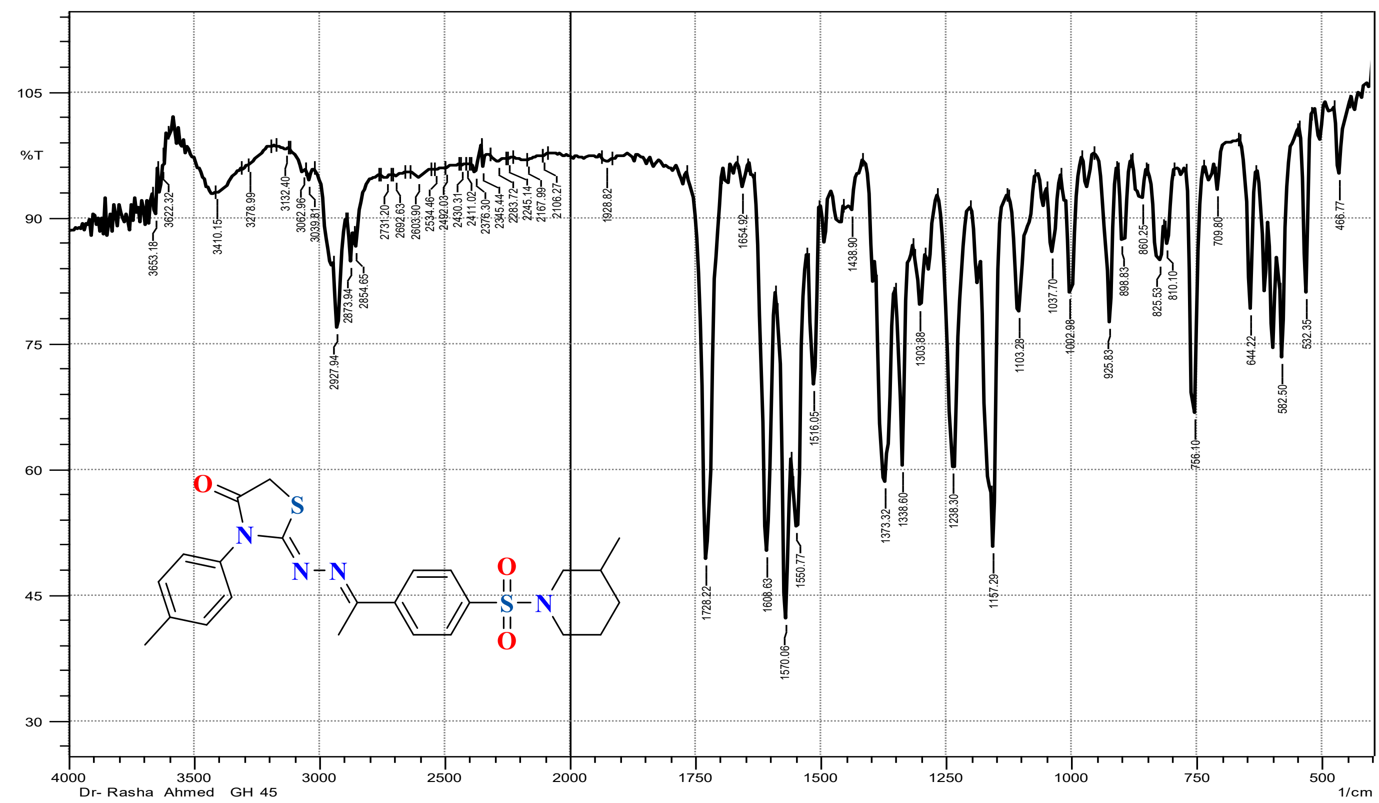

*Fig S36: FT-IR (*KBr, ν cm^{-1}) *of* compound *14.*

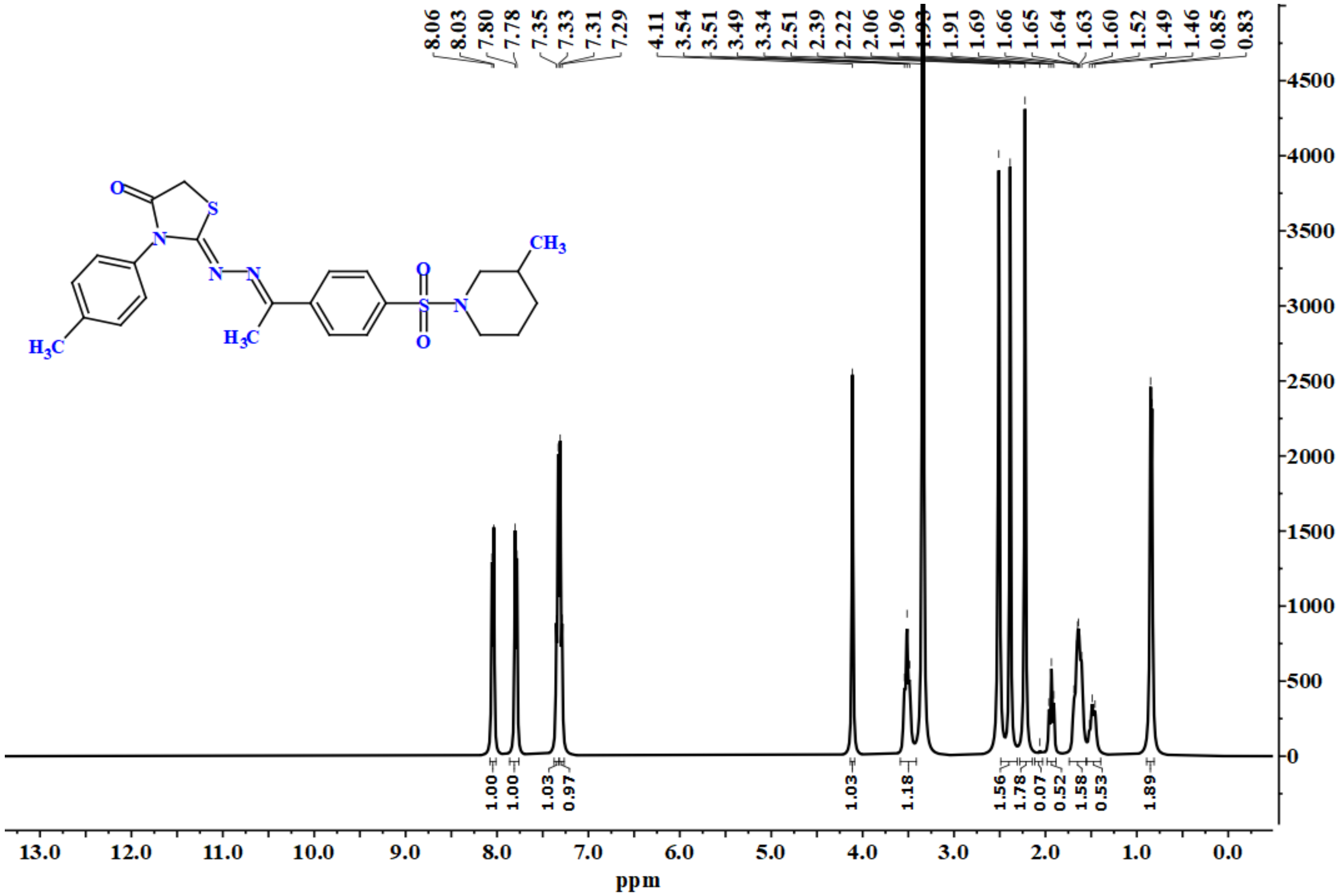

*Fig S37: ^{1}H NMR (*DMSO-d$_6$) *of compound 14*

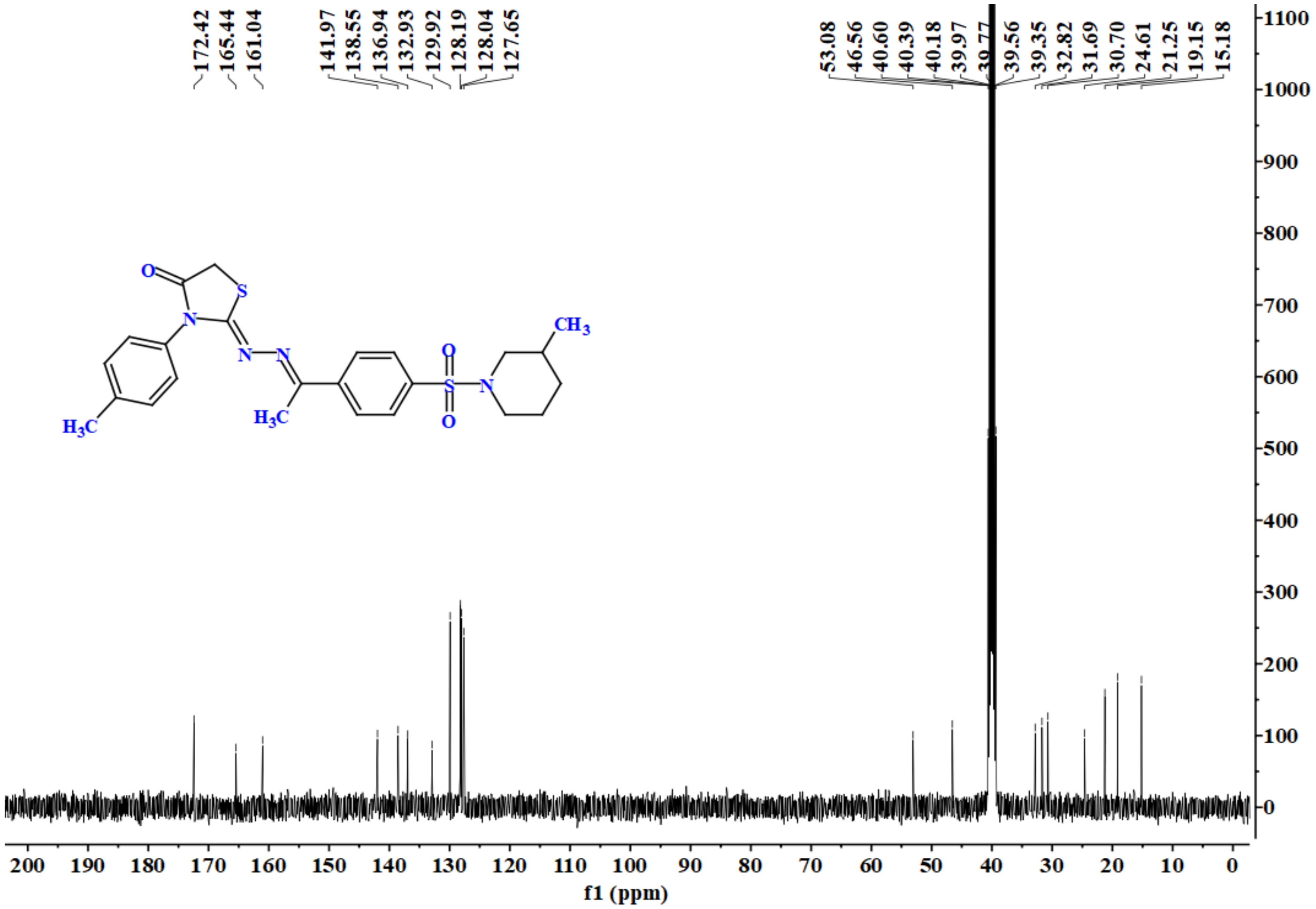

*Fig S38: ^{13}C NMR (*DMSO-d6) *of* Compound 14

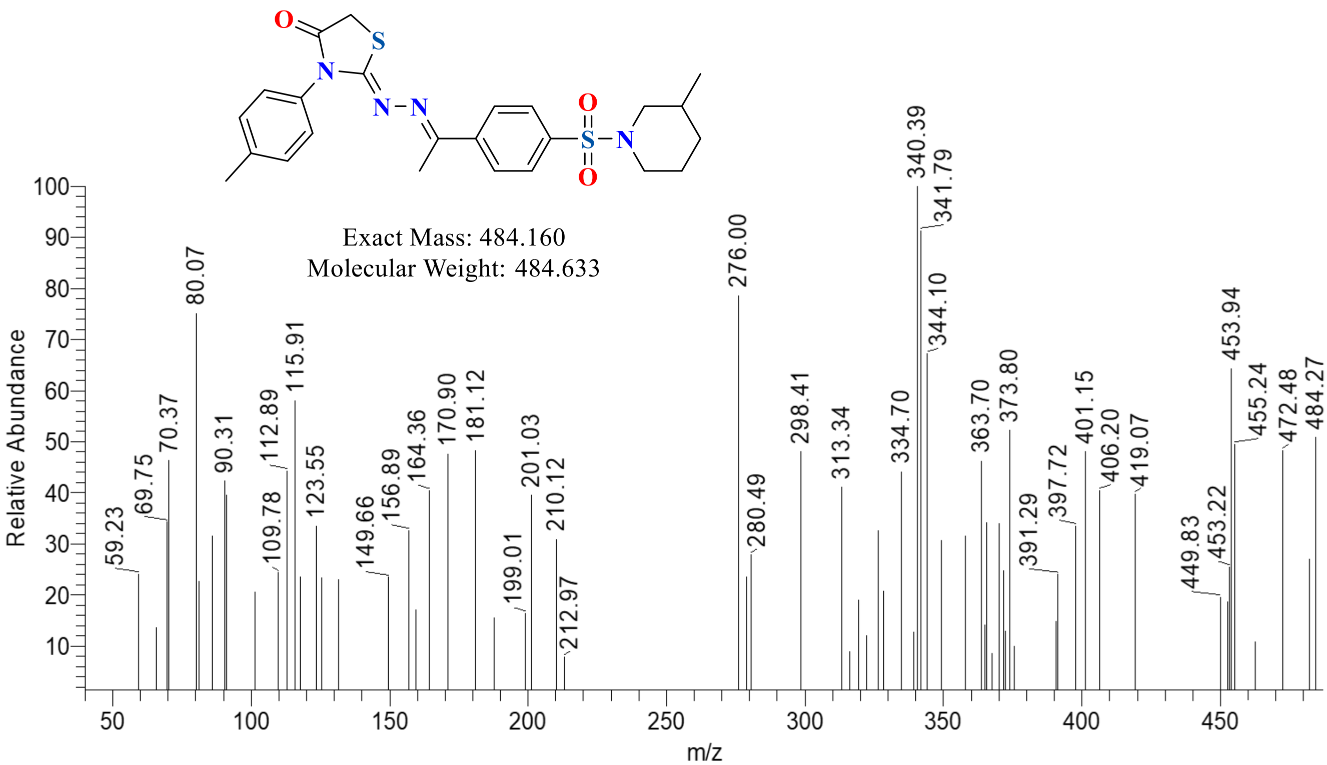

Fig S39: Mass spectrum of compound 14

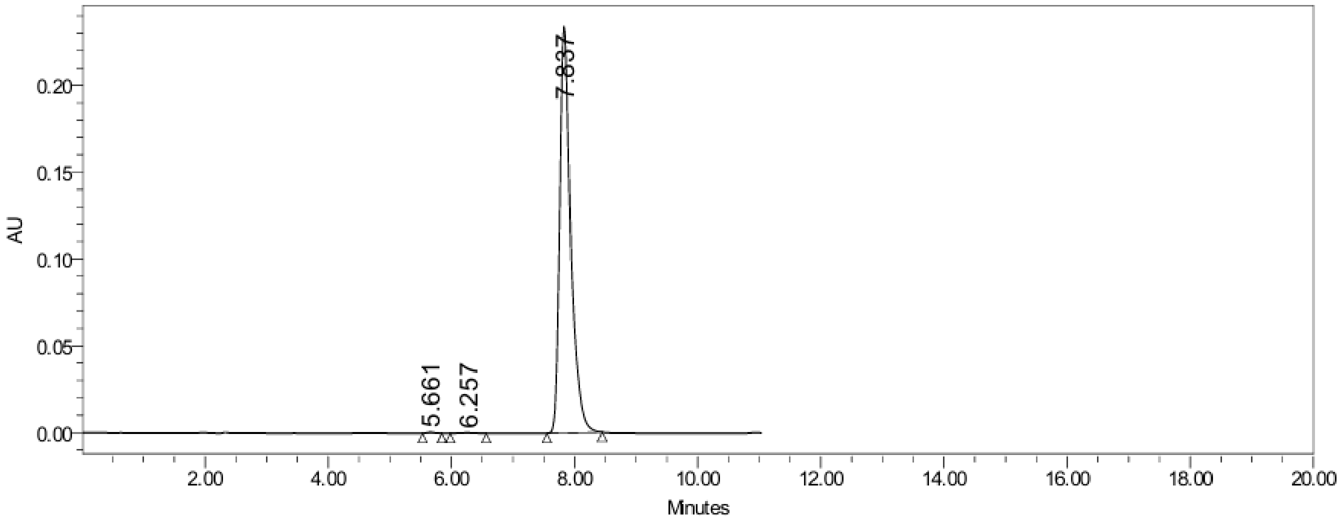

	RT	Area	% Area	USP Plate Count	USP Tailing	K Prime
1	5.661	5394	0.19	10462.79	1.18	4.39
2	6.257	4721	0.16	5512.69	1.03	4.96
3	7.837	2898385	99.65	10156.97	1.45	6.46

Fig S40: HPLC chromatogram of compound 14

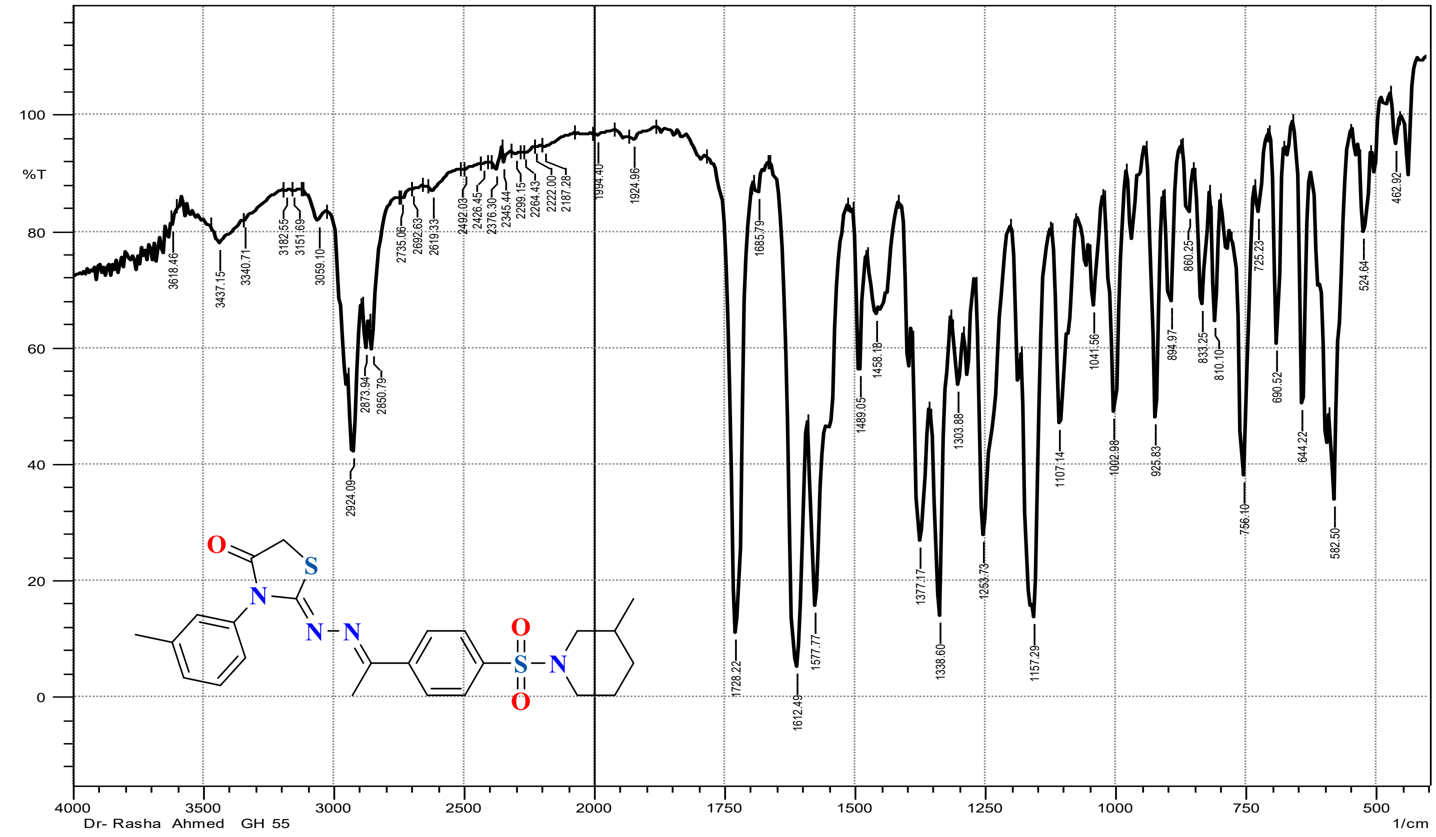

*Fig S41: FT-IR (*KBr, ν cm^{-1}) *of* compound *15.*

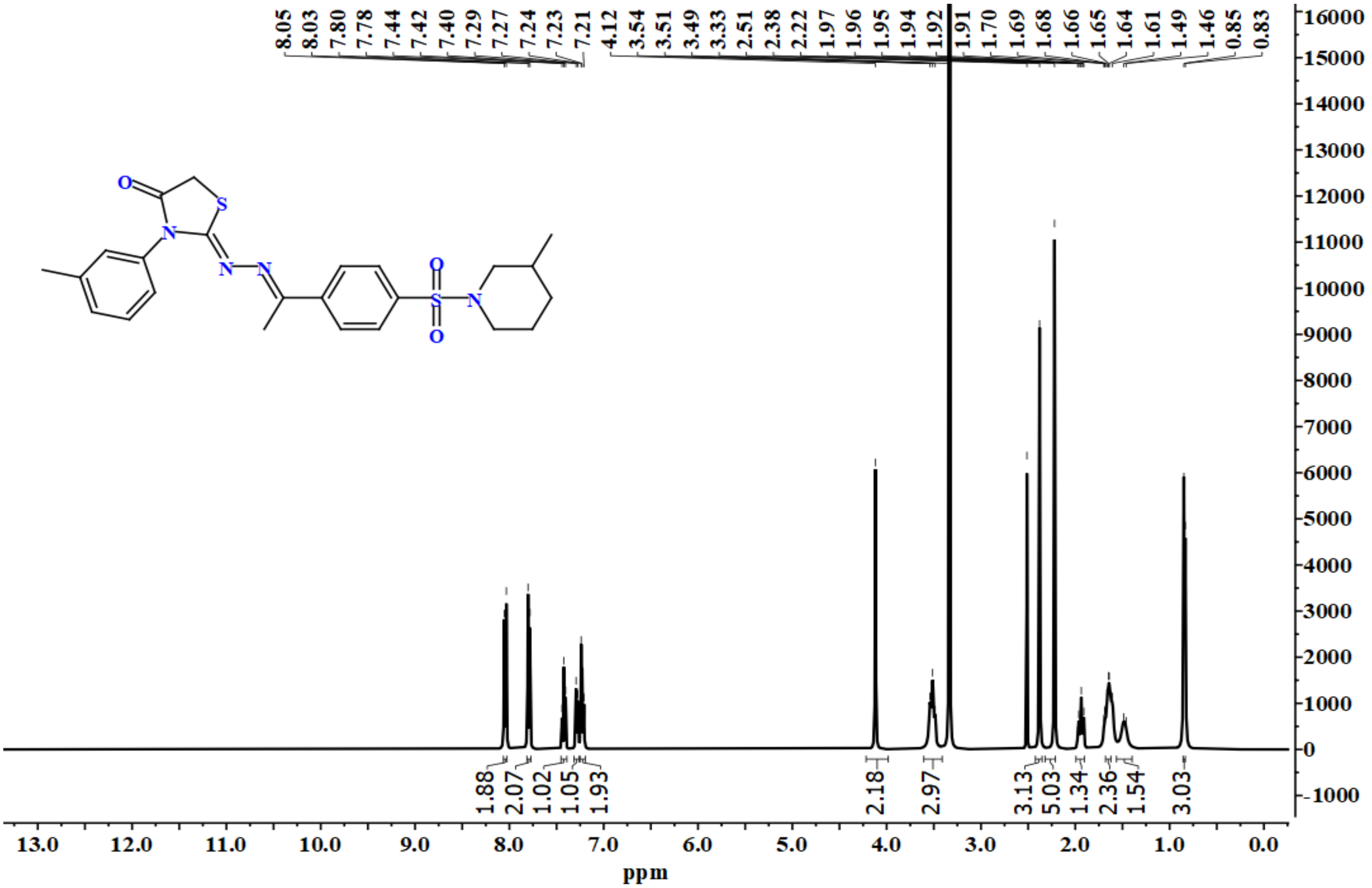

Fig S42: ^{1}H NMR (*DMSO-d$_6$) *of compound 15

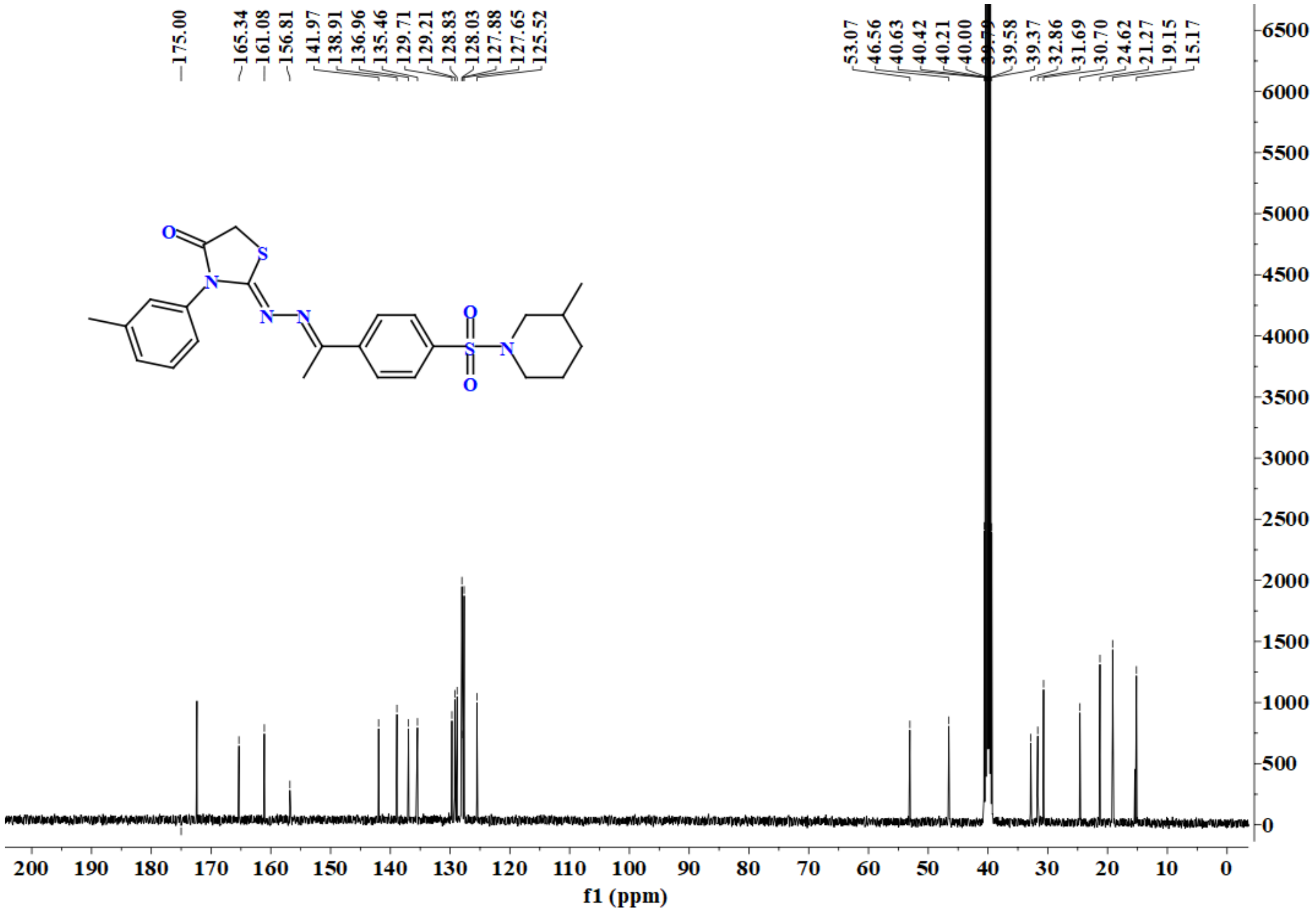

*Fig S43: ^{13}C NMR (***DMSO-d**$_6$**)** *of* **compound** *15*

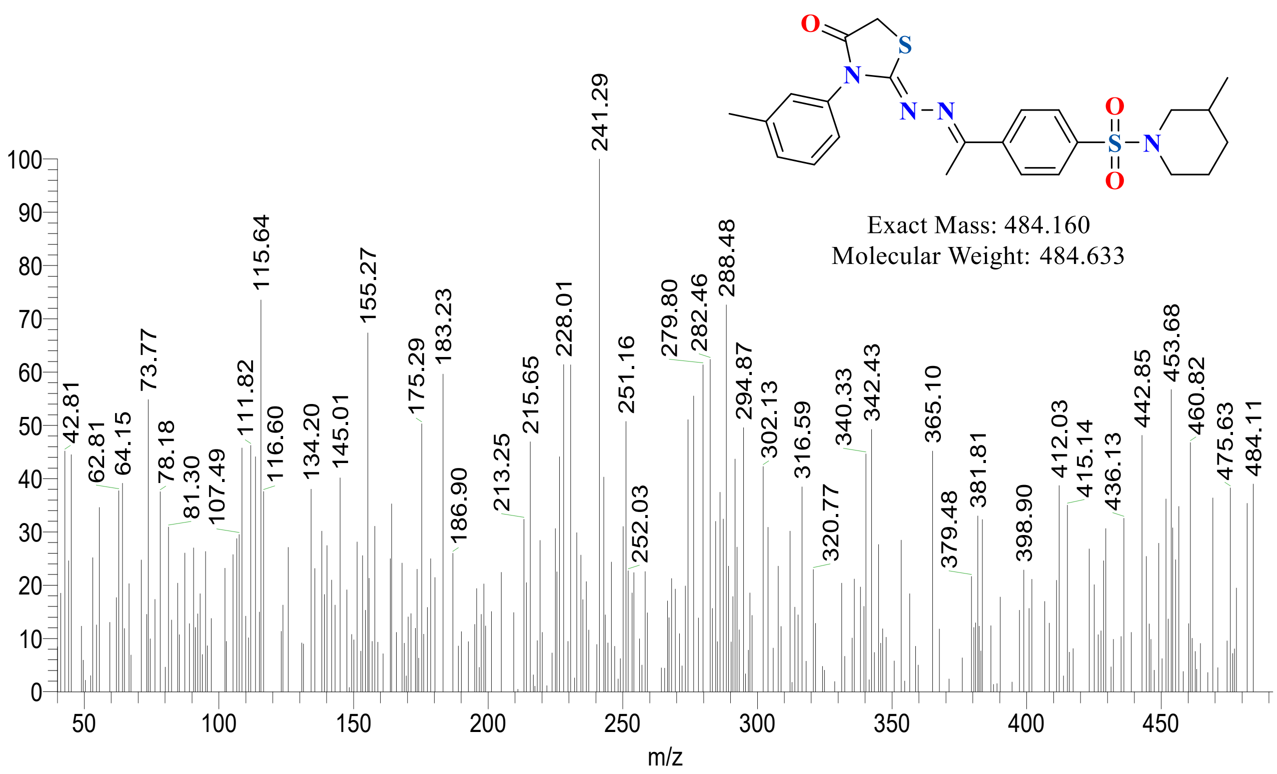

Fig S44: Mass spectrum of compound 15

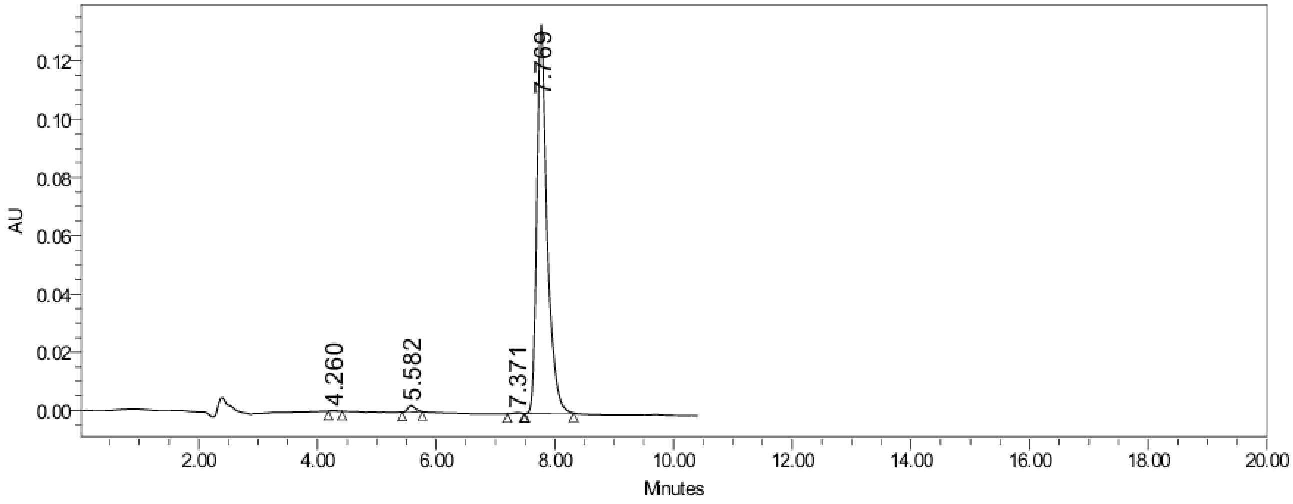

	RT	Area	% Area	USP Plate Count	USP Tailing	K Prime
1	4.260	1552	0.10	10810.99	1.48	3.06
2	5.582	17955	1.10	10006.05	1.20	4.32
3	7.371	2590	0.16	18595.82	0.85	6.02
4	7.769	1611485	98.65	10452.10	1.46	6.40

Fig S45: HPLC chromatogram of compound 15

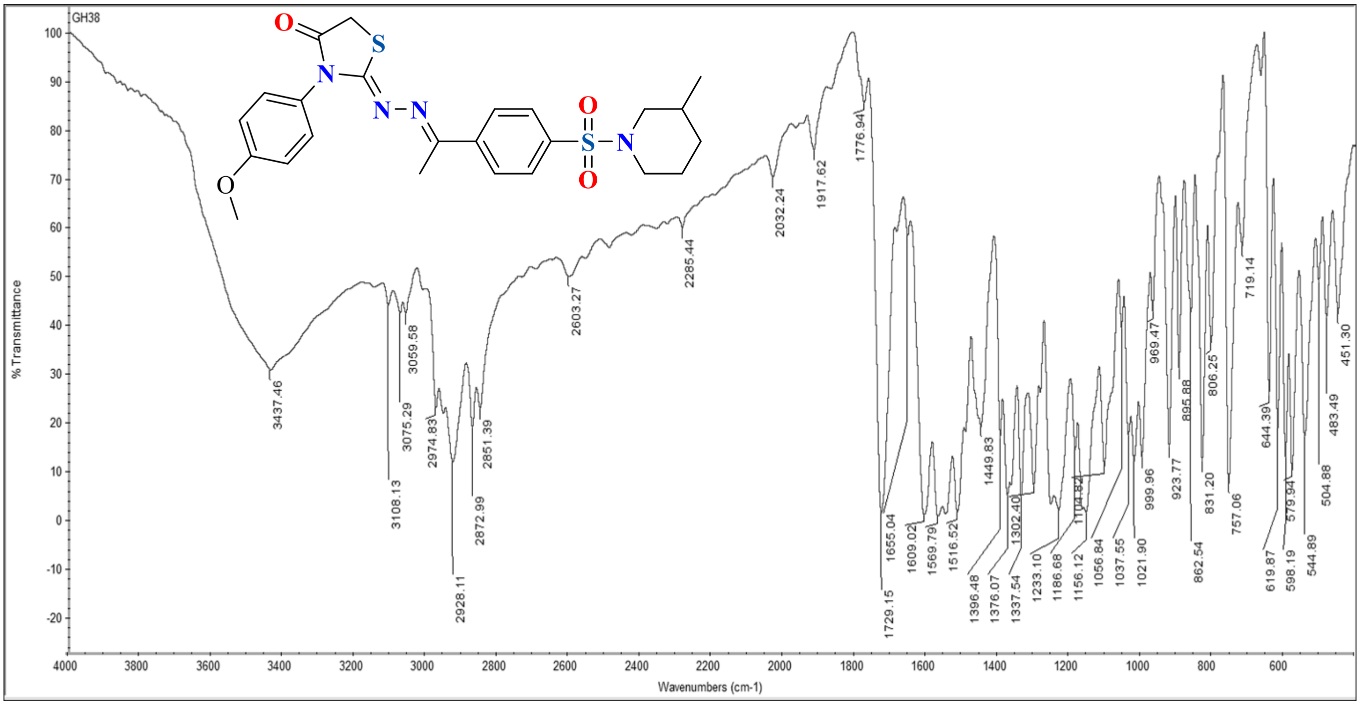

*Fig S46: FT-IR (*KBr, ν cm^{-1}) *of* compound *16.*

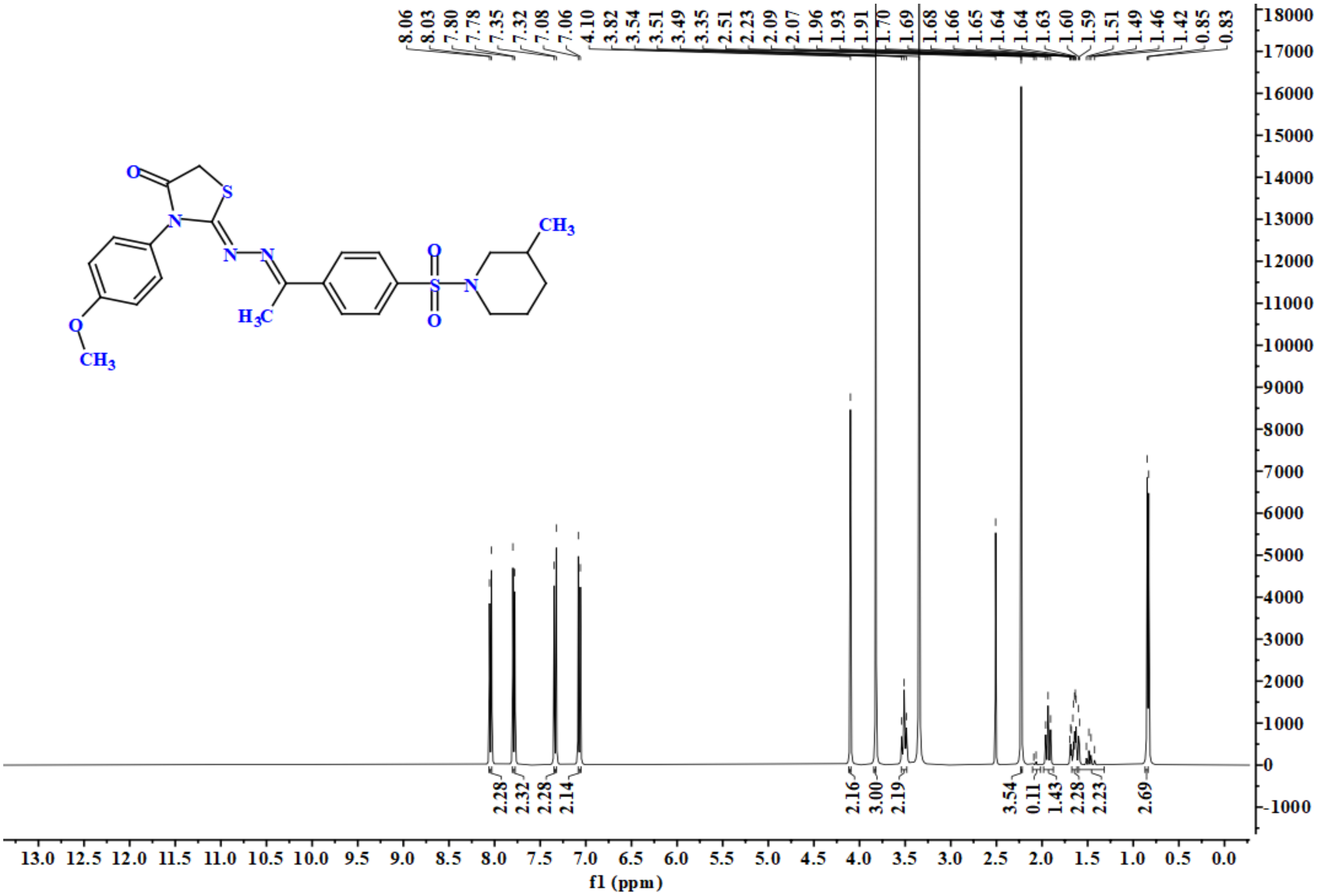

Fig S47: ¹H NMR (*DMSO-d$_6$) *of compound 16

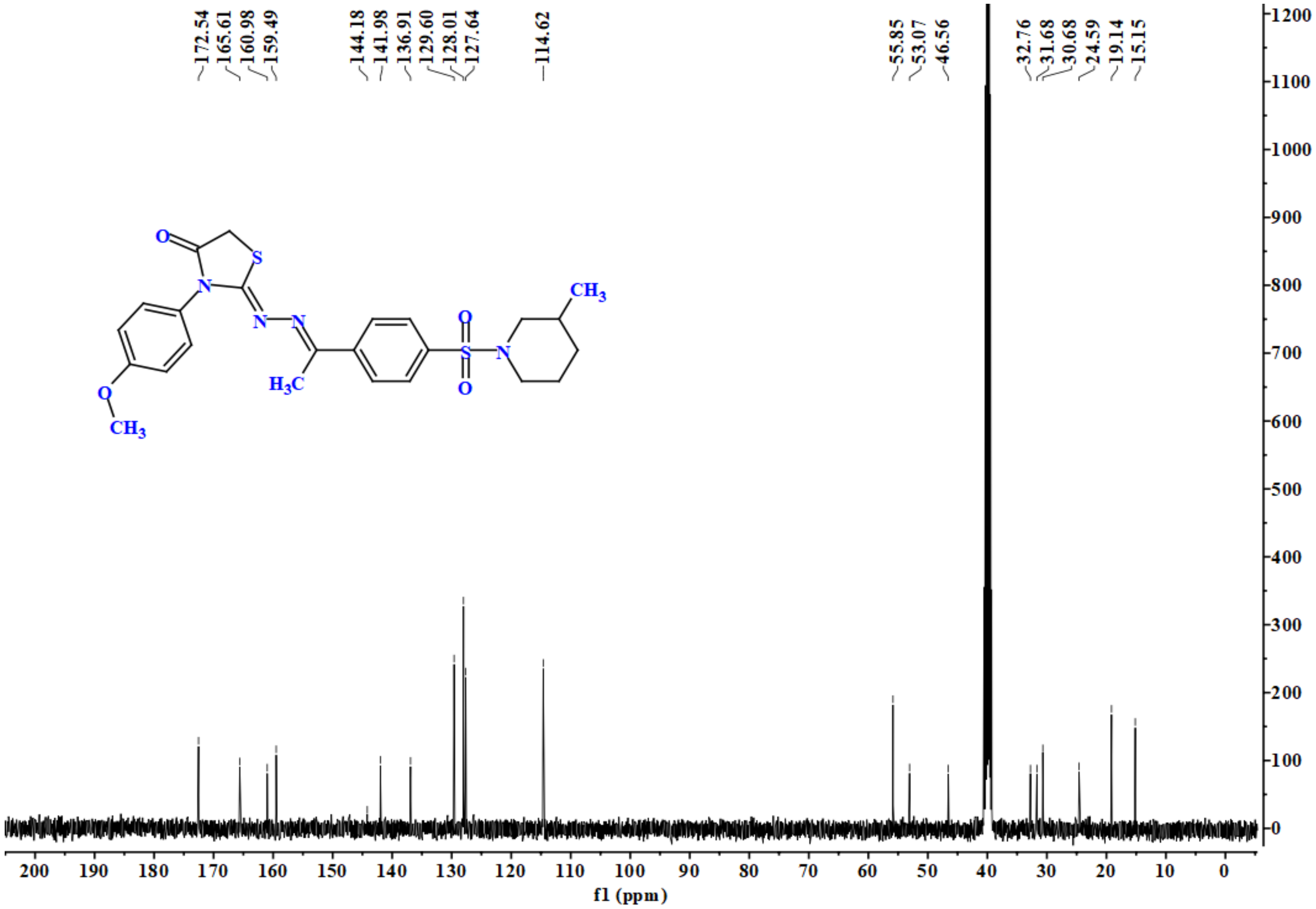

Fig S48: ^{13}C NMR (**DMSO-d6**) ***of*** **compound** ***16***

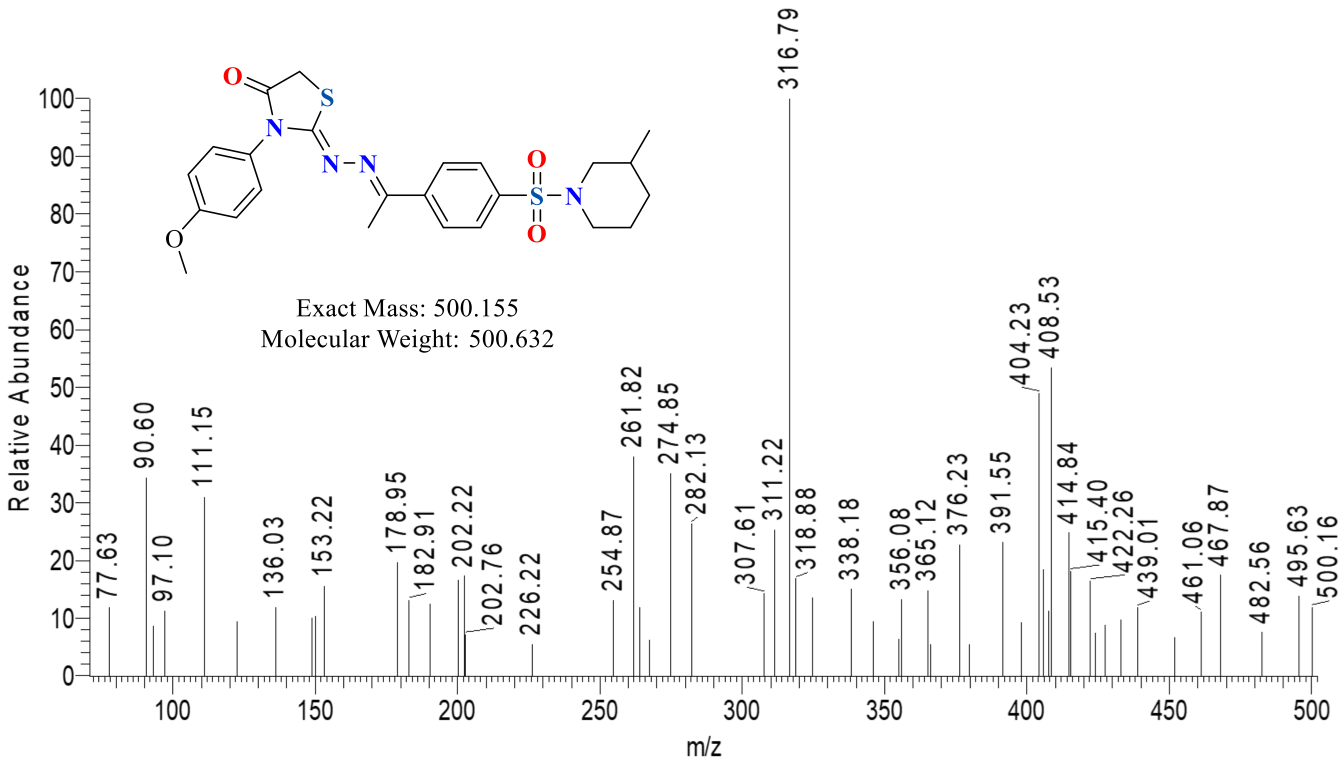

Fig S49: Mass spectrum of compound 16

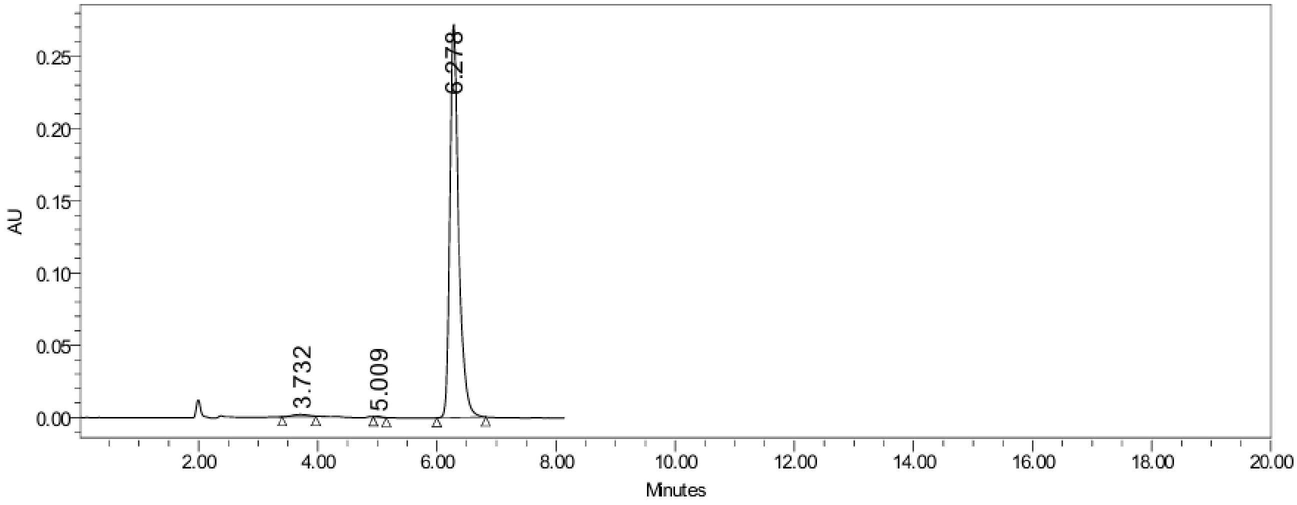

	RT	Area	% Area	USP Plate Count	USP Tailing	K Prime
1	3.732	8809	0.31	744.84	1.14	2.55
2	5.009	3635	0.13	15446.16	1.45	3.77
3	6.278	2794142	99.56	9654.85	1.41	4.98

Fig S50: HPLC chromatogram of compound 16

3. Biological activity (Doss response curve)

Compound 3

Con.	MCF-7Viability %	HCT-116Viability %	HEPG-2Viability %
100	16.43	26.22	22.81
50	29.33	38.04	32.74
25	43.49	51.8	48.54
12.5	58.92	74.27	63.97
6.25	72.36	80.43	77.65
0	100	100	100
IC_{50}	18.43±0.9	23.78±1.03	19.39±1.2

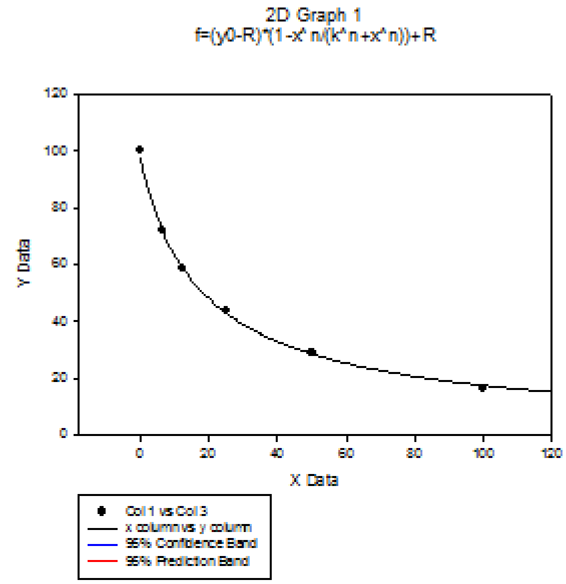

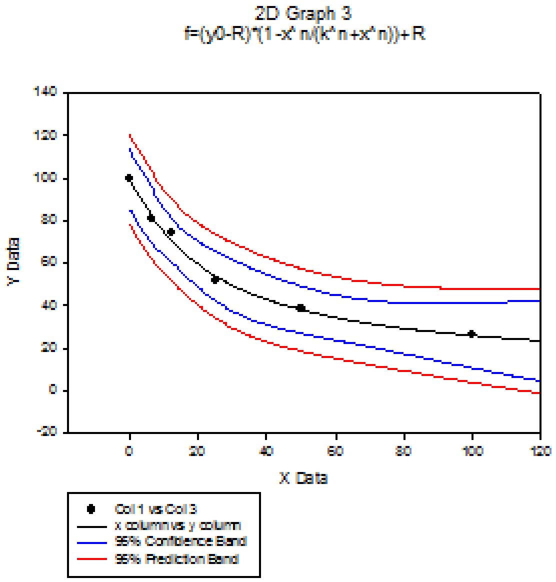
2D Graph 3
f=(y0-R)*(1-x^n/(k^n+x^n))+R
Y Data
X Data
Col 1 vs Col 3
x column vs y column
95% Confidence Band
95% Prediction Band

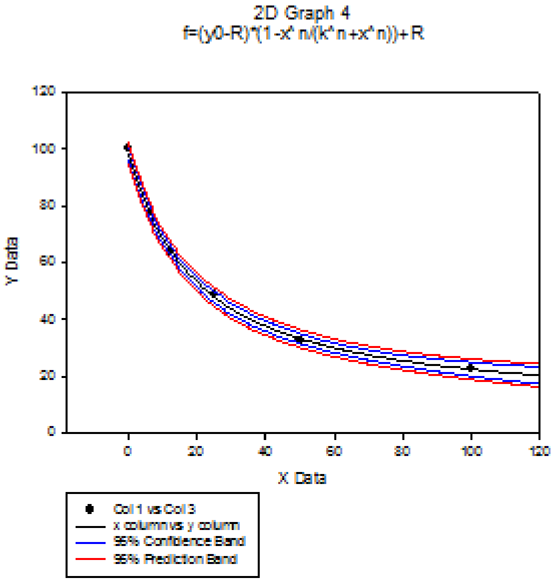
2D Graph 4
f=(y0-R)*(1-x^n/(k^n+x^n))+R
Y Data
X Data
Col 1 vs Col 3
x column vs y column
95% Confidence Band
95% Prediction Band

<u>Compound 9</u>

Con.	MCF-7Viability %	HCT-116Viability %	HEPG-2Viability %
100	12.62	13.9	17.34
50	22.56	23.75	31.12
25	37.64	41.28	46.25
12.5	48.44	53.54	58.54
6.25	61.29	68.43	71.16
0	100	100	100
IC_{50}	11.63±0.82	15.02± 0.92	19.06±0.85

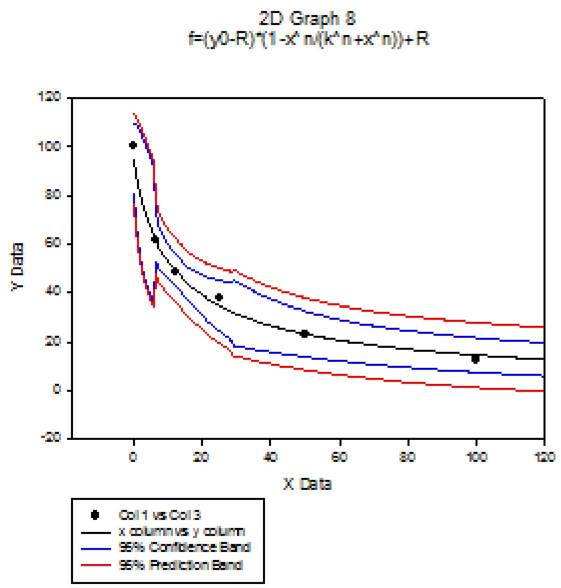

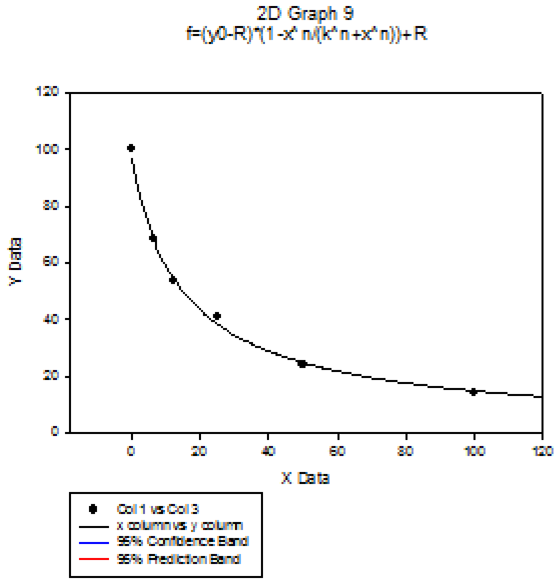
2D Graph 9
f=(y0-R)*(1-x^n/(k^n+x^n))+R
Y Data
X Data
Col 1 vs Col 3
x column vs y column
95% Confidence Band
95% Prediction Band

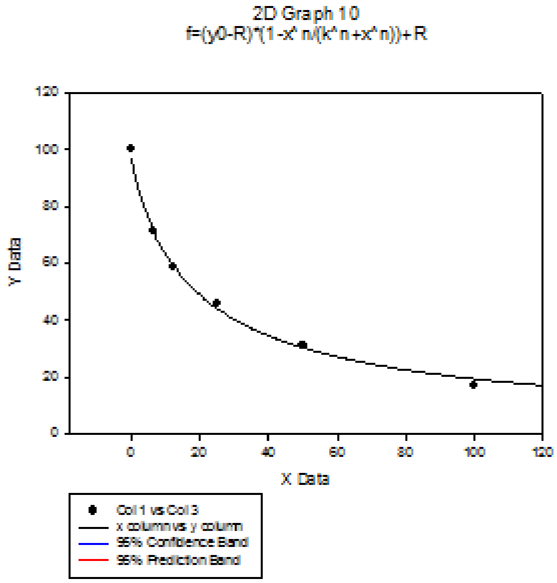
2D Graph 10
f=(y0-R)*(1-x^n/(k^n+x^n))+R
Y Data
X Data
Col 1 vs Col 3
x column vs y column
95% Confidence Band
95% Prediction Band

Compound 10

Con.	MCF-7Viability %	HCT-116Viability %	HEPG-2Viability %
100	26.51	22.1	20.17
50	40.71	33.52	30.5
25	56.24	51.03	48.12
12.5	67.14	65.82	60.31
6.25	80.13	75.73	70.6
0	100	100	100
IC_{50}	31.48± 0.04	24.66±0.89	20.0±0.8

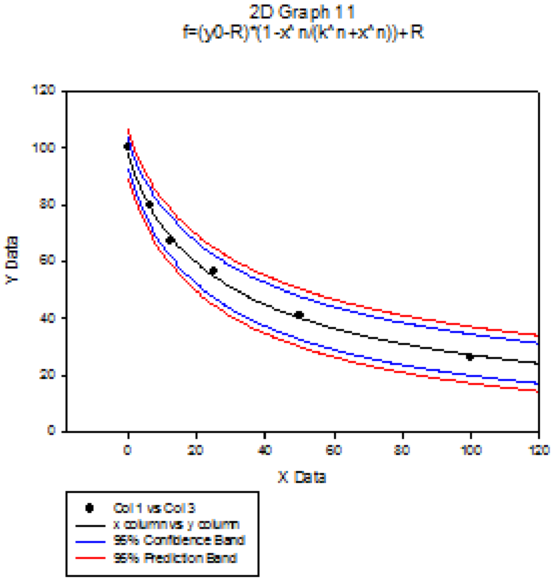

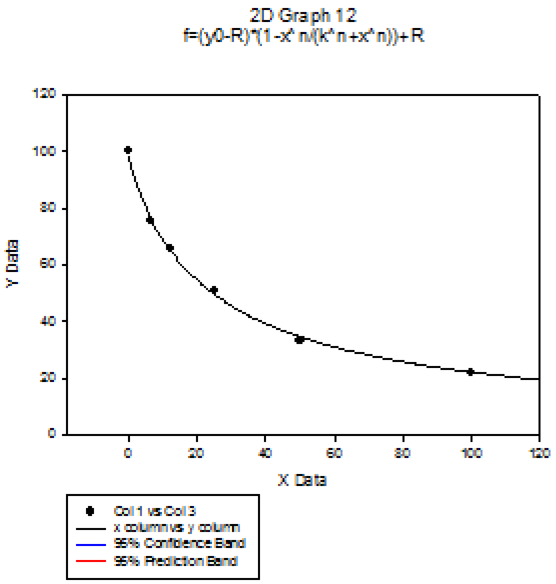

2D Graph 12
f=(y0-R)*(1-x^n/(k^n+x^n))+R
Y Data
X Data
Col 1 vs Col 3
x column vs y column
95% Confidence Band
95% Prediction Band

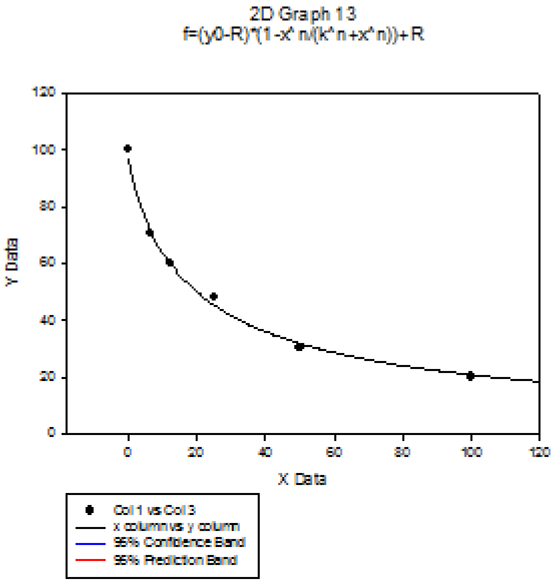

2D Graph 13
f=(y0-R)*(1-x^n/(k^n+x^n))+R
Y Data
X Data
Col 1 vs Col 3
x column vs y column
95% Confidence Band
95% Prediction Band

Compound 11

Con.	MCF-7Viability %	HCT-116Viability %	HEPG-2Viability %
100	23.56	16.9	15.92
50	35.8	29.11	27.93
25	50.47	41.84	41.09
12.5	63.49	55.18	52.62
6.25	78.61	71.63	70.2
0	100	100	100
IC_{50}	22.26±0.94	17.02± 0.87	15.73± 0.16

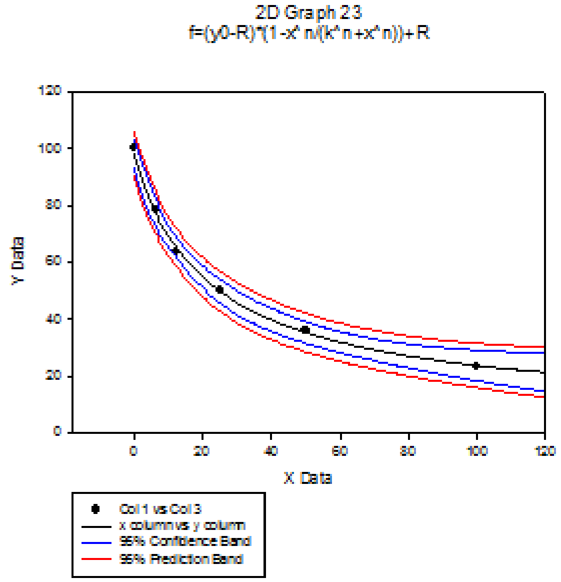

2D Graph 24
f=(y0-R)*(1-x^n/(k^n+x^n))+R

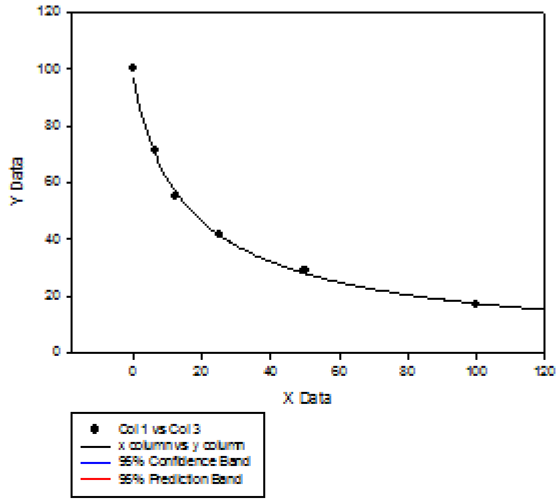

2D Graph 25
f=(y0-R)*(1-x^n/(k^n+x^n))+R

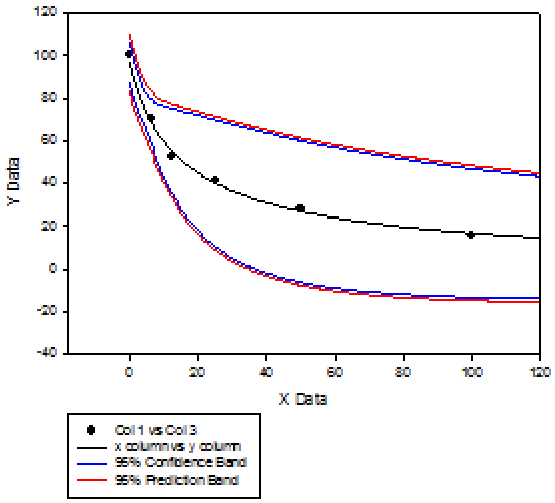

Compound 12

Con.	MCF-7Viability %	HCT-116Viability %	HEPG-2Viability %
100	14.81	16.7	13.93
50	27.16	30.59	23.81
25	35.44	41.26	31.72
12.5	47.57	50.19	42.18
6.25	61.29	65.89	55.92
0	100	100	100
IC_{50}	11.39± 0.18	14.41± 0.13	8.48± 0.7

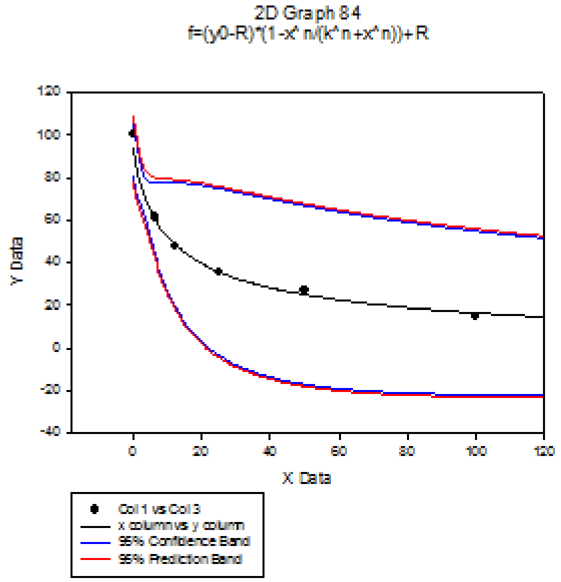

2D Graph 85

f=(y0-R)*(1-x^n/(k^n+x^n))+R

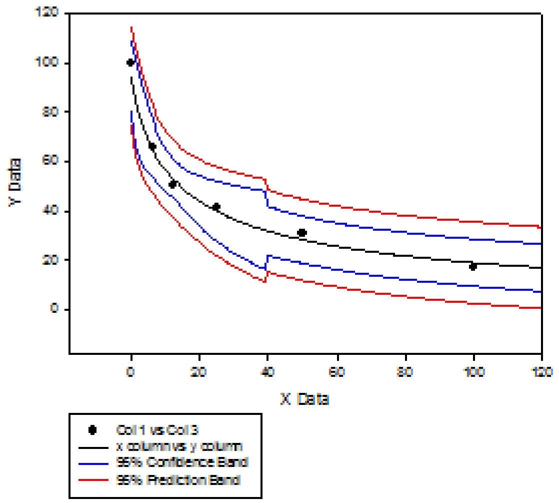

2D Graph 86

f=(y0-R)*(1-x^n/(k^n+x^n))+R

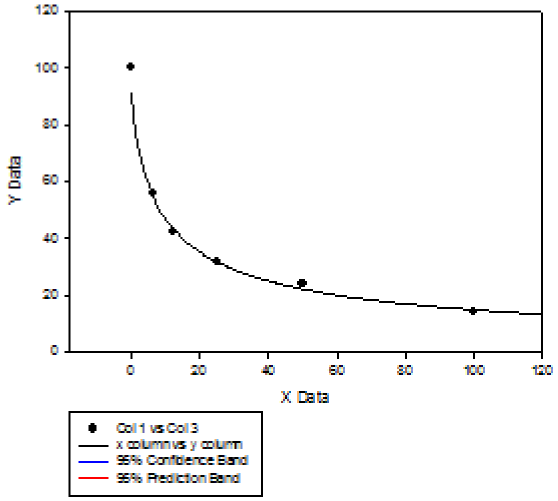

Compound 13

Con.	MCF-7Viability %	HCT-116Viability %	HEPG-2Viability %
100	12.15	7.16	5.77
50	24.53	18.16	15.38
25	34.17	32.21	28.7
12.5	52.18	48.74	44.61
6.25	65.33	61.87	59.25
0	100	100	100
IC_{50}	12.98±0.07	11.089± 0.23	9.60± 0.12

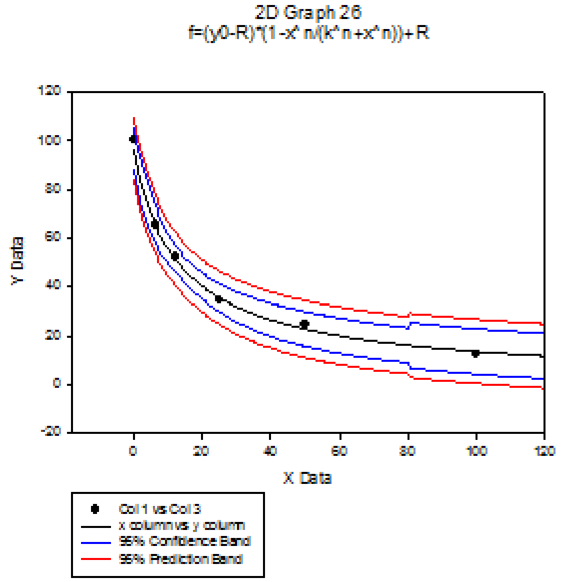

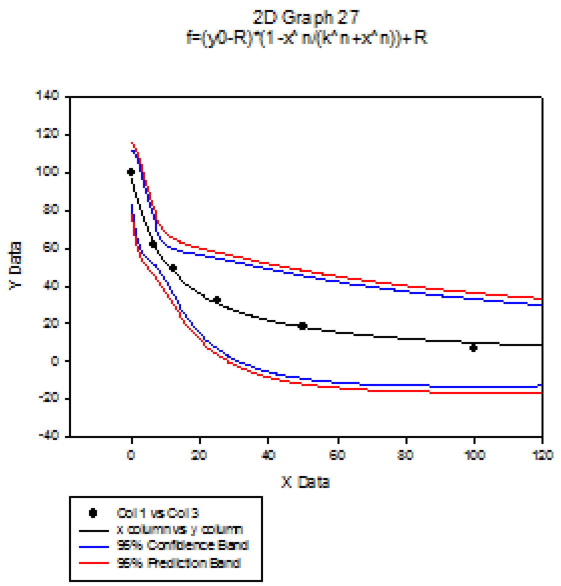
2D Graph 27
f=(y0-R)*(1-x^n/(k^n+x^n))+R
Y Data
X Data
Col 1 vs Col 3
x column vs y column
95% Confidence Band
95% Prediction Band

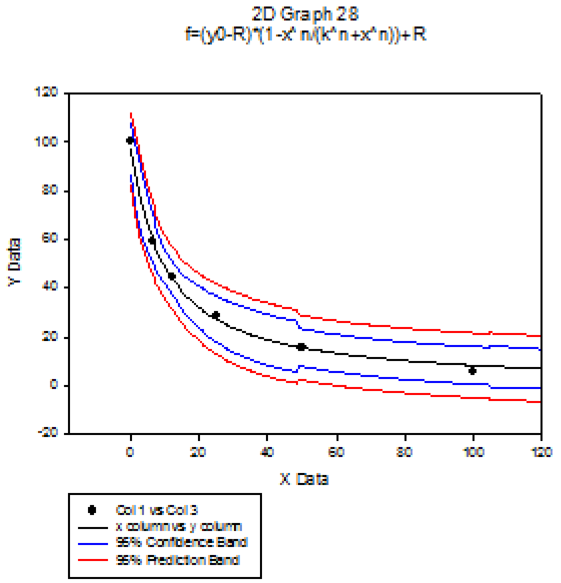
2D Graph 28
f=(y0-R)*(1-x^n/(k^n+x^n))+R
Y Data
X Data
Col 1 vs Col 3
x column vs y column
95% Confidence Band
95% Prediction Band

Compound 14

Con.	MCF-7Viability %	HCT-116Viability %	HEPG-2Viability %
100	17.38	15.85	13.22
50	33.11	28.99	25.03
25	44.69	42.89	36.5
12.5	58.24	55.34	47.39
6.25	67.14	62.55	56.27
0	100	100	100
IC_{50}	17.91± 0.77	14.84± 07.5	10.05± 0.7 1

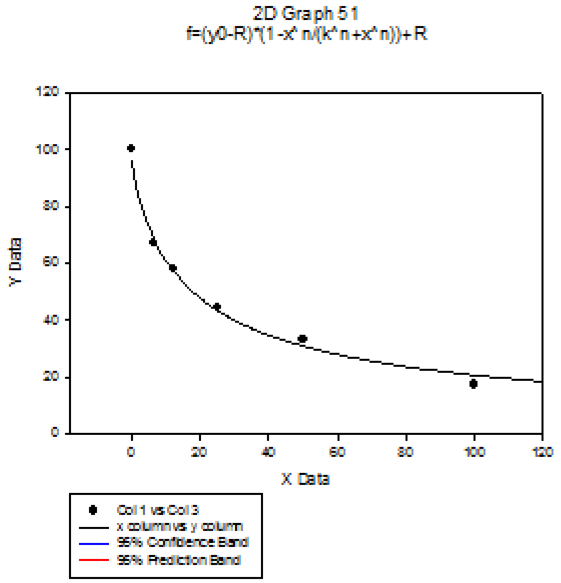

2D Graph 52
f=(y0-R)*(1-x^n/(k^n+x^n))+R

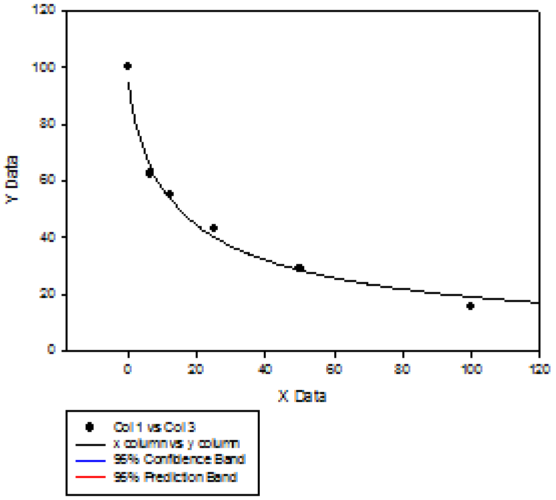

2D Graph 53
f=(y0-R)*(1-x^n/(k^n+x^n))+R

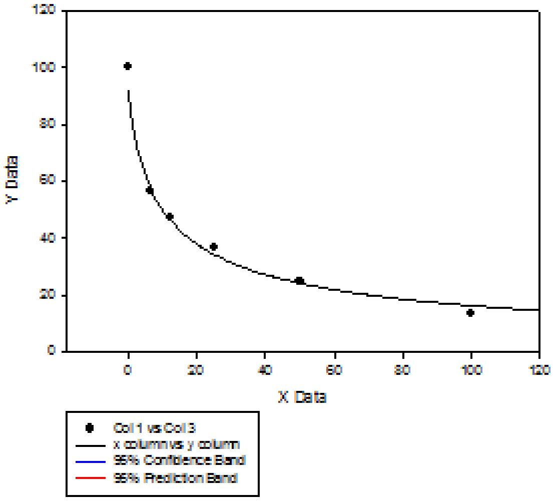

Compound 15

Con.	MCF-7Viability %	HCT-116Viability %	HEPG-2Viability %
100	18.37	15.31	7.83
50	30.94	28.72	14.94
25	39.19	36	27.55
12.5	51.67	47.11	38.89
6.25	64.17	62.75	50.19
0	100	100	100
IC_{50}	14.02± 0.12	11.90± 0.25	6.79± 0.11

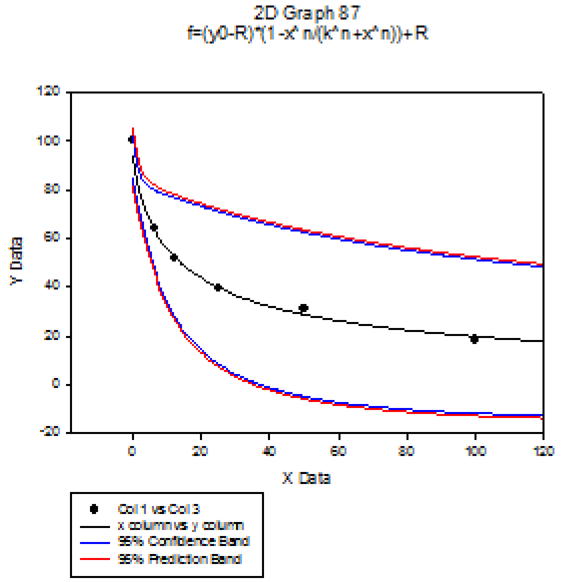

2D Graph 88
f=(y0-R)*(1-x^n/(k^n+x^n))+R

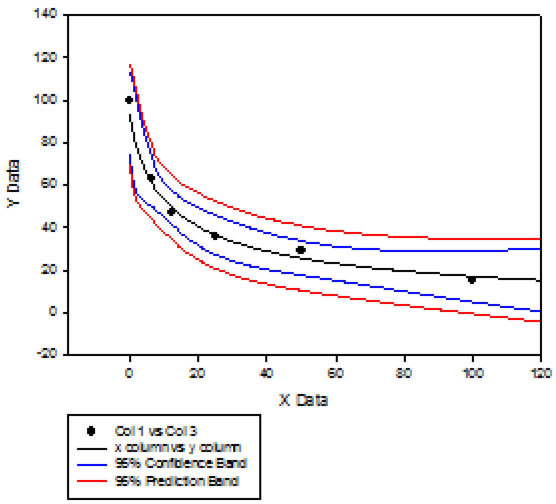

2D Graph 89
f=(y0-R)*(1-x^n/(k^n+x^n))+R

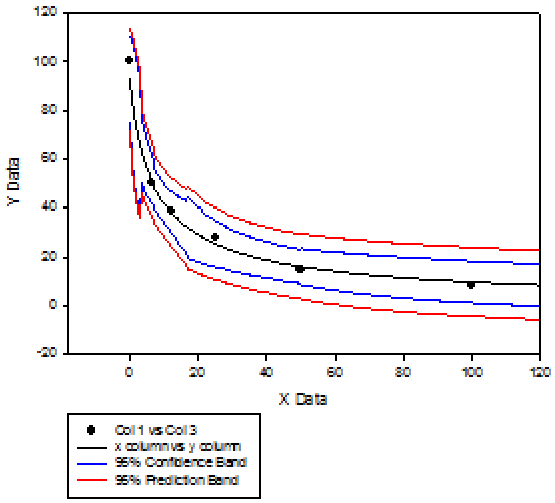

Compound 16

Con.	MCF-7Viability %	HCT-116Viability %	HEPG-2Viability %
100	2.08	2.57	2.85
50	10.36	11.02	10.94
25	14.72	16.51	17.99
12.5	25.49	27.42	31.75
6.25	38.25	41.19	42.36
0	100	100	100
IC_{50}	3.78± 0.15	4.27 ± 0.52	4.79± 0.81

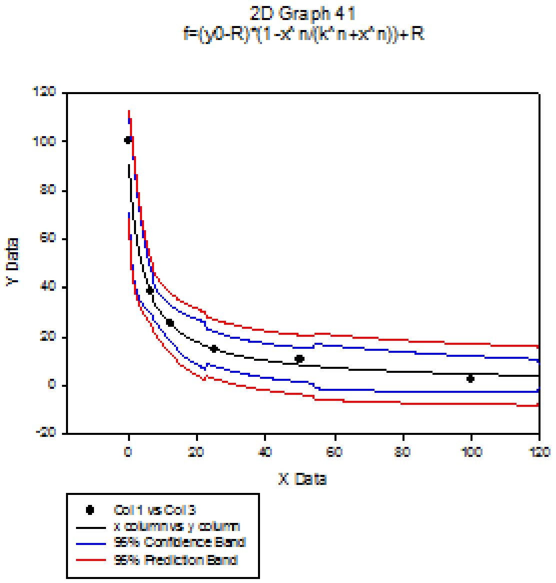

2D Graph 44
f=(y0-R)*(1-x^n/(k^n+x^n))+R

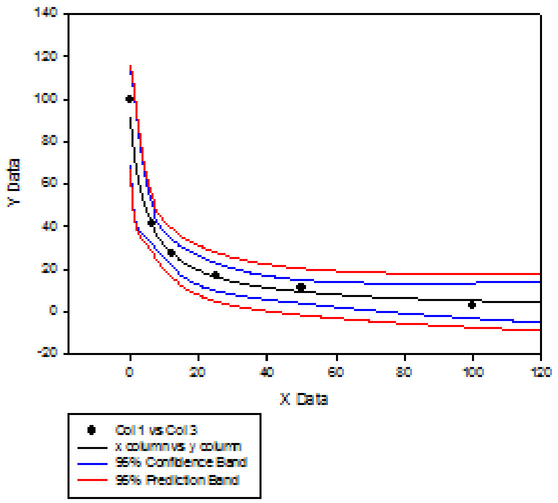

2D Graph 43
f=(y0-R)*(1-x^n/(k^n+x^n))+R

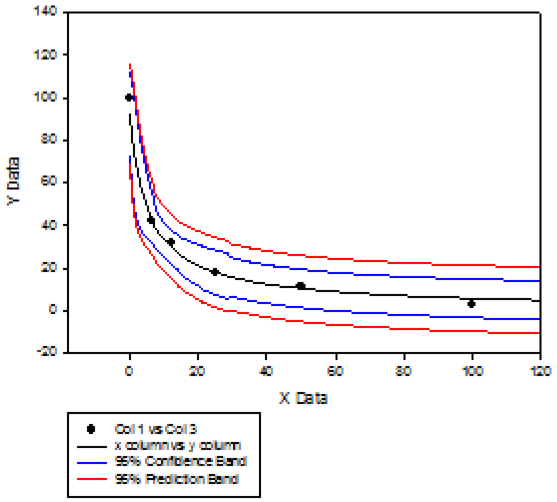

Supplement: Supplementary file 1 — Supplementary Material 1 [file 41598_2026_44109_MOESM1_ESM.pdf]
